# A TGF-**β**1/LEF1/**β**-catenin/JLP network motif regulates autophagy and tubule injury in renal fibrosis

**DOI:** 10.1172/jci.insight.196835

**Published:** 2026-01-08

**Authors:** Chen Li, Meng Zhang, Maoqing Tian, Zeyu Tang, Yuying Hu, Yuyu Long, Xiaofei Wang, Liwen Qiao, Jiefei Zeng, Yujuan Wang, Xinghua Chen, Cheng Chen, Xiaoyan Li, Lu Zhang, Huiming Wang

**Affiliations:** 1 Department of Nephrology, Renmin Hospital of Wuhan University, Wuhan, China.; 2 Hubei Provincial Clinical Research Center for Kidney Disease, Wuhan, China.; 3 Department of Internal Medicine, and Department of Biochemistry and Molecular Biology, Mayo Clinic, Rochester, Minnesota, USA.

**Keywords:** Cell biology, Nephrology, Autophagy, Chronic kidney disease, Fibrosis

## Abstract

Sustained injury to renal tubular epithelial cells (TECs), driven by excessive autophagy, is a critical mechanism underlying kidney fibrosis. Our previous work identified JLP — a TEC-expressed scaffolding protein — as an endogenous antifibrotic factor that counteracts TGF-β1–induced autophagy and fibrogenesis. However, the mechanism underlying JLP downregulation in renal fibrosis remains unclear. Here, we delineated a TGF-β1/LEF1/β-catenin/JLP axis that governs TEC autophagy through a dichotomous regulatory circuit. Under physiological conditions, low levels of β-catenin and LEF1 with minimal nuclear localization permitted normal JLP expression, which in turn maintained autophagy in check. In contrast, during renal injury, TGF-β1 promoted the expression and nuclear translocation of β-catenin and LEF1, which together suppressed JLP transcription. This loss of JLP-mediated inhibition led to unchecked autophagy and exacerbated fibrotic damage. Analyses of kidney tissues from patients with CKD, murine fibrotic kidneys, and cultured HK-2 cells confirmed consistent JLP downregulation accompanied by upregulation and nuclear accumulation of LEF1 and β-catenin. Therapeutic intervention using the β-catenin/LEF1 inhibitor iCRT3 or LEF1-targeted silencing in murine fibrosis models restored JLP expression, attenuated TEC autophagy, and ameliorated renal fibrosis. These findings revealed an autoregulatory circuit controlling TEC autophagy and fibrogenesis, and supported LEF1 and β-catenin as potential therapeutic targets in CKD.

## Abstract 2

This study identifies LEF1 as a potential therapeutic target for mitigating kidney fibrosis in chronic kidney disease.

## Graphical abstract

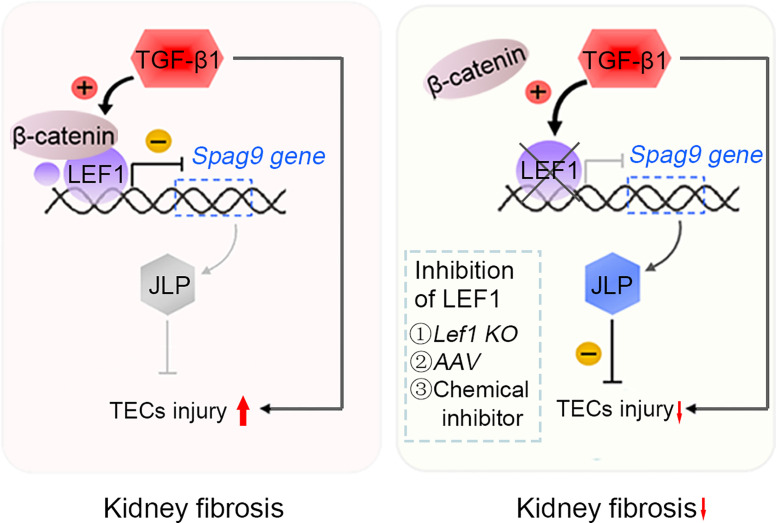

## Introduction

Tubulointerstitial fibrosis is a hallmark of progressive chronic kidney disease (CKD), characterized by tubular atrophy and accumulation of extracellular matrix (ECM) in renal tissues (1–4). Renal tubular epithelial cells (TECs), the major components of kidney tissue, are highly susceptible to injury from hypoxia, proteinuria, and toxins (5–8). Injured TECs undergo structural and phenotypic changes, adopting inflammatory and fibrogenic tributes that drive fibrosis progression (1, 5, 9).

Autophagy, a cellular homeostatic mechanism, maintains TEC integrity under both physiological and pathological conditions (10). While it protects against acute renal injury, persistent TEC autophagy promotes maladaptive repair by inducing tubular degeneration and profibrotic phenotypes, accelerating renal dysfunction (11–14). However, the molecular mechanism underlying relentless TEC autophagy in renal fibrosis remains unclear.

Transforming growth factor-β1 (TGF-β1), a key driver of CKD, promotes tubular injury and fibrosis (15). However, despite its well-established pathogenic role, attempts to inhibit TGF-β1 in humans have not yielded successful outcomes (16). Therefore, identifying alternative and effective therapeutic targets is crucial for preventing the onset and progression of CKD. We previously identified JNK-associated leucine zipper protein (JLP, encoded by the *SPAG9* gene [sperm-associated antigen 9]), a scaffolding protein that plays a crucial role in orchestrating several cellular events, such as proliferation, apoptosis, autophagy, migration, and epithelial-mesenchymal transition (EMT) (17, 18), and serves as an endogenous antifibrotic factor in the kidney with predominant expression in renal tubules, particularly the proximal segment of the nephron (15, 19). JLP is a fundamental coordinator of cell vesicle transport, facilitating the movement of organelles and other cellular components within the cell and may regulate lysosome localization and autophagy (20, 21). Recent studies have shown that JLP is involved in regulating fibrotic diseases through counteracting the profibrotic effects of TGF-β1 (15, 22, 23). These findings suggest a potential therapeutic approach of retaining the JLP expression level other than direct TGF-β1 inhibition in preventing renal fibrosis.

Lymphoid enhancer-binding factor 1 (LEF1), a member of the T cell factor (TCF)/LEF1 family, is an important downstream mediator of the Wnt/β-catenin signaling pathway. A substantial elevation in LEF1 levels has been observed in patients with idiopathic pulmonary fibrosis and cardiac fibrosis (24, 25). As a high-mobility-group domain–containing transcription factor, LEF1 regulates the expression of canonical Wnt target genes (26). A recent study demonstrated that LEF1 regulates the transcription of JLP in endothelial cells (27). However, the role of LEF1 regulates JLP in tubular cells and renal fibrosis has not been investigated.

In the present study, we demonstrated that LEF1 expression is excessively upregulated in fibrotic TECs from patients with CKD and in murine models. LEF1-mediated inhibition of JLP prolonged autophagy activation in TECs and exacerbated fibrosis. Critically, the inhibition of LEF1 mitigated renal fibrosis, highlighting its therapeutic potential. We propose that the TGF-β1/LEF1/β-catenin/JLP axis is a regulator of TEC autophagy and renal fibrosis, offering a molecular target for therapeutic interventions in CKD.

## Results

### JLP is the direct downstream target of LEF1.

**Figure 1:**
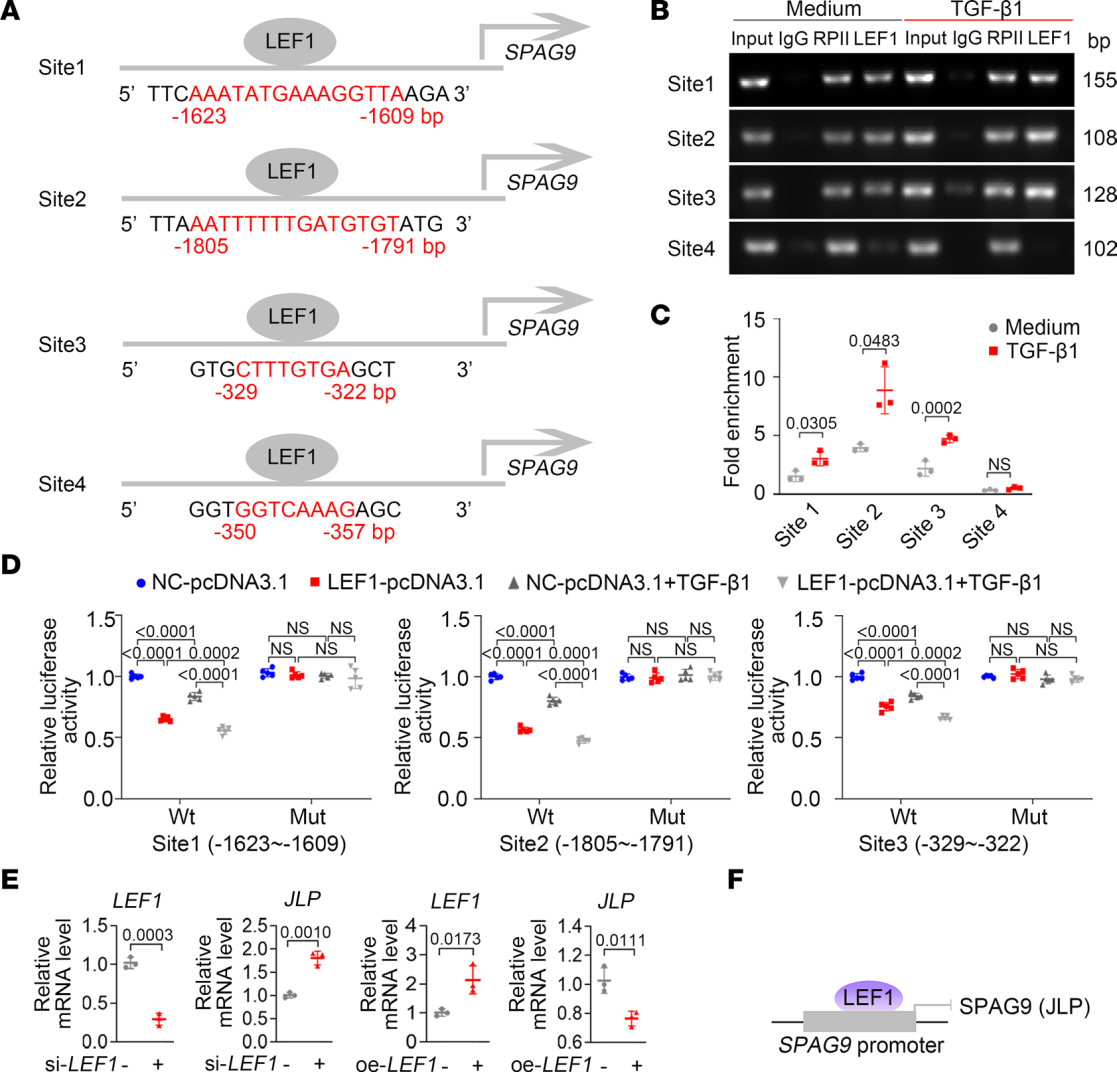
LEF1 acts as a transcription factor to inhibit JLP gene expression. (**A**) The 4 putative 4 LEF1 binding sites in the JLP gene (*SPAG9*) promoter region identified by JASPAR. (**B**) PCR amplification was carried out with DNA fragments that were immunoprecipitated by anti-LEF1 (IP), anti-IgG (negative control), and anti–RNA polymerase II (RPII, positive control), and total DNA fragment (Input). (**C**) ChIP-qPCR was performed to verify LEF1 binding to the promoter of the *SPAG9* gene in HK-2 cells (*n* = 3 independent experiments). (**D**) Relative luciferase activities associated with the wild-type (Wt) and site-mutated (Mut) LEF1-binding sequences in the *SPAG9* gene promoter in LEF1-overexpressing HEK-293T cells (*n* = 5 independent experiments). (**E**) qRT-PCR analysis of *SPAG9* and *LEF1* expression in HK-2 cells from the indicated groups. Cells were transfected with either *LEF1* siRNA or control siRNA (left panel), or with either pcDNA3.1 (oe-Ctrl) or LEF1-pcDNA3.1 (oe-*LEF1*) plasmid (right panel) (*n* = 3 independent experiments). (**F**) Schematic representation of LEF1 binding to the promoter region of the *SPAG9* gene, regulating JLP expression. Data are presented as mean ± SD. Two-tailed, unpaired Student’s *t* test (**C** and **E**) and 1-way ANOVA followed by Tukey’s multiple-comparison test (**D**) were used for statistical analysis. NS, no significant difference.

First, using JASPAR software (https://jaspar.elixir.no/), we identified LEF1 as a potential transcriptional regulator of JLP (*SPAG9* gene), with 4 predicted binding regions on the *SPAG9* promoter (Figure 1A). Previous studies demonstrated that LEF1 binds to the *SPAG9* promoter in HUVECs to regulate its expression (27). To confirm the physical interaction between LEF1 and the *SPAG9* promoter in renal TECs, we performed chromatin immunoprecipitation (ChIP) assays in HK-2 cells (Supplemental Figure 1A; supplemental material available online with this article; https://doi.org/10.1172/jci.insight.196835DS1). Our results confirmed LEF1 binding to 3 specific regions of the *SPAG9* promoter (–322 to –329 bp, –1609 to –1623 bp, and –1791 to –1805 bp) in HK-2 cells (Figure 1, B and C). Three LEF1-binding motifs, AAATATGAAAGGTTA, AATTTTTTGATGTGT, and CTTTGTGA, were identified within the *SPAG9* promoter (Figure 1B). Notably, in the presence of TGF-β1, LEF1 exhibited increased binding to the *SPAG9* promoter (Figure 1, B and C). In addition, we performed luciferase reporter assays to further validate gene expression. The results demonstrated that LEF1 markedly decreased JLP promoter activity, whereas TGF-β1 stimulation enhanced LEF1 binding to the JLP promoter (Figure 1D). The regulatory relationship between LEF1 and JLP was further validated by examining the mRNA level of JLP in HK-2 cells with altered LEF1 expression. As anticipated, LEF1 knockdown increased JLP expression, while overexpression reduced it (Figure 1, E and F and Supplemental Figure 1, B and C), establishing LEF1 as a *SPAG9* repressor.

### LEF1 and JLP exhibit an inverse correlation in fibrotic kidneys.

**Figure 2:**
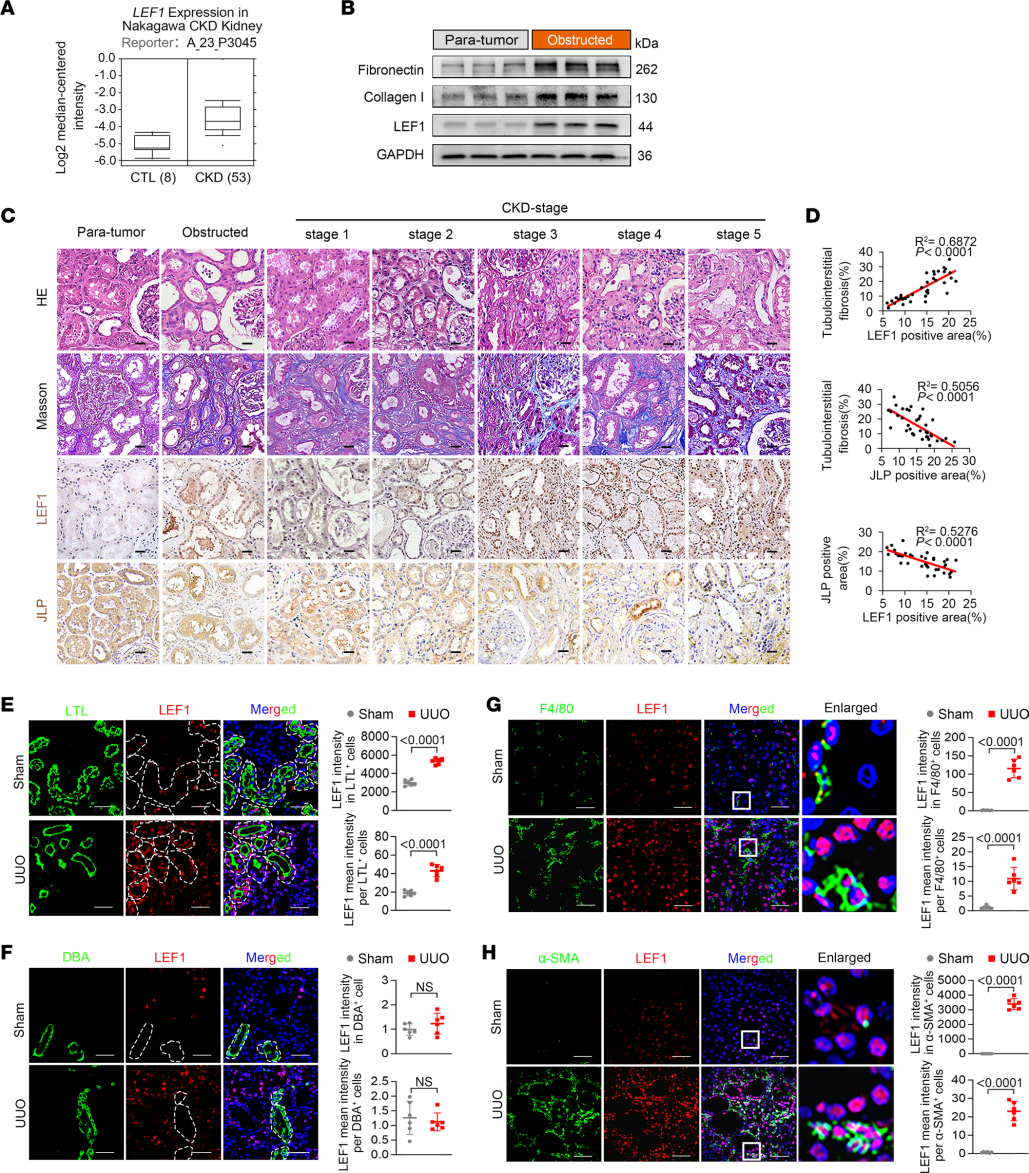
JLP expression is inversely correlated with LEF1 level in fibrotic kidneys and TGF-β1–treated TECs. (**A**) *LEF1* mRNA expression data were extracted from the Nephroseq database (https://www.nephroseq.org). Boxes bounds represent the interquartile range (25th to 75th percentiles), the horizontal line within each box indicates the median, whiskers extend to 1.5 × the interquartile range, and individual dots denote outliers beyond this range. **B**) Western blotting analysis of fibronectin, collagen I, and LEF1 protein levels (*n* = 6 per group). (**C**) Images of H&E, Masson’s trichrome, and immunohistochemical staining of kidney sections from paratumor kidney tissue of patients with renal carcinoma and from renal specimens of patients with obstructive nephropathy and CKD. Scale bars: 50 μm. (**D**) Correlations between LEF1 expression and tubulointerstitial fibrosis, JLP expression and tubulointerstitial fibrosis, and renal LEF1 expression and JLP expression are shown (*n* = 38). (**E**–**H**) Representative dual-color immunofluorescence images of mouse kidney sections stained for LEF1 (red) and cell-type-specific markers: LTL (proximal tubular cells), DBA (collecting ducts), F4/80 (macrophages), and α-SMA (pericytes and myofibroblasts). Scale bars: 50 μm. (**G** and **H**)The total original magnification for the enlarged images is ×3,000. Quantification of LEF1 fluorescence intensity in marker-positive cells and mean fluorescence intensity per marker-positive cell was performed using ImageJ (*n* = 6 mice per group; 5 images were analyzed per sample). Data are presented as mean ± SD. Linear regression analysis (**D**) and 2-tailed, unpaired Student’s *t* test (**E**–**H**) were used for statistical analysis.

To evaluate the clinical relevance of LEF1 in kidney fibrosis, we first analyzed LEF1 mRNA expression using the Nephroseq database (https://nephroseq.org/). In the “Nakagawa CKD” dataset, LEF1 expression was observably upregulated in kidney tissues from patients with CKD (*n* = 53) compared with healthy controls (*n* = 8) (Figure 2A). Consistently, by examining the expression of LEF1 in kidney samples from patients with renal fibrosis (obstructive nephropathy, CKD patients) and without renal fibrosis (paratumor kidney tissue from patients with renal carcinoma), we found that LEF1 was weakly expressed in control human kidneys but remarkably upregulated in kidneys from obstructive nephropathy (Figure 2, B and C). LEF1 upregulation was accompanied by increased fibronectin and collagen I accumulation (Figure 2B and Supplemental Figure 2A). We also examined the expression of LEF1 and JLP in kidney tissues from patients with CKD stages 1–5. Immunohistochemical analysis revealed that LEF1 expression increased progressively with CKD stage, whereas JLP expression decreased (Figure 2C). Correlation analysis showed a positive association between LEF1 expression and tubulointerstitial fibrosis scores, and a negative association between JLP expression and tubulointerstitial fibrosis scores. Furthermore, LEF1 expression was positively correlated with serum creatinine and blood urea nitrogen levels, but negatively correlated with estimated glomerular filtration rate (eGFR) (Figure 2D and Supplemental Figure 2B). In contrast, JLP expression showed the opposite pattern (Figure 2D and Supplemental Figure 2B).

To determine LEF1 expression in various renal cell types, dual immunofluorescent staining was performed using markers for proximal tubules (*Lotus* *tetragonolobus* lectin, LTL), collecting ducts (*Dolichos* *biflorus* agglutinin, DBA), macrophages (F4/80), and fibroblasts (α-SMA) in the mouse kidneys. Compared with sham-operated controls, LEF1 expression was markedly elevated following unilateral ureteral obstruction (UUO), predominantly in LTL-positive TECs (Figure 2, E–H). We next assessed the expression patterns of LEF1 and JLP in 2 mouse models of kidney fibrosis: UUO and unilateral renal ischemia-reperfusion injury (uIRI). Mice were sacrificed at 14 days after UUO or 28 days after uIRI for histological and molecular analyses of fibrotic injury. In UUO mouse models, LEF1 mRNA and protein levels increased, while JLP decreased (Supplemental Figure 3, A and B). H&E and Masson’s trichrome staining confirmed tubular damage and collagen deposition in kidneys (Supplemental Figure 3C). Nuclear LEF1 localization in TECs was inversely correlated with JLP expression (Supplemental Figure 3, D and E). Similar changes were observed in uIRI-induced renal fibrosis mouse models (Supplemental Figure 4, A–F). In addition, TGF-β1–treated HK-2 cells mirrored these findings (Supplemental Figure 4, G–I).

### LEF1 drives TEC injury via JLP-dependent autophagy.

**Figure 3:**
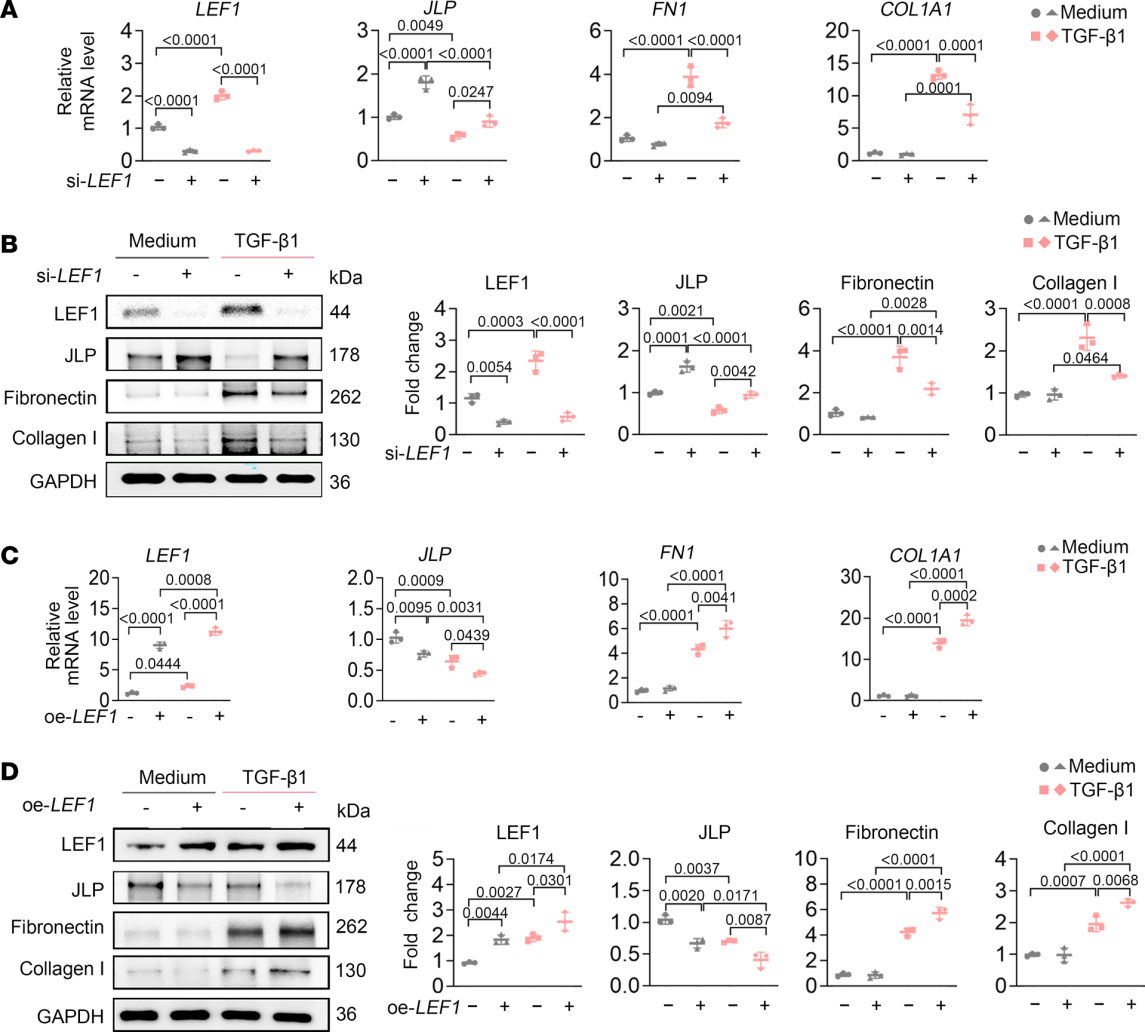
LEF1 exerts profibrotic effects on TECs under TGF-β1 stimulation. (**A** and **B**) qRT-PCR analysis (**A**) and immunoblotting analysis (**B**) were performed to detect the expression of LEF1, JLP, fibronectin, and collagen I in HK-2 cells with either *LEF1* siRNA or control siRNA, followed by TGF-β1 stimulation (*n* = 3 independent experiments). (**C** and **D**) mRNA (**C**) and protein (**D**) levels of LEF1, JLP, fibronectin, and collagen I in HK-2 cells transfected with LEF1 overexpression plasmid or control vector following TGF-β1 treatment (*n* = 3 independent experiments). Data are presented as mean ± SD and 1-way ANOVA followed by Tukey’s multiple-comparison test was used for statistical analysis.

The above findings prompted further investigation into the functional relationship between LEF1 and TEC injury. HK-2 cells were transfected with *LEF1* siRNA or control siRNA and subsequently treated with TGF-β1 for 24 hours. *LEF1* siRNA attenuated TGF-β1–induced fibrosis markers (fibronectin, collagen I) and restored JLP, at both the mRNA (Figure 3A) and protein levels (Figure 3B). Conversely, *LEF1* overexpression exacerbated these effects (Figure 3, C and D). Collectively, these results demonstrate that LEF1 mediates TGF-β1–driven upregulation of fibronectin and collagen I in TECs, thereby contributing to renal fibrosis.

**Figure 4:**
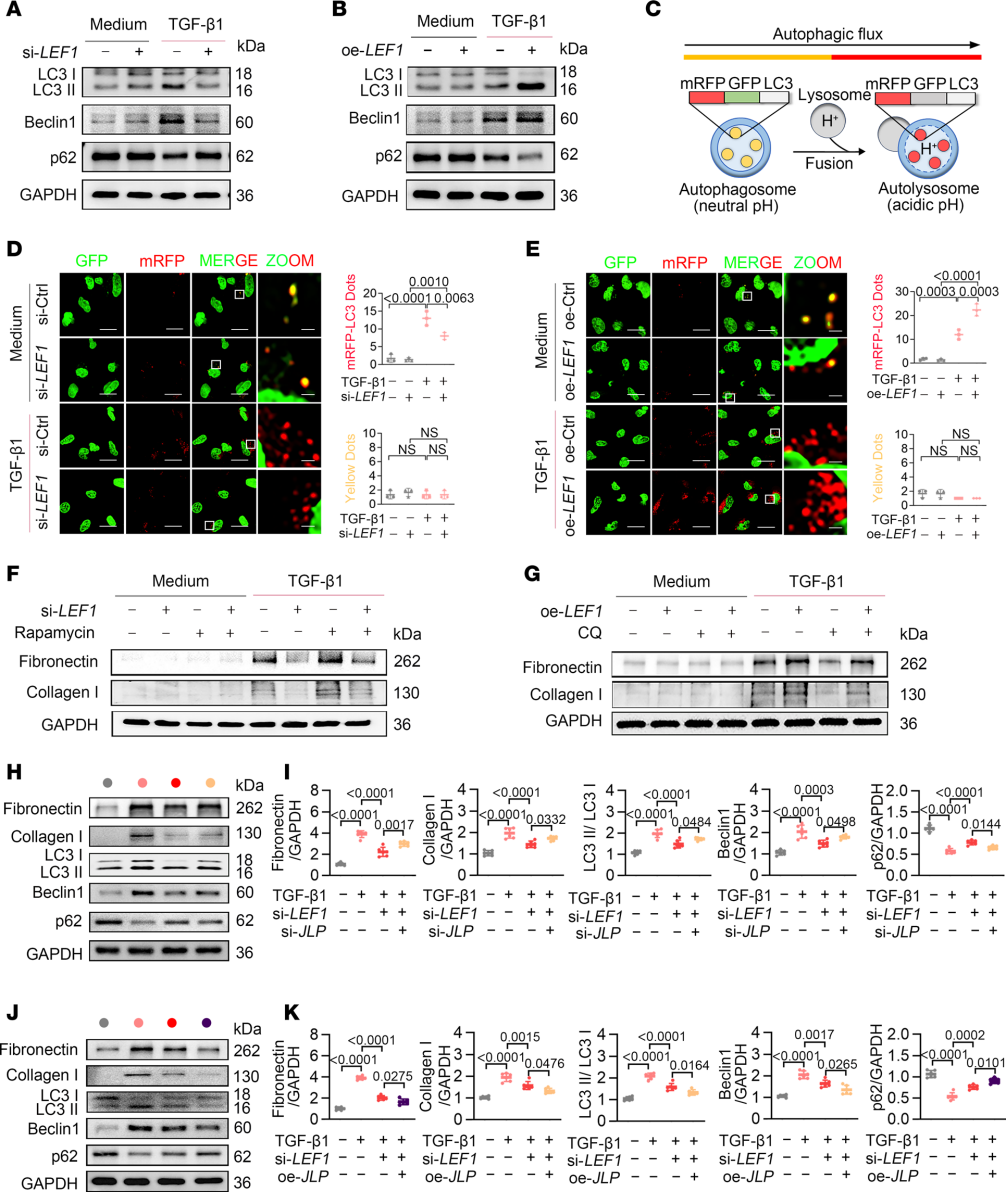
LEF1 regulates autophagy activity via JLP in TECs. (**A** and **B**) Western blot analysis of LC3, Beclin-1, and p62 in HK-2 cells transfected with *LEF1* siRNA or control siRNA (**A**), or with pcDNA3.1 (oe-Ctrl) or LEF1-pcDNA3.1 (oe-*LEF1*) plasmid (**B**), followed by TGF-β1 stimulation. (**C**) Schematic of the mRFP-GFP-LC3 tandem probe to monitor autophagic flux. Yellow puncta indicate autophagosomes; red-only puncta indicate autolysosomes where GFP is quenched under acidic conditions. (**D** and **E**) Fluorescence microscopy of mRFP-GFP-LC3 puncta in HK-2 cells transfected with control siRNA (si-Ctrl) or *LEF1* siRNA (si-*LEF1*) (**D**), or pcDNA3.1 (oe-Ctrl) or LEF1-pcDNA3.1 (oe-*LEF1*) plasmid (**E**), after TGF-β1 treatment. Right panel: Quantitative data for mRFP^+^GFP^–^ or yellow (mRFP^+^GFP^+^) LC3 puncta per cell (*n* = 3 independent experiments). Scale bars: 50 μm and 5 μm (insets). (**F**) Western blotting showing the relative protein levels of fibronectin and collagen I in HK-2 cells transfected with either *LEF1* siRNA or control siRNA, following rapamycin (100 nM, 24 hours) and TGF-β1 stimulation. (**G**) Western blotting showing the relative protein levels of fibronectin and collagen I in HK-2 cells transfected with either oe-Ctrl or oe-*LEF1* plasmid, following CQ (20 μM, 24 hours) and TGF-β1 stimulation. (**H**–**K**) Western blotting and quantification of fibronectin, collagen I, LC3, Beclin-1, and p62 in HK-2 cells with different treatments (*n* = 3 independent experiments). Statistical analysis was performed using 1-way ANOVA followed by Tukey’s multiple-comparison test (**D**, **E**, **I**, and **K**). Data are mean ± SD.

JLP has been reported to play a role in autophagy activation (15), a process closely linked to the progression of renal fibrosis (28–31). In HK-2 cells, JLP knockdown enhanced TGF-β1–induced autophagy, as evidenced by increased LC3-II and Beclin-1 levels and a marked reduction in the autophagy substrate p62 (Supplemental Figure 5A). Conversely, overexpression of JLP suppressed TGF-β1–induced autophagy activation (Supplemental Figure 5B), supporting its negative regulatory role in autophagy. To further explore the relationship between LEF1 and JLP in TEC injury, we evaluated the role of LEF1 in autophagy regulation. Upon TGF-β1 stimulation, HK-2 cells exhibited increased LC3-II and Beclin-1 levels, along with decreased p62 expression, indicating autophagy activation (Figure 4A). LEF1 knockdown markedly reversed these changes, suggesting that LEF1 contributes to TGF-β1–induced autophagy in TECs (Figure 4A and Supplemental Figure 6A). In contrast, LEF1 overexpression further enhanced LC3 and Beclin-1 expression and reduced p62 levels (Figure 4B and Supplemental Figure 6B).

To monitor the maturation process of autophagosomes converted into autolysosomes, which called autophagic flux, we utilized a monomeric red fluorescent protein (mRFP)–GFP tandem fluorescently tagged LC3 (tfLC3) plasmid methods. In this assay, GFP fluorescence is quenched in the acidic environment of autolysosomes, while mRFP remains stable, allowing for discrimination between autophagosomes (mRFP^+^GFP^+^) and autolysosomes (mRFP^+^GFP^–^) (Figure 4C) (12, 31). As shown in Figure 4D, mRFP^+^GFP^+^ (yellow) LC3 puncta were observed under basal conditions, indicating a basal autophagy activity. TGF-β1 stimulation observably increased mRFP^+^GFP^–^ LC3 puncta, while this effect was attenuated by LEF1 silencing in HK-2 cells (Figure 4D). In contrast, LEF1 overexpression increased mRFP^+^GFP^–^ puncta in response to TGF-β1 (Figure 4E), confirming its role in promoting autophagic flux.

To further investigate the role of LEF1 in autophagy susceptibility, we treated HK-2 cells with pharmacological autophagy modulators. Rapamycin, an autophagy activator, aggravated TGF-β1–induced fibrotic makers expression (fibronectin, collagen I), which was substantially mitigated by LEF1 silencing (Figure 4F and Supplemental Figure 6C). Conversely, treatment with the autophagy inhibitor chloroquine (CQ) reduced fibrotic marker expression, but its protective effects were partial diminished in LEF1-overexpressing HK-2 cells (Figure 4G and Supplemental Figure 6D). To determine whether the profibrotic effects of LEF1-mediated autophagy are dependent on JLP, we manipulated JLP expression in LEF1-knockdown HK-2 cells. LEF1 silencing markedly suppressed TGF-β1–induced autophagy and fibrotic marker expression, effects that were exacerbated by JLP knockdown (Figure 4, H and I) and reversed by JLP overexpression (Figure 4, J and K). These results collectively suggest that the LEF1/JLP axis drives renal fibrosis, at least in part, through autophagy enhancement.

### TEC-specific Lef1 knockout attenuates renal fibrosis.

TEC-specific *Lef1*-deleted (*Lef1*^cKO^) mice were generated by crossing *Ksp-Cre* mice with *Lef1^fl/fl^* mice (Supplemental Figure 7, A and B). The knockout efficiency was confirmed through quantitative PCR (qPCR) (Supplemental Figure 7C) and Western blotting (Supplemental Figure 7D), showing a remarkable reduction in LEF1 mRNA and protein levels in the kidney of *Lef1*^cKO^ mice compared with *Lef1^fl/fl^* mice. Immunohistochemistry (IHC) further confirmed the successful ablation of the LEF1 in *Lef1*^cKO^ mice (Supplemental Figure 7E). In addition, we assessed the kidney weight/body weight ratio (Supplemental Figure 7F), urine protein-to-creatinine ratio (UPCR) (Supplemental Figure 7G), serum creatinine, serum urea, eGFR (Supplemental Figure 7H), and renal histology (Supplemental Figure 7I), in wild-type, *Lef1^fl/fl^*, and *Lef1^cKO^* mice. No significant differences were observed among these groups under basal conditions, indicating that *Lef1* deletion does not alter normal kidney morphology or function.

**Figure 5:**
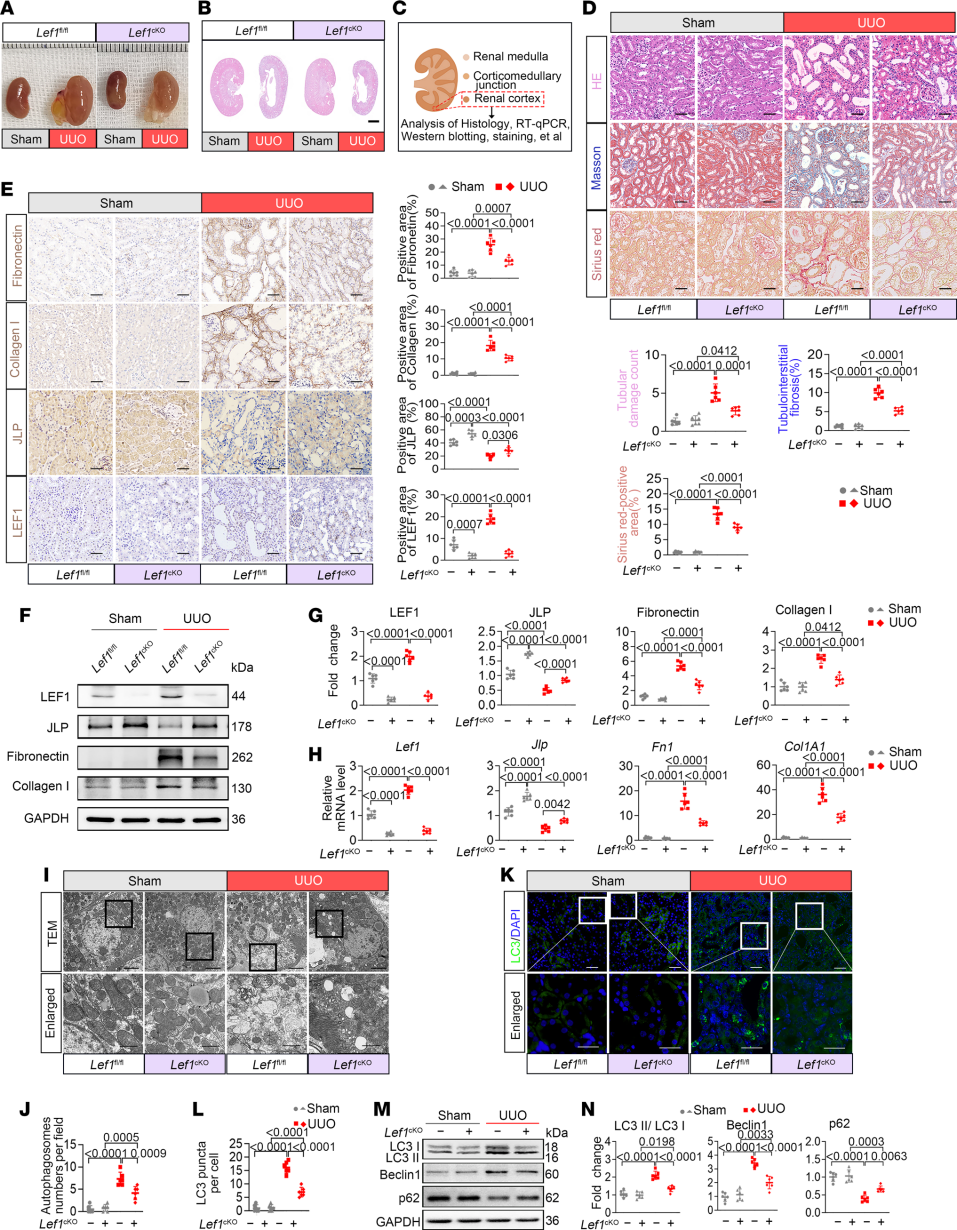
Renal tubule–specific *Lef1* deficiency ameliorates renal fibrosis. (**A**) Gross appearance of kidneys from the indicated groups. (**B**) Photomicrographs exhibiting the H&E staining of kidney sections from the indicated groups. Scale bar: 2 mm. (**C**) Schematic diagram indicating the region of the kidney (renal cortex, highlighted area) used for histological and molecular analyses. (**D**) H&E, Masson’s trichrome, and Sirius red staining of kidney tissues from the indicated group. Tubular damage score was quantified from staining; the percentage of tubulointerstitial fibrosis was quantified from Masson’s trichrome– or Sirius red–stained kidney sections using ImageJ (*n* = 6 mice per group). Scale bars: 50 μm. (**E**) Immunohistochemical staining of fibronectin, collagen I, JLP, and LEF1 in kidney tissues with quantitative analysis (*n* = 6 mice per group). Scale bars: 50 μm. (**F** and **G**) Western blot analysis and densitometric analysis of LEF1, JLP, fibronectin, and collagen I normalized to GAPDH (*n* = 6 mice per group). (**H**) Relative mRNA levels in kidney tissues in the 4 groups were calculated by normalization to GAPDH mRNA (*n* = 6 mice per group). (**I**) Representative TEM images of renal tubular cells from indicated groups. Scale bars: 2 μm and 1 μm (enlarged insets). (**J**) Quantification of results in **I**. The number of autophagosomes was counted per field. Statistical analyses were performed on data from 5 independent experiments, with counts of more than 30 fields (*n* = 6 mice per group). (**K** and **L**) Immunofluorescence of LC3 (green) and DAPI (blue) in kidney sections from indicated groups. **L** shows quantification of results in **K** (*n* = 6 mice per group). Scale bars: 50 μm and 20 μm (enlarged insets). (**M** and **N**) Immunoblot analysis of LC3, Beclin-1, and p62 in sham or UUO mouse kidneys from *Lef1*^cKO^ and *Lef1^fl/fl^* littermate mice (*n* = 6 mice per group). Statistical analysis was performed using 1-way ANOVA followed by Tukey’s multiple-comparison test (**D**, **E**, **G**, **H**, **J**, **L**, and **N**). Data are mean ± SD.

*Lef1*^cKO^ and *Lef1^fl/fl^* littermate mice that underwent UUO (hereafter referred to as *Lef1*^cKO^-UUO mice and *Lef1^fl/fl^*-UUO mice, respectively) were used to evaluate LEF1’s role in renal fibrosis. UUO challenge led to changes in renal morphology, upregulation of fibrotic markers, enhanced tubular autophagy, and loss of JLP expression in renal tissue, which is consistent with previous reports (15, 22). However, *Lef1*^cKO^-UUO mice exhibited improved renal morphology (Figure 5, A and B), fibrotic injury, tubular damage, as well as increased JLP expression, compared with their littermate *Lef1^fl/fl^*-UUO mice in renal cortex (Figure 5, C–H). Autophagic vesicles were assessed by transmission electron microscopy (TEM), which revealed a distinct increase in autophagic vacuole formation in the kidneys of UUO mice compared with sham controls (Figure 5I). *Lef1*^cKO^ mice exhibited a marked reduction in autophagic vesicles compared with *Lef1^fl/fl^* mice after UUO (Figure 5J). Consistently, immunofluorescent staining showed that UUO treatment caused more LC3-positive puncta to form in the cytoplasm of renal TECs, which were observably inhibited in *Lef1*^cKO^ mice compared with their *Lef1^fl/fl^* counterparts (Figure 5, K and L). Western blot analysis further confirmed increased levels of LC3-II and Beclin-1, along with decreased p62, in UUO kidneys; these changes were notably reversed in *Lef1*^cKO^ mice (Figure 5, M and N). Moreover, these findings were mirrored by studies based on the uIRI-induced renal fibrosis model, another progressive CKD mouse model (Supplemental Figure 8). To further assess the role of LEF1 in persistent autophagy activation, we performed time-course analyses of LC3 and SQSTM1/p62 expression in both the UUO kidneys and HK-2 cells. LC3-II progressively accumulated, whereas p62 levels declined, during UUO progression (Supplemental Figure 9, A and B), consistent with findings reported by Dong et al. (32). *Lef1* deficiency blunted LC3-II accumulation and restored p62 expression under UUO or TGF-β1 stimulation (Supplemental Figure 9, A–D).

To further clarify the role of LEF1 in autophagy regulation, we conducted experiments using CQ, an autophagy inhibitor that prevents autophagosome-lysosome fusion and blocks LC3-II degradation. CQ treatment markedly alleviated renal injury and fibrosis in *Lef1^fl/fl^*-UUO mice, and this protective effect was further enhanced in *Lef1^cKO^*-UUO mice (Supplemental Figure 10, A–D). Moreover, CQ partially reversed Beclin-1 accumulation and p62 reduction in *Lef1^fl/fl^*-UUO mice kidneys, indicating effective suppression of autophagy (Supplemental Figure 10, E and F). Collectively, these results indicate that LEF1 drives autophagy activation and promotes renal fibrosis.

### AAV9-shLef1 gene therapy mitigates renal fibrosis.

**Figure 6:**
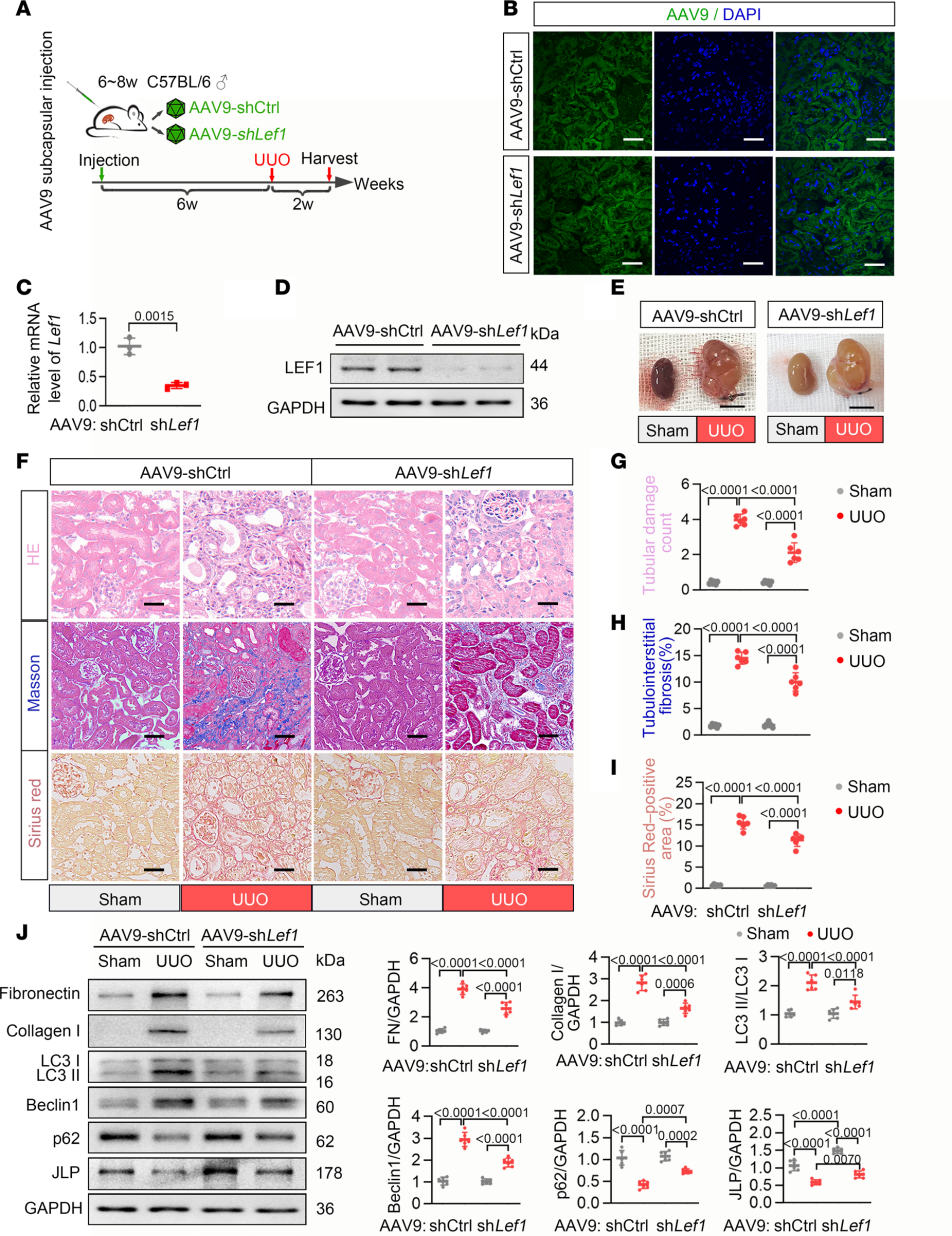
AAV9-mediated knockdown of renal *Lef1* mitigated kidney fibrosis. (**A**) Schematic of experimental design. Renal subcapsular delivery of AAV9-shCtrl or AAV9-sh*Lef1* to wild-type C57BL/6 mice at 6 weeks of age. After the delivery for 6 weeks, the mice were subjected to UUO surgery. (**B**) Fluorescence microscopic analysis of EGFP in frozen sections of mouse kidney at 1 week after injection of AAV9-shCtrl or AAV9-sh*Lef1*. Nuclei were stained with DAPI (blue). Scale bars: 50 μm. (**C** and **D**) qRT-PCR and Western blotting analysis of LEF1 expression in the whole kidney of AAV9-shCtrl or AAV9-sh*Lef1* mice (*n* = 3 mice per group). (**E**) Gross appearance of kidneys from the indicated groups. Scale bars: 5 mm. (**F**–**I**) H&E, Masson’s trichrome, and Sirius red staining of kidney tissues from the indicated group. Quantification of tubular damage score, and tubulointerstitial fibrosis percentage (*n* = 6 mice per group). Scale bars: 50 μm. (**J**) Western blot analysis and quantitative data of fibronectin, collagen I, LC3, Beclin-1, p62, and JLP of kidney tissues in the indicated groups (*n* = 6 mice per group). Statistical analysis was performed using 2-tailed Student’s *t* test (**C**) or 1-way ANOVA followed by Tukey’s multiple-comparison test (**G**–**J**). Data are mean ± SD.

The above results show that TEC-specific deletion of *Lef1* reduces renal fibrosis, highlighting the therapeutic potential of LEF1 inhibition in CKD treatment. To explore this further, we developed a gene therapy approach using adeno-associated virus serotype 9 (AAV9) for renal subcapsular administration. AAV9 carrying *Lef1*-specific short hairpin RNA (AAV9-sh*Lef1*) or control shRNA (AAV9-shCtrl) under the *Ksp-cadherin* promoter was constructed and administered via renal subcapsular injection in 6- to 8-week-old mice for 6 weeks, then challenged by UUO for 2 weeks or uIRI for 4 weeks (Figure 6A and Supplemental Figure 11A). Whole kidneys were collected to assess LEF1 depletion, revealing observably reduced mRNA and protein levels of LEF1 6 weeks after AAV9-sh*Lef1* injection (Figure 6, B–D). In UUO-induced fibrosis models, AAV9-sh*Lef1*–treated mice exhibited a marked reduction in renal fibrosis compared with AAV9-shCtrl–treated mice. This was evidenced by decreased ECM accumulation (Figure 6, E–I) and downregulation of fibrotic markers, including fibronectin and collagen I (Figure 6J). Additionally, autophagy activity was markedly suppressed in these mice, as indicated by the changes in LC3-II and Beclin-1 levels, whereas p62 and JLP expression was restored in AAV9-sh*Lef1*–treated mice (Figure 6J). Moreover, these findings were mirrored by studies based on the uIRI-induced renal fibrosis model (Supplemental Figure 11). These findings suggest that LEF1-targeted gene therapy holds promise for CKD treatment by suppressing autophagy and mitigating renal fibrotic lesions.

### Pharmacological LEF1 inhibition alleviates renal fibrosis.

**Figure 7:**
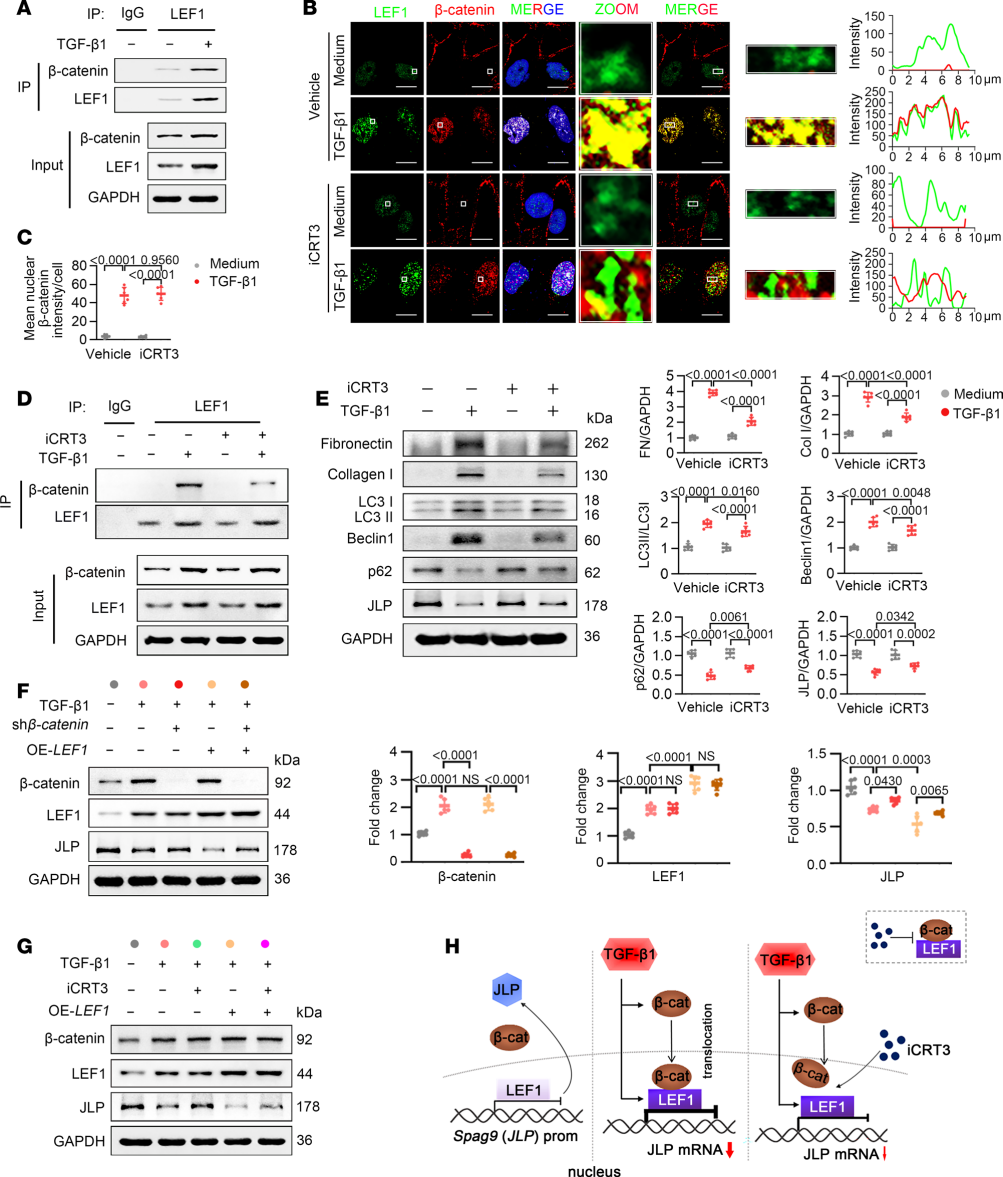
Pharmacological inhibition of LEF1 reduces TEC injury and attenuates TGF-β1–induced fibrogenic responses. (**A**) Co-IP of LEF1 and β-catenin in HK-2 cells. (**B**) Immunofluorescent (IF) staining of LEF1 and β-catenin in the indicated groups. HK-2 cells were treated with or without TGF-β1 (10 ng/mL, 24 hours) in the presence or absence of iCRT3 (10 μM) for 24 hours before IF staining. Scale bars: 20 μm. Enlarged images: original magnification ×30,000. Right panels showed the colocation along the white box in the merge images in the left panel. (**C**) Quantification of mean nuclear β-catenin IF staining intensity in HK-2 cells (*n* = 6 biologically independent samples). (**D**) Co-IP of LEF1 and β-catenin in HK-2 cells. (**E**) Western blot analysis of autophagy-related protein and fibrotic markers in HK-2 cells (*n* =6 biologically independent samples). (**F**) Western blot analysis and quantification of β-catenin, LEF1, and JLP protein levels in HK-2 cells (*n* = 6 biologically independent samples). (**G**) Western blot analysis of β-catenin, LEF1, and JLP protein levels in HK-2 cells (*n* = 6 biologically independent samples). (**H**) Working model: LEF1 negatively regulates JLP expression by binding to the promoter region of the *SPAG9* gene. Upon TGF-β1 stimulation, 2 key events occur: (i) increased LEF1 abundance further represses *SPAG9* transcription and (ii) β-catenin translocates from the plasma membrane to the nucleus, where it enhances LEF1 transcriptional activity. Pharmacological inhibition with iCRT3 disrupts the LEF1–β-catenin interaction, thereby partially restoring JLP expression and attenuating downstream fibrotic responses. Statistical analysis was performed using 1-way ANOVA with Tukey’s multiple-comparison test (**C**, **E**, and **F**). Data are mean ± SD. NS, no significant difference.

Previous studies have demonstrated that the interaction between LEF1 and β-catenin is important for LEF1-mediated transcriptional activation (33). iCRT3, a small-molecule inhibitor that specifically disrupts the LEF1–β-catenin interaction, has shown therapeutic potential in cancer (34). Our prior findings indicated that loss of LEF1 alleviated TEC injury and renal fibrosis; we further investigated the therapeutic potential of inhibiting LEF1 transcriptional activity. Co-IP assay showed TGF-β1 stimulation markedly enhanced the LEF1–β-catenin interaction (Figure 7A). Previous studies have shown that β-catenin is present both at the plasma membrane and in the nucleus. Consistently, our confocal microscopy experiments showed that majority of β-catenin was located at the plasma membrane; TGF-β1 stimulation promoted β-catenin nuclear translocation, where it colocalized with LEF1 (Figure 7, B and C). Notably, treatment with iCRT3 effectively disrupted the TGF-β1–induced LEF1–β-catenin interaction without altering LEF1 expression levels (Figure 7, B–D). In addition, iCRT3 markedly suppressed TGF-β1–induced autophagy activation and the expression of fibrotic markers in HK-2 cells (Figure 7E). Interestingly, iCRT3 treatment also partially restored JLP expression following TGF-β1 stimulation (Figure 7E), suggesting that iCRT3 disrupts the LEF1–β-catenin interaction and thereby partially inhibits LEF1 transcriptional activity. To further determine whether LEF1 activity is regulated by β-catenin, HK-2 cells were transduced with β-catenin shRNA. LEF1 overexpression exacerbated TGF-β1–induced JLP suppression, which was partially reversed by β-catenin silencing (Figure 7F and Supplemental Figure 12A). Although iCRT3 did not alter β-catenin protein levels, it similarly restored JLP expression at both the protein (Figure 7G and Supplemental Figure 12B) and mRNA (Supplemental Figure 12C) levels under TGF-β1 stimulation. These findings, in conjunction with Figure 1E, indicate that LEF1 serves as a negative regulator of JLP expression in TECs. Under basal conditions, LEF1 activity is relatively low; however, it is markedly enhanced in the presence of β-catenin and elevated levels of LEF1 itself, a condition that occurs following TGF-β1 stimulation. This implies that β-catenin functions as a cofactor, augmenting the transcriptional activity of LEF1 on JLP expression (Figure 7H).

**Figure 8:**
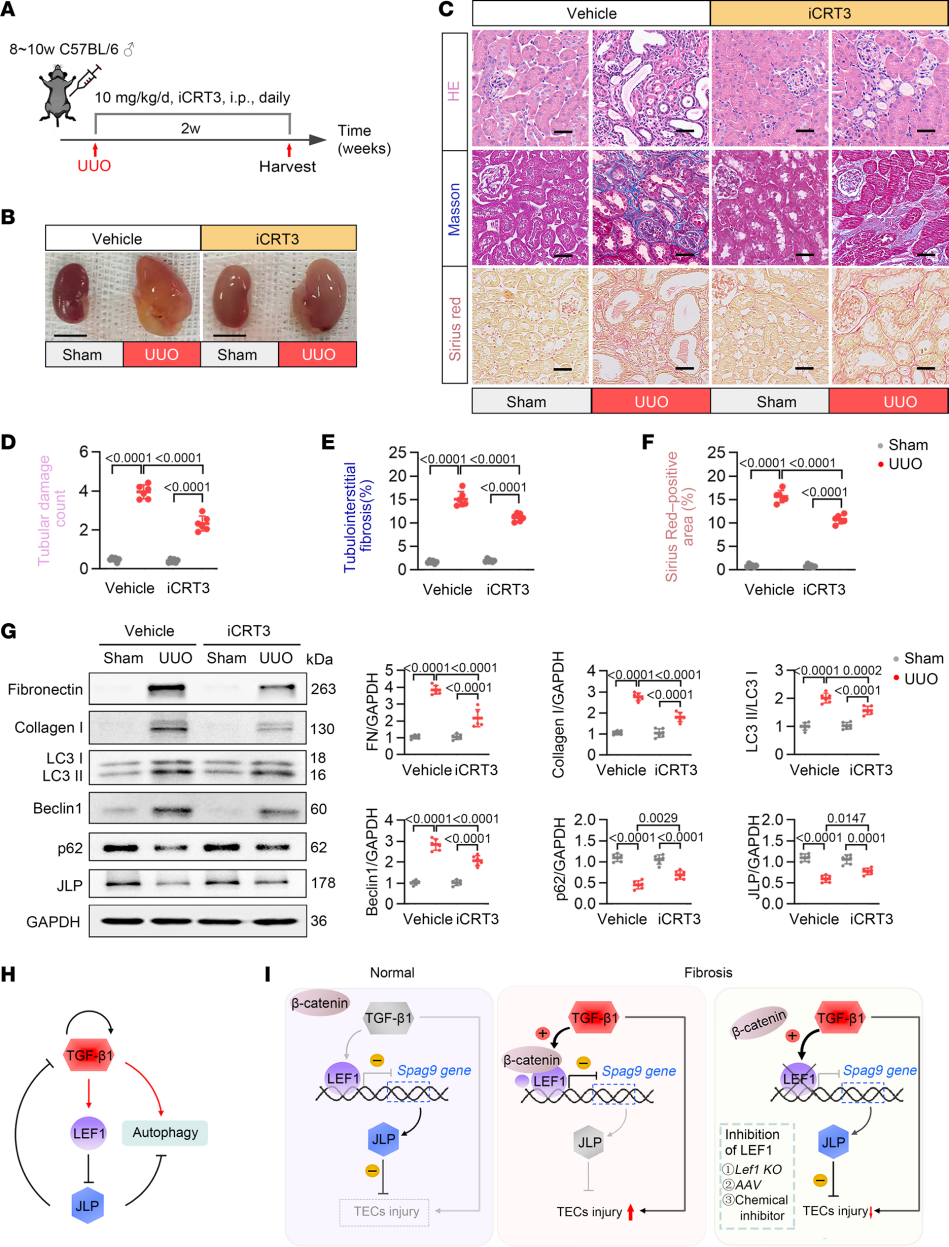
Pharmacological inhibition of LEF1 reduces TEC injury and attenuates UUO-induced renal fibrosis. (**A**) Schematic of experimental design. Wild-type C57BL/6 mice (8–10 weeks old, male) underwent UUO surgery and were administered daily i.p. injections of iCRT3 (10 mg/kg/d) for 2 weeks. (**B**) Gross appearance of kidneys from the indicated groups. Scale bars: 5 mm. (**C**) H&E, Masson’s trichrome, and Sirius red staining of kidney tissues from the indicated group. (**D**–**F**) Quantification of tubular damage score, and tubulointerstitial fibrosis percentage (*n* = 6 mice per group). Scale bars: 50 μm. (**G**) Western blot analysis and quantitative data of fibronectin, collagen I, LC3, Beclin-1, p62, and JLP of kidney tissues in the indicated groups (*n* = 6 mice per group). (**H** and **I**) Schematic illustration of the TGF-β1/LEF1/β-catenin/JLP axis in renal fibrosis. In response to TGF-β1 stimulation, the transcription factor LEF1 is specifically upregulated in tubular epithelial cells. LEF1 binds to the promoter region of the JLP gene (*SPAG9*), suppressing its transcription and expression. JLP, an intrinsic antifibrotic factor, as previously identified by our group, counteracts TGF-β1–induced fibrosis. The inhibition of JLP leads to the sustained activation of TGF-β1 signaling and persistent autophagy in TECs, which exacerbates cellular injury and accelerates the progression of renal fibrosis. TGF-β1 induces β-catenin translocation from the plasma membrane to the nucleus and interacts with LEF1, partially enhancing LEF1 transcriptional activity. The absence of LEF1, achieved through either TEC-specific knockout, AAV9-mediated gene therapy, or pharmacological inhibition of activity, effectively prevents the loss of JLP under fibrotic conditions. This preservation of JLP leads to suppression of sustained autophagy and attenuation of renal fibrosis. Statistical analysis was performed 1-way ANOVA with Tukey’s multiple-comparison test (**D**–**G**). Data are mean ± SD.

To evaluate the therapeutic effect of LEF1/β-catenin inhibition in vivo, wild-type mice subjected to UUO or uIRI were treated with daily intraperitoneal injections of iCRT3 (10 mg/kg) or PBS as control. Two weeks after UUO surgery, TEC injury and kidney fibrosis were evaluated (Figure 8A). In UUO models, treatment with iCRT3 obviously improved renal morphology (Figure 8B) and markedly attenuated tubular damage, renal fibrosis, and collagen deposition compared with vehicle-treated control mice (Figure 8, C–F). Immunoblot analysis further confirmed a substantial downregulation of fibronectin and collagen I levels in iCRT3-treated mice (Figure 8G). Additionally, iCRT3 treatment suppressed autophagy activation and restored JLP expression in UUO kidneys, aligning with its protective effects (Figure 8G). The antifibrotic efficacy of iCRT3 was further validated in the 28-day uIRI model (Supplemental Figure 13A), where similar protective outcomes were observed reinforcing its therapeutic potential (Supplemental Figure 13). Collectively, these results demonstrate that iCRT3 alleviates renal fibrosis by inhibiting LEF1 transcriptional activity and partially restoring JLP pathway function (Figure 8, H and I).

## Discussion

Our study demonstrated that LEF1 expression is markedly upregulated in TECs in response to TGF-β1 stimulation and during the progression of kidney fibrosis. Genetic or pharmacological inhibition of LEF1 expression or activity effectively mitigated fibrotic responses. Mechanistically, LEF1 directly binds to the *SPAG9* promoter, and TGF-β1 stimulation not only enhances LEF1 expression but also promotes β-catenin nuclear translocation, collectively augmenting LEF1 transcriptional activity. This enhanced activity suppresses the expression of the antifibrotic mediator JLP, thereby sustaining autophagy activation and exacerbating CKD progression. Together, these findings identified the LEF1 as a potential mediator of TEC injury and renal fibrosis through dysregulated autophagy (Figure 8, H and I; schematic illustrations).

Autophagy serves as a cellular homeostasis mechanism in response to unfavorable conditions by degrading cytoplasmic components (35). While this process primarily protects cells, uncontrolled autophagy can lead to cell death (35, 36). Basal autophagy in proximal tubular cells helps to maintain cellular integrity (28). In nephrotoxic and ischemic kidney injury models, induced autophagy in proximal tubules provides renal protection (13, 37, 38). However, persistent or overactivated autophagy can promote renal fibrosis by triggering tubular atrophy, interstitial inflammation, and production of the profibrotic factor FGF-2 (32, 39, 40). TGF-β1 has been identified as an inducer of autophagy in renal tubules both in vitro and in vivo models of kidney injury (15, 32). Our findings support these studies; *LEF1*^cKO^ mice displayed reduced autophagy and attenuated fibrosis, while in vitro experiments confirmed that LEF1 knockdown suppressed TGF-β1–induced autophagy, at least in part through the restoration of JLP expression, whereas LEF1 overexpression further enhanced autophagic activity. These results underscore the pathogenic role of persistent autophagy activation in TECs during CKD progression and highlight the importance of LEF1 in sustaining maladaptive autophagic activity. However, the precise mechanism by which the LEF1/JLP axis regulates autophagy remains unclear. Previous studies have shown that lysosomal positioning is a key determinant of autophagic activity and other cellular processes (41). JLP (also known as JIP4) is a scaffold protein that interacts with both kinesin-1 and the dynein-dynactin complex to regulate retrograde lysosomal transport (42). Notably, JLP-mediated lysosome repositioning toward the microtubule-organizing center has been implicated in autophagy activation in neurons (43, 44). Whether LEF1 regulates autophagy through JLP-dependent lysosomal trafficking remains to be elucidated.

LEF1 is a key transcriptional effector of both the Wnt/β-catenin and TGF-β1 signaling pathways, regulating diverse pathological processes, including tumorigenesis and tissue remodeling (26, 45). Although aberrant Wnt signaling has long been implicated in renal fibrosis (46, 47), the specific contribution of LEF1 to CKD has remained unclear. A previous study reported that elevated LEF1 mRNA levels in human diabetic kidney disease (DKD) glomeruli by a microarray analysis (48). Igarashi et al. demonstrated that ablation of HNF-1β in mIMCD3 renal epithelial cells leads to increased LEF1 expression (49, 50), and that elevated LEF1 expression and nuclear localization are observed in cystic kidneys from *Hnf1b*-mutant mice. In this study, we demonstrate that LEF1 expression is markedly upregulated in TECs following TGF-β1 stimulation and during kidney fibrosis progression. Functionally, silencing LEF1 or inhibiting its activity attenuated renal fibrosis by reducing excessive autophagy, indicating that LEF1 serves as a profibrotic regulator in CKD.

Mechanistically, we found that LEF1 acts as a transcriptional repressor of JLP (encoded by *SPAG9*), a multifunctional scaffolding protein that coordinates intracellular signaling and vesicle trafficking (21, 51–54). Previous studies have shown that aberrant expression of JLP disrupts its role in maintaining cellular homeostasis and contributes to the pathogenesis of human diseases (20, 55–58). JLP overexpression has been observed in numerous tumor cells and immortalized cell lines, where it promotes proliferation, migration, and invasion (18, 57–62). In contrast to neoplastic conditions, fibrotic tissues in the kidneys and peritoneum exhibit JLP downregulation (15, 23). JLP in TECs functions as an endogenous antifibrotic molecule by counteracting TGF-β1–induced ECM production, EMT, apoptosis, cell cycle arrest, and dysregulated autophagy (15). Our data reveal that LEF1 directly binds to the *SPAG9* promoter and suppresses its transcription. Both bioinformatic analysis and ChIP assays identified multiple LEF1-binding motifs within the *SPAG9* promoter region, and TGF-β1 stimulation enhanced this LEF1-DNA interaction. As a result, elevated LEF1 expression leads to transcriptional repression of JLP, loss of its autophagy-regulating function, and subsequent overactivation of autophagic flux in TECs. Previous studies have reported that LEF1 binds to the proximal promoter region of *SPAG9*, located within 360 bp upstream of the transcription start site, thereby acting as a transcriptional activator to enhance *SPAG9* expression in KSHV-associated tumors (27). Li et al. identified 2 LEF1-binding motifs, CTTTGTGA and GGTCAAAG, within the *SPAG9* promoter region (27). In the present study, we further confirmed LEF1 occupancy at 3 binding motifs within the *SPAG9* locus in HK-2 cells. Comparative analysis of LEF1-binding patterns between tumor cells and renal TECs revealed that the CTTTGTGA motif is conserved across both contexts, suggesting that LEF1 may modulate *SPAG9* expression through context-dependent regulatory mechanisms.

Importantly, the LEF1/JLP regulatory relationship appears to be context dependent. We propose that JLP expression may co-regulated by LEF1, acting as a negative transcriptional regulator, and by yet-unidentified positive transcription factor(s). Under physiological conditions, LEF1 expression and nuclear localization are minimal, allowing positive regulators to maintain JLP expression at a basal level sufficient to support normal autophagic homeostasis. Upon pathological stimulation — such as TGF-β1 exposure or renal injury — LEF1 expression increases, and β-catenin translocates into the nucleus to form a transcriptionally active LEF1–β-catenin complex. This complex strengthens LEF1 binding on the *SPAG9* promoter, amplifying transcriptional repression of JLP. Thus, the LEF1/JLP axis remains largely quiescent under homeostatic conditions but becomes strongly engaged during disease, serving as a key regulatory switch that links TGF-β1 and Wnt signaling to sustained autophagy activation and fibrogenesis in CKD.

Interestingly, members of the TCF/LEF family, including LEF1, can function as either transcriptional activators or repressors, depending on their cofactors and cellular environment (63). This dual functionality underscores the complexity of LEF1’s role in different diseases, including renal fibrosis, highlighting the need for further exploration of its regulatory mechanisms. In tumors, LEF1 promotes JLP expression, in contrast to its repressive role in TECs. This dual functionality may arise from variations in the recruitment of co-activators or co-repressors as well as intrinsic histone deacetylase activity differences (64). The diversity in LEF1 effects could also stem from distinct cotranscription factors (β-catenin, et al.) interacting with LEF1 across various disease contexts (65). Notably, our study demonstrated that iCRT3 exerts antifibrotic effects by inhibiting the interaction between LEF1 and β-catenin, thereby suppressing LEF1 transcriptional activity and restoring JLP expression in TECs. Therefore, further investigation of these molecular mechanisms is warranted to elucidate LEF1’s context-dependent effects. Our data highlight the pivotal role of LEF1 in renal fibrogenesis and suggest that LEF1-targeted interventions may improve CKD outcomes. Recent studies have demonstrated the potential of LEF1 inhibitors, such as 3PO, in targeting cancer cells, offering promise for CKD treatment. Future clinical investigations are necessary to assess the therapeutic potential of these inhibitors in suppressing CKD progression.

There are some limitations of our study. Clinical trials targeting TGF-β1 signaling in CKD have yielded disappointing results (66, 67), underscoring the complexity of fibrotic signaling networks in human disease. Indeed, LEF1 expression in CKD may be regulated by multiple upstream factors beyond TGF-β1. A previous study found that loss of the adapter protein CD2-associated protein (CD2AP), which is essential for maintaining glomerular integrity, leads to upregulation of *Lef1* and *Tcf1* mRNAs in podocytes and exacerbates kidney injury (68). In addition, microarray analyses have revealed increased *LEF1* mRNA levels in glomeruli from patients with DKD (48). Furthermore, Igarashi et al. demonstrated that ablation of *HNF-1β* in renal epithelial cells increases LEF1 expression (49, 50). Together, these findings suggest that LEF1 upregulation in human CKD may occur through diverse mechanisms, not limited to TGF-β1 signaling.

The limited success of direct TGF-β1 inhibition suggests that we need to look beyond TGF-β1 itself and focus on downstream or modulatory components that more specifically drive fibrosis. Previous studies have shown that targeting TGF-β1 modulators such as BAMBI or LRG1 can selectively suppress profibrotic signaling without interfering with TGF-β1’s essential physiological functions (69–71). In line with this concept, our findings indicate that LEF1 acts downstream of TGF-β1 to promote autophagy activation and fibrosis, pointing to the LEF1/JLP axis as a promising therapeutic target. Future work using human kidney organoids or patient-derived samples will help confirm this mechanism and its relevance to human CKD.

In summary, our study reveals an important role of the TGF-β1/LEF1/β-catenin/JLP axis in renal fibrosis. We demonstrate that LEF1 drives the persistent activation of autophagy in TECs, contributing to the progression of kidney fibrosis. Mechanistically, the scaffolding protein JLP acts as negative regulator of autophagy in TECs, while the transcription factor LEF1 serves as repressor for JLP expression. Under normal condition, autophagy is maintained at basal levels in the presence of JLP due to weak LEF1 expression. However, in the context of progressive CKD, intensive TGF-β1 signaling led to nuclear accumulation of LEF1 that binds to the promoter of JLP gene (*SPAG9*), resulting in the loss of JLP and persistent autophagy activity in TECs, which ultimately facilitates renal fibrosis. These findings provide mechanistic insights into the regulation of TEC autophagy and kidney fibrosis via the TGF-β1/LEF1/β-catenin/JLP axis.

## Methods

### Sex as a biological variable.

In our human studies, we examined men and women, and similar findings were reported for both sexes. In contrast, our animal studies used only male mice to avoid potential interference from sex hormones.

### Human kidney biopsy specimens.

Obstructive kidneys were obtained from patients with obstructive nephropathy, paratumor tissue from patients with renal carcinoma, and human renal biopsy specimens from patients with CKD. The clinical demographics of these patients are provided in Supplemental Tables 1 and 2.

### Mice and animal models.

Specific pathogen–free (SPF) C57BL/6 wild-type mice and *Lef1^fl/fl^* (catalog S-CKO-03376) mice were purchased from Cyagen Biosciences Suzhou Inc. and maintained at the Center for Animal Experiments at Wuhan University. Conditional knockout mice were generated using the Cre/loxP system. To create renal tubule–specific *Lef1*-knockout mice (*Lef1^fl/fl^;Cre^+^*, hereafter referred to as *Lef1*^cKO^), *Lef1^fl/fl^* mice on a C57BL/6 background were crossed with *Ksp-Cre* transgenic mice (Cyagen, C001022). *Lef1^fl/fl^;Cre^-^* littermates (referred to as *Lef1^fl/fl^*) served as controls. All experimental mice were backcrossed to C57BL/6 for at least 10 generations as confirmed by the vendor, and were male and matched by age and body weight (8–10 weeks old, 20–25 g). Transgenic mice were identified by standard PCR genotyping. *Ksp-Cre* was genotyped using primers 5′-GCAGATCTGGCTCTCCAAAG-3′ and 5′-AGGCAAATTTTGGTGTACGG-3′. *Lef1* was genotyped using the primers 5′-GTGCGATTTTGAAATGTGATGCC-3′ and 5′-GTAGCTTTTCAAAGTGGCGTTCT-3′.

A UUO mouse model was established as previously described (6). SPF C57BL/6 mice male (8–10 weeks old) underwent ligation of the left ureter with 4-0 silk suture at 2 locations and cutting to prevent urinary tract infection. The UUO control mice underwent sham surgery of the right ureter. The mice were sacrificed, and the kidneys were harvested 14 days after UUO. The uIRI mouse model for progressive kidney fibrosis was induced in male C57BL/6 as described below, and the left renal artery was clamped with a microvascular clamp for 30 minutes at 37°C using a heating device, followed by reperfusion. A sham operation was performed on the right kidney instead of its removal. Mice were sacrificed 28 days after modeling (6).

### Administration of iCRT3 in vivo.

Based on previous studies, mice were administered iCRT3 (TargetMol, T4302; CAS 901751-47) via intraperitoneal injection at a dose of 10 mg/kg/d (28). SPF C57BL/6 mice (male, matched by age and body weight, 8–10 weeks old) underwent UUO or uIRI surgery, followed by daily intraperitoneal injections of iCRT3 or PBS (control), starting on the day of surgery and continuing for a specified duration.

### Administration of CQ in vivo.

Mice received CQ (TargetMol, T8689; CAS 54-05-7) at a dose of 30 mg/kg by intraperitoneal injection starting on the first day after establishment of the UUO or uIRI model. Thereafter, CQ was administered intraperitoneally 3 times per week until sample collection.

### AAV-infected mice.

C57BL/6 mice (male, aged 6–8 weeks old, 20–25 g) were used in these experiments. AAV9 encoding mouse LEF1 was obtained from Huameng Biotechnology. Mice were anesthetized with an intraperitoneal injection of pentobarbital sodium (30 mg/kg) and injected with 1.0 × 10^12^ vector genome copies of AAV9 encoding an shRNA targeting Lef1 (sh*Lef1*) or a control shRNA (sh-Ctrl). The injections were administered at 5 different sites in the renal cortex. Six weeks after AAV administration, mice were subjected to UUO or uIRI surgery.

### Cell culture and treatment.

Human renal TECs (HK-2) were originally obtained from ATCC (catalog CBP60447), subsequently maintained in our laboratory, and cultured in DMEM/F12 medium (HyClone, SH30023.01) supplemented with 10% fetal bovine serum (FBS; HyClone, SV30208.02) and 1% penicillin-streptomycin (Beyotime Biotechnology, ST488S) at 37°C in 5% CO_2_. HK-2 cells were synchronized with DMEM/F12 medium without FBS for 12 hours and treated with 10 ng/mL TGF-β1 (MedChemExpress, HY-P78168) for 24 hours.

For siRNA transfection, control siRNA and *LEF1* siRNA (Sangon Biotech) were transfected into HK-2 cells using Lipofectamine RNAiMAX (Thermo Fisher Scientific, 13778150) following the manufacturer’s protocol. siRNAs (si-Ctrl and si-*LEF1*) were obtained from Sangon Biotech.

For plasmid transfection, pcDNA3.1, LEF1-pcDNA3.1 (Miaoling Biology, P27315) and mRFP-GFP-LC3 (Miaoling Biology, P48062) plasmids were constructed using the MiaoLing Plasmid Platform and transfected into HK-2 cells using Lipofectamine 3000 (Thermo Fisher Scientific, L3000015), according to the manufacturer’s instructions.

### Antibodies.

The following antibodies were used: rabbit anti-LEF1 (Abcam, ab137872), rabbit anti-LEF1 (Cell Signaling Technology, D6J2W), rabbit anti-fibronectin (Abcam, ab2413), rabbit anti–collagen I (Proteintech, 14695-1-AP), rabbit anti-JLP (Abcam, ab12331), mouse anti-JLP (Santa Cruz Biotechnology, sc-271492), rabbit anti-F4/80 (Abcam, ab300421), mouse anti–α-SMA (Abcam, ab7817), rabbit anti-p62/SQSTM1 (Servicebio, GB11531), rabbit anti–Beclin-1 (ABclonal, A21191), rabbit anti-LC3B (Servicebio, GB113801), rabbit anti-LC3B (Abcam, ab192890), rabbit anti–β-catenin (Abcam, ab32572), mouse anti-GAPDH (Proteintech, 60004-1-Ig), HRP–goat anti-mouse IgG (Antgene, ANT019), HRP–goat anti-rabbit IgG (Antgene, ANT020), LTL (Vector, FL-1321), and DBA (Vector, FL-1031).

### Dual-luciferase reporter assay.

The transcriptional activity of the *SPAG9* promoter was assessed using dual-luciferase reporter assays (GeneCreate). DNA fragments of the *SPAG9* promoter were cloned into the pGL3-Basic vector. HEK293T cells were cotransfected with this reporter construct and the pRL-TK *Renilla* luciferase plasmid as an internal control. Cells were cotransfected with a *LEF1*-expressing plasmid (LEF1-pcDNA3.1) and treated with TGF-β1 for 24 hours. Luciferase activity was measured 24–48 hours after transfection, and firefly luminescence was normalized to *Renilla* luminescence. Data are from 5 independent experiments performed in triplicate.

### Western blotting.

Kidney tissues and cultured cells were lysed in RIPA lysis buffer (Beyotime, P0013B) supplemented with 1% protease and phosphatase inhibitor cocktails (Beyotime, P1045) on ice for 30 minutes. Lysates were centrifuged at 12,000*g* for 15 minutes at 4°C, and the supernatants were collected. Protein concentrations were determined using the Pierce BCA Protein Assay Kit (Beyotime, P0012) according to the manufacturer’s instructions. The samples were separated by SDS-PAGE and transferred to PVDF membranes (Merck Millipore, IPVH00010). After blocking in TBST (20 mM Tris-HCl, 150 mM NaCl, 0.1% Tween 20) with 5% skim milk, membranes were incubated with primary antibodies overnight at 4°C. Following 3 washes with TBST, membranes were incubated with HRP-conjugated secondary antibodies for 1 hour at room temperature. Membranes were imaged with the ChemiDoc MP System and analyzed using Image Lab 3.0 software (Bio-Rad Laboratories).

### Quantitative real-time PCR.

Total RNA from kidney tissues and cells was extracted using RNAiso Plus (TaKaRa, 9109) according to the manufacturer’s protocol. RNA concentration and purity were determined spectrophotometrically using a NanoDrop 2000 (Thermo Fisher Scientific). For reverse transcription, 1 μg of total RNA was converted into complementary DNA (cDNA) using the ABScript III RT Master Mix for qPCR with a gDNA Remover kit (ABclonal, RK20429), which includes a genomic DNA elimination step to prevent contamination. Quantitative real-time PCR (qRT-PCR) was carried out using SYBR Green Fast qPCR Mix (ABclonal, RK21203) on a CFX-96 Real-time PCR system (Bio-Rad Laboratories). Each 20 μL reaction contained 10 μL of SYBR Green mix, 1 μL each of forward and reverse primers, 2 μL of diluted cDNA template, and nuclease-free water. The 2^−ΔΔCt^ method was used to calculate mRNA levels, and *GAPDH* was used to standardize the gene expression measurements. The sequences of the primer pairs are shown in Supplemental Table 3.

### Histology and IHC.

Kidney tissues were fixed in 4% paraformaldehyde (pH 7.4) and embedded in paraffin. Following deparaffinization in xylene and rehydration through a descending ethanol series, sections were prepared for staining, paraffin-embedded kidney sections (4 μm) were stained with H&E (Servicebio, G1005), Masson’s trichrome (Servicebio, G1006), and Sirius red (Servicebio, G1078), following the manufacturer’s protocols to assess morphological changes and collagen deposition.

For IHC, paraffin-embedded kidney sections (4 μm) were deparaffinized, hydrated, antigen retrieved, and blocked, followed by incubation with the corresponding primary antibodies at 4°C overnight, followed by incubation with corresponding secondary antibodies at room temperature for 1 hour. DAB (Servicebio, G1212) staining, hematoxylin staining, dehydration, and sealing were performed.

Kidney sections were examined under a microscope (BX53, Olympus). Image analysis was performed with Image J v1.37c analysis software (NIH). The percentage of tubulointerstitial fibrosis was quantified from Masson’s trichrome– or Sirius red–stained kidney sections using ImageJ software. For each section, the positively stained area was measured and expressed as a percentage of the total interstitial area.

### Immunofluorescent staining.

Paraffin sections were used for tissue immunofluorescence, following the same protocol as that used for IHC, until primary antibody incubation. HK-2 cells were fixed with 4% paraformaldehyde for 30 minutes at room temperature, followed by a 1-hour incubation with 5% bovine albumin V blocking and primary antibody incubation at 4°C overnight. The sections were then incubated with secondary antibodies at 37°C for 1 hour in the dark and counterstained with DAPI (Antgene, ANT063) before staining. LTL and DBA were used to identify proximal and distal tubules in mice. The sections were examined under a microscope (FV1200, Olympus). Five random visual fields were selected, and image analysis was performed using the ImageJ software. “Expression intensity” refers to the total integrated optical density of LEF1 immunofluorescence signals measured per field. “Mean expression intensity” refers to the average signal intensity per positive cell (total integrated optical density divided by the number of LEF1-positive cells).

### TEM.

Cortical kidney tissues were fixed with glutaraldehyde (2.5%) at 4°C overnight. The tissues were sectioned into ultrathin (40 nm) slices and stained with uranyl acetate. A Hitachi transmission electron microscope was used to analyze the sections. All TEM procedures, including sectioning, staining, and imaging, were commissioned to Servicebio Technology Co., Ltd., with quality control ensured by standardized operational protocols.

### ChIP analysis.

ChIP assays were performed using the Pierce Magnetic ChIP Kit (Thermo Fisher Scientific, 26157) following the manufacturer’s instructions. Briefly, cells were crosslinked with 1% formaldehyde for 10 minutes at room temperature to preserve protein-DNA interactions, and the reaction was quenched by adding 125 mM glycine for 5 minutes. Cells were then harvested and resuspended in membrane extraction buffer supplemented with protease and phosphatase inhibitor cocktails. Nuclei were pelleted by centrifugation and resuspended in micrococcal nuclease digestion buffer working solution, followed by micrococcal nuclease digestion at 37°C for 15 minutes to partially fragment chromatin. Residual large fragments were further sheared by brief sonication to obtain chromatin fragments of approximately 200–500 bp. For immunoprecipitation, chromatin extracts were incubated overnight at 4°C with anti-LEF1 antibody (Cell Signaling Technology, D6J2W) or normal rabbit IgG as a negative control. Immune complexes were captured using ChIP-grade Protein A/G magnetic beads, washed to reduce nonspecific binding, and eluted from the beads. Crosslinks were reversed by incubation at 65°C for 1 hour, and proteins were digested with proteinase K. The purified DNA was recovered using the spin column purification system provided in the kit and analyzed by qRT-PCR using specific primer pairs listed in Supplemental Table 4.

### Statistics.

Data were processed using GraphPad Prism 8.0 and are presented as mean ± SD. A 2-tailed, unpaired Student’s *t* test or 1-way analysis of variance (ANOVA) with Tukey’s multiple-comparison test was used to compare differences between groups. Statistical significance was set at a *P* value of less than 0.05.

### Study approval.

The human study was approved by the Clinical Research Ethics Committee of the Renmin Hospital of Wuhan University, with informed patient consent (Approval No. WDRY2023-K095). All animal care and experimental procedures complied with the guidelines of the Animal Care and Use Committee of the Renmin Hospital of Wuhan University (Approval No. 20210703).

### Data availability.

All data generated or analyzed in this study were included in the main text and the supplemental material for this article. Values for all data points in graphs are reported in the Supporting Data Values file.

## Author contributions

CL, MZ, and MT co-authored a first draft of the manuscript. The order of co–first authors CL, MZ, and MT, was determined by their contribution to the study. LZ and HW designed the research. CL and MZ performed the major experiments. CL contributed to the cellular experiments. CL, MZ, and MT contributed to the animal experiments. CL, MZ, ZT, YH, YL, XW, JZ, YW, and LQ contributed to the data analysis. LZ and MZ wrote the manuscript. LZ, JW, XC, CC, and HW revised the manuscript. LZ, XL, and HW supervised the entire study. All authors read and approved of the final manuscript.

## Conflict of interest

The authors have declared that no conflict of interest exists.

## Funding support

- National Natural Science Fund of China (nos. 82270711, 82370682, and 81800614).
- Key-Area Research and Development Program of Hubei Province (no. 2025BCB017).
- Hubei Province Major Scientific and Technological Special Project (no. 2019ACA137).

## Supplementary Material

Supplemental data

Unedited blot and gel images

Supporting data values

## Version 1 01/08/2026

In-Press Preview

## Version 2 07/08/2026

Electronic publication
