# Supplementary material for: A TGF-β1/LEF1/β-catenin/JLP network motif regulates autophagy and tubule injury in renal fibrosis
Source: JCI Insight. 2026 Jan 8;11(13):e196835. doi: 10.1172/jci.insight.196835 (PMC13460821; doi:10.1172/jci.insight.196835)
Supplement: Supplementary file 1 [file jciinsight-11-196835-s160.pdf]

SUPPLEMENTARY MATERIALS

A TGF-β1/LEF1/β-catenin/JLP network motif regulates autophagy and tubule injury in renal fibrosis

Chen Li[1,#], Meng Zhang[1,#], Maoqing Tian[1,#], Zeyu Tang[1], Yuying Hu[1], Yuyu Long[1], Xiaofei Wang[1], Liwen Qiao[1], Jiefei Zeng[1], Yujuan Wang[1,2], Xinghua Chen[1,2], Cheng Chen[1,2], Xiaoyan Li[3], Lu Zhang[1,2,*], Huiming Wang[1,2,^,*]

[1]Department of Nephrology, Renmin Hospital of Wuhan University, Wuhan, 430060, China
[2]Hubei Provincial Clinical Research Center for Kidney Disease, Wuhan, 430060, China
[3]Department of Internal Medicine, Mayo Clinic, Rochester, Minnesota, USA; Department of Biochemistry and Molecular Biology, Mayo Clinic, Rochester, Minnesota, USA.
^ Present affiliation: Department of Nephrology, Zhongnan Hospital of Wuhan University, Wuhan, 430060, China
[#] These authors contributed equally: Chen Li, Meng Zhang, Maoqing Tian
[*] Address correspondence to: Lu Zhang, Department of Nephrology, Renmin Hospital of Wuhan University, E-mail: rm002714@whu.edu.cn. Or to: Huiming Wang, Department of Nephrology, Zhongnan Hospital of Wuhan University, E-mail: rm000301@whu.edu.cn.

TABLE OF CONTENTS

1. Supplementary Figures and Legends.

1) Figure S1. LEF1 represses JLP gene transcription. (Related to Figure 1).
2) Figure S2. LEF1 expression is elevated during CKD progression. (Related to Figure 2).
3) Figure S3. LEF1 expression is elevated in UUO mice models accompanied by reduced expression of JLP. (Related to Figure 2).
4) Figure S4. JLP expression inversely correlates with LEF1 level in fibrotic mouse kidneys. (Related to Figure 2).
5) Figure S5. JLP mediates TECs autophagy. (Related to Figure 4).
6) Figure S6. LEF1 regulates TECs autophagy and fibrotic response. (Related to Figure 4).
7) Figure S7. Generation of TEC-specific *Lef1* knockout mouse. (Related to Figure 5).
8) Figure S8. Renal TEC-specific *Lef1* deficiency ameliorates renal fibrosis in uIRI (28d) mouse model. (Related to Figure 5).
9) Figure S9. LEF1 regulates autophagy dynamics in the UUO mouse model and in HK-2 cells following TGF-β1 stimulation. (Related to Figure 5).
10) Figure S10. Administration of Chloroquine attenuates renal fibrosis in the UUO model. (Related to Figure 5).
11) Figure S11. AAV9-*shLef1* gene therapy mitigates uIRI-induced renal fibrosis. (Related to Figure 6).
12) Figure S12. Inhibition of LEF1 reduces TECs injury under TGF-β1 treatment. (Related to Figure 7).
13) Figure S13. Pharmacological inhibition of LEF1 attenuates uIRI-induced renal fibrosis. (Related to Figure 8).

2. Supplementary Tables.

1) Table S1. Baseline characteristics of CKD and adjacent normal tissues patients.
2) Table S2. Clinical information of patients with obstructive nephropathy.
3) Table S3. Primer sequences used for RT-qPCR in this study.
4) Table S4. Primer sequences used for ChIP-qPCR in this study.

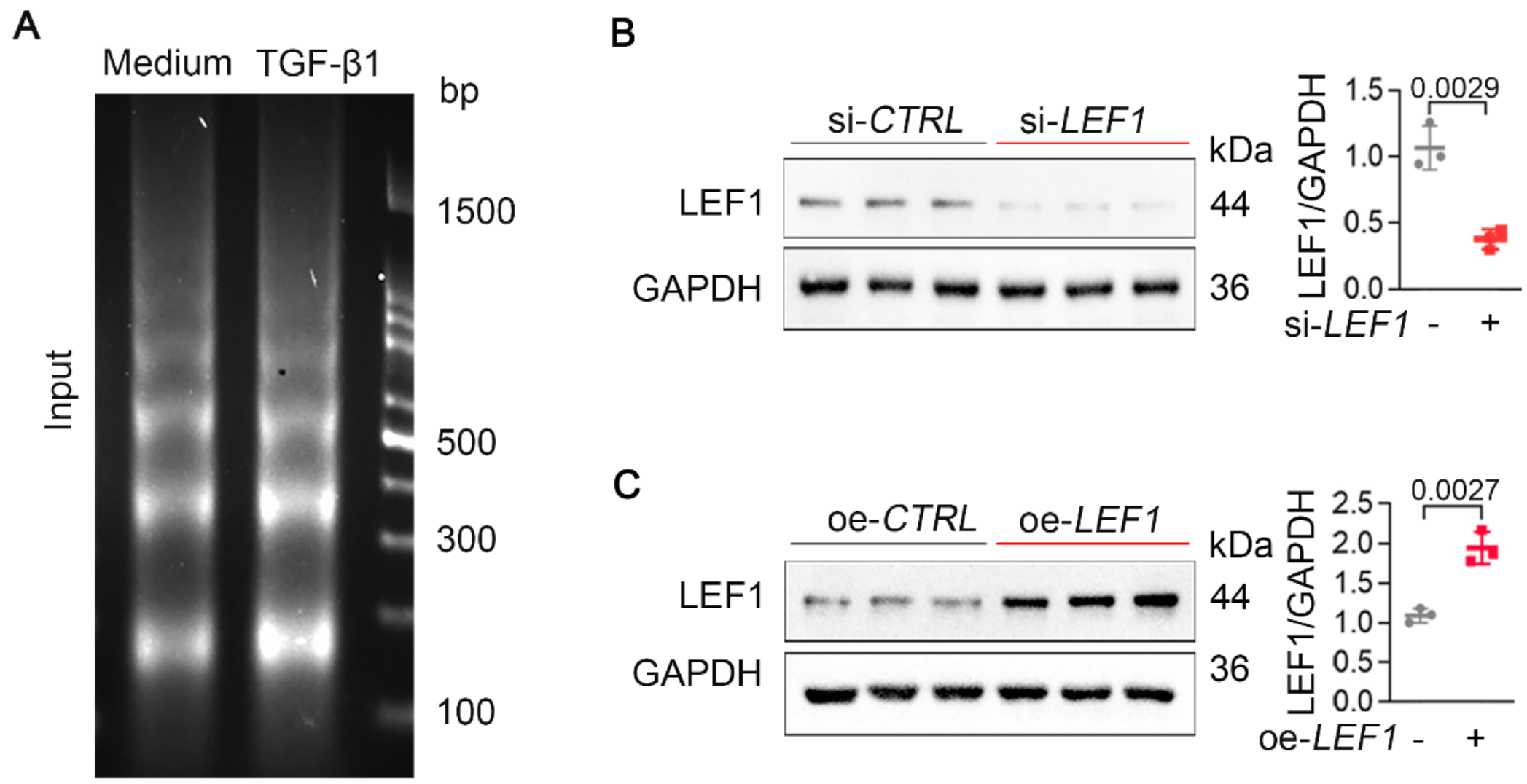

Figure S1. LEF1 represses JLP gene transcription. (Related to Figure 1).

(**A**) Agarose gel electrophoresis was performed to detect the effect of sonication of DNA samples. (**B**) Western blotting analysis of LEF1 in HK-2 cells transfected with either *LEF1* siRNA or control siRNA. The relative protein abundance was determined by normalization with GAPDH (n=3 independent experiments). (**C**) Western blotting analysis of LEF1 in HK-2 cells transfected with either the pcDNA (oe-*CTRL*) or pcDNA-LEF1 (oe-*LEF1*) plasmid. The relative protein abundance was determined by normalization with GAPDH (n=3 independent experiments). Statistical analysis was performed using two-tailed Student's t-test (panel B and C). Data are mean ± SD. NS, no significance $P \geqslant 0.05$.

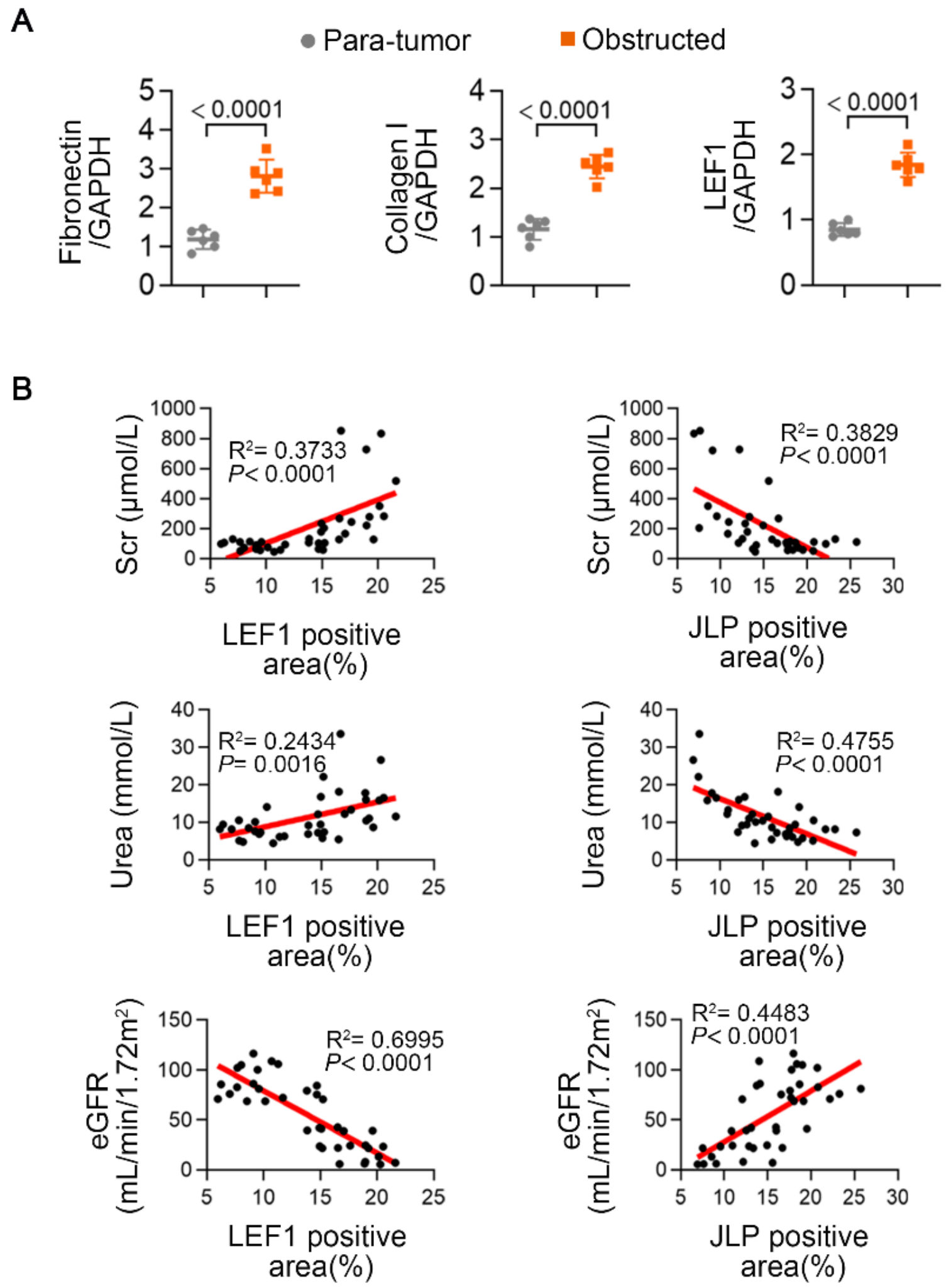

Figure S2. LEF1 expression is elevated during CKD progression. (Related to Figure 2).

(**A**) Quantification of fibrosis-related molecules (Fibronectin and Collagen I) and LEF1. The fold changes of target proteins expression levels were normalized by GAPDH (n=6 per group). (**B**) Left panel: Correlation of renal LEF1 expression with serum creatinine (Scr), and blood urea nitrogen, and estimated glomerular filtration rate (eGFR) in CKD patients. Right panel: Correlation of renal JLP expression with serum creatinine (Scr), and blood urea nitrogen, and estimated glomerular filtration rate (eGFR) in CKD patient samples and adjacent normal tissues (n=38). Statistical analysis was performed using two-tailed Student's t-test (panel A) and linear regression analysis (panel B). Data are mean ± SD.

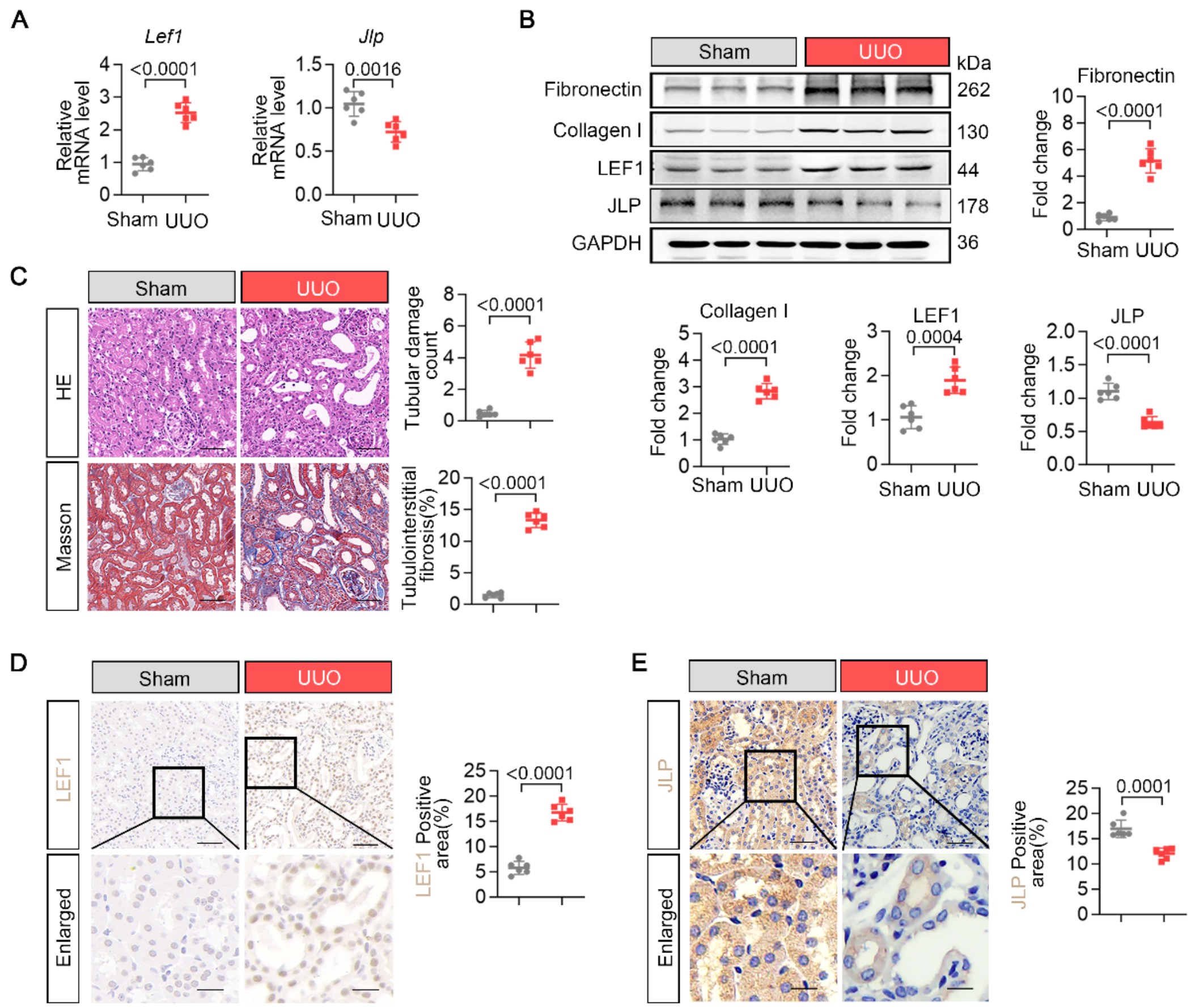

Figure S3. LEF1 expression is elevated in UUO mice models accompanied by reduced expression of JLP. (Related to Figure 2).

(**A**) Quantitative real-time PCR analysis of relative *Lef1* and *Jlp* mRNA levels in kidney tissues of the indicated groups (n=6 mice per group). *Lef1* mRNA levels were normalized to *Gapdh* mRNA levels. (**B**) Western blot analysis of fibrosis-related molecules, LEF1, and JLP in kidney tissues (n=6 mice per group). The fold changes in protein expression were normalized to GAPDH. (**C**) Representative images of HE and Masson's trichrome staining of kidney sections from Sham and UUO groups with quantification of tubular damage score and tubulointerstitial fibrosis (n=6 mice per group). Scale bar=50 μm. (**D** and **E**) Immunohistochemical images of LEF1 and JLP in mice kidneys of the indicated groups. Scale bar=50 μm. Scale bar=20 μm for the enlarged insets. (n=6 mice per group). Data are presented as mean ± SD and two-tailed unpaired Student's t-test was used for statistical analysis.

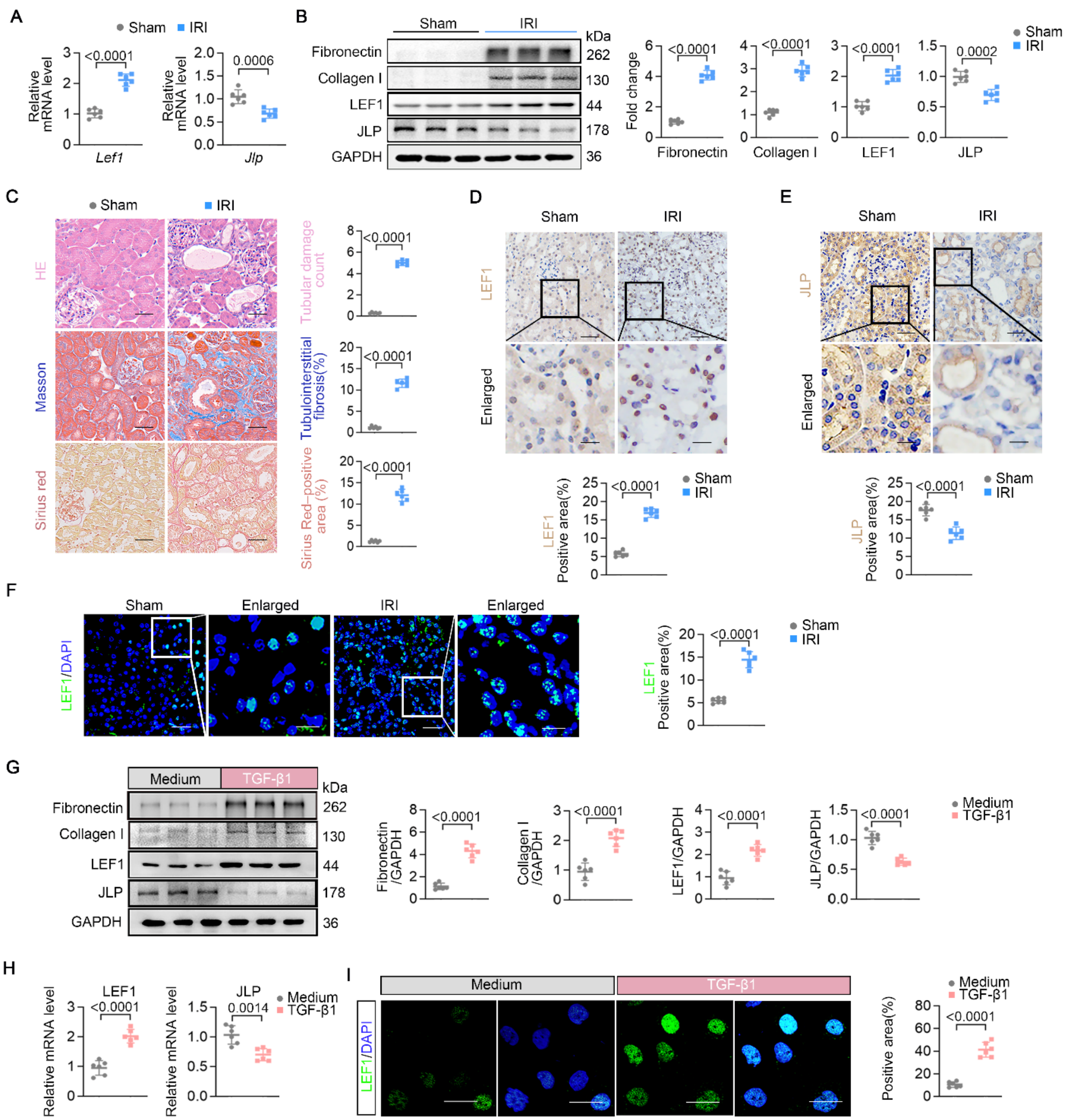

Figure S4. JLP expression is inversely correlated with LEF1 level in fibrotic models.

(Related to Figure 2).

(**A**) Quantitative real-time PCR analysis of relative *Lef1* and *Jlp* mRNA levels in kidney tissues of the indicated groups (n=6 mice per group). (**B**) Western blot analysis of fibrosis-related molecules (Fibronectin, Collagen I), LEF1, and JLP expression in kidney tissues of the indicated groups (n=6 mice per group). The fold changes in protein expression were normalized to GAPDH. (**C**) Representative images of HE and Masson's trichrome, Sirius red staining of kidney sections from Sham and uIRI (28d) groups. Quantification of tubular damage score and tubulointerstitial fibrosis

is shown at the right of images (n=6 mice per group). Scale bar = 50 µm. (**D** and **E**) Immunohistochemical and IF images of indicated protein in mice kidneys. The quantification of positive area was shown in the below panel (n=6 mice per group). Scale bar=50 µm. Scale bar=20 µm for the enlarged insets. (**F**) Immunofluorescence images of LEF1 in the indicated groups. n=6 mice per group. Scale bar = 50 µm. Scale bar=20 µm for the enlarged insets. (**G**) Western blot analysis of the expression of fibrosis-related molecules and LEF1 in HK-2 cells treated with TGF-β1 (n=6 independent experiments). Fold changes were normalized to GAPDH. (**H**) Quantitative real-time PCR analysis of relative LEF1 and JLP mRNA levels in HK-2 cells treated with TGF-β1 (n=6 independent experiments). LEF1 and JLP mRNA levels were normalized to GAPDH mRNA levels. (**I**) Immunofluorescence staining for LEF1(green) in HK-2 cells treated with or without TGF-β1. Nuclei stained with the DNA dye DAPI (blue). Quantification of the positive area is shown in the adjacent panel (n=6 independent experiments). Scale bar = 50 µm. Statistical analysis was performed using two-tailed Student's t-test. Data are mean ± SD. NS no significance $P \geq 0.05$.

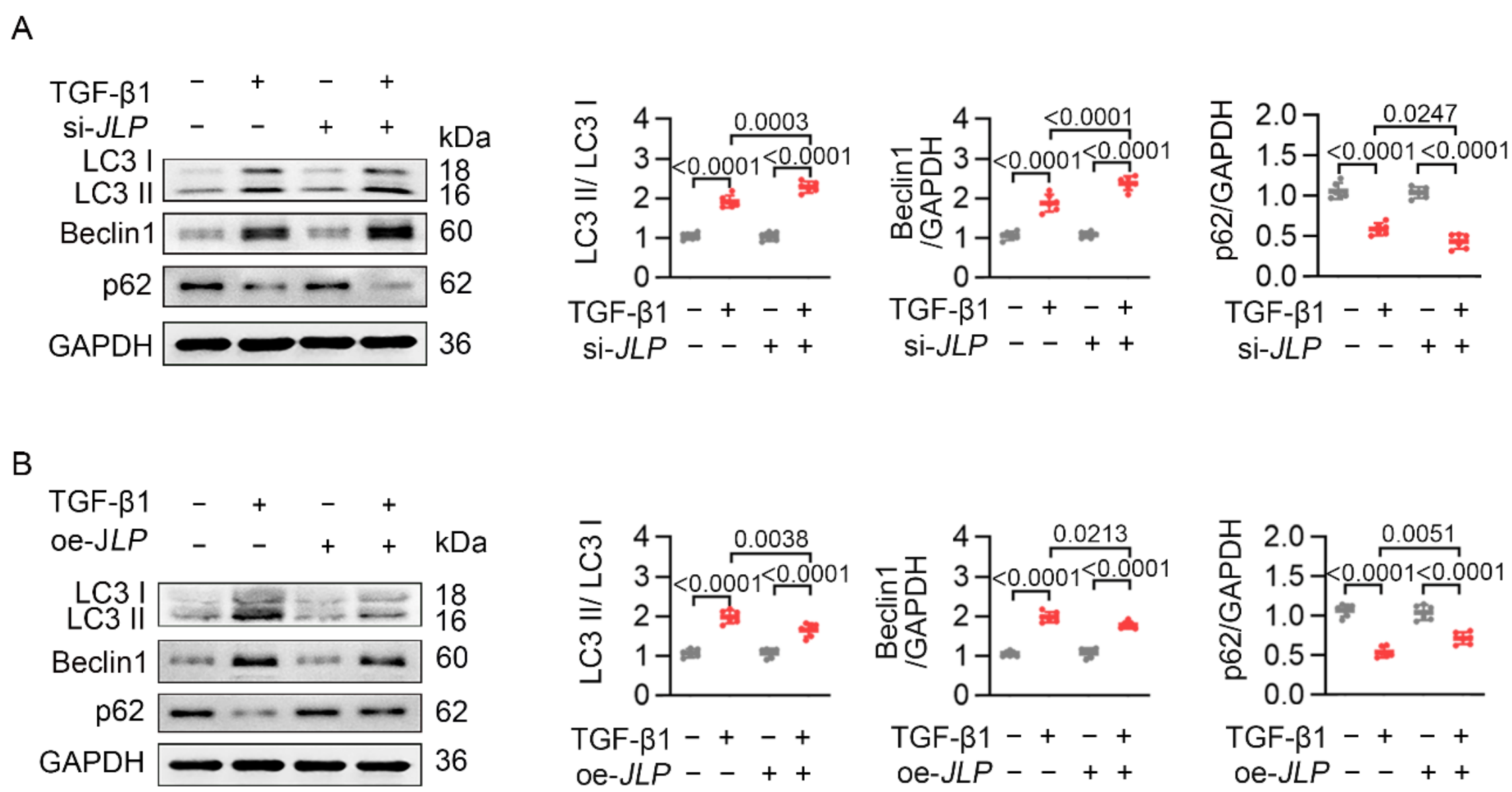

Figure S5. JLP mediates TECs autophagy. (Related to Figure 4).

(**A** and **B**) Immunoblotting analysis were performed to detect the expression of LC3 I, LC3 II, Beclin 1, p62 in HK-2 cells from the indicated groups. The fold changes of target proteins expression levels were normalized by GAPDH (n=6 independent experiments). Statistical analysis was performed using one-way ANOVA followed by Tukey's multiple-comparison test. Data are mean ± SD.

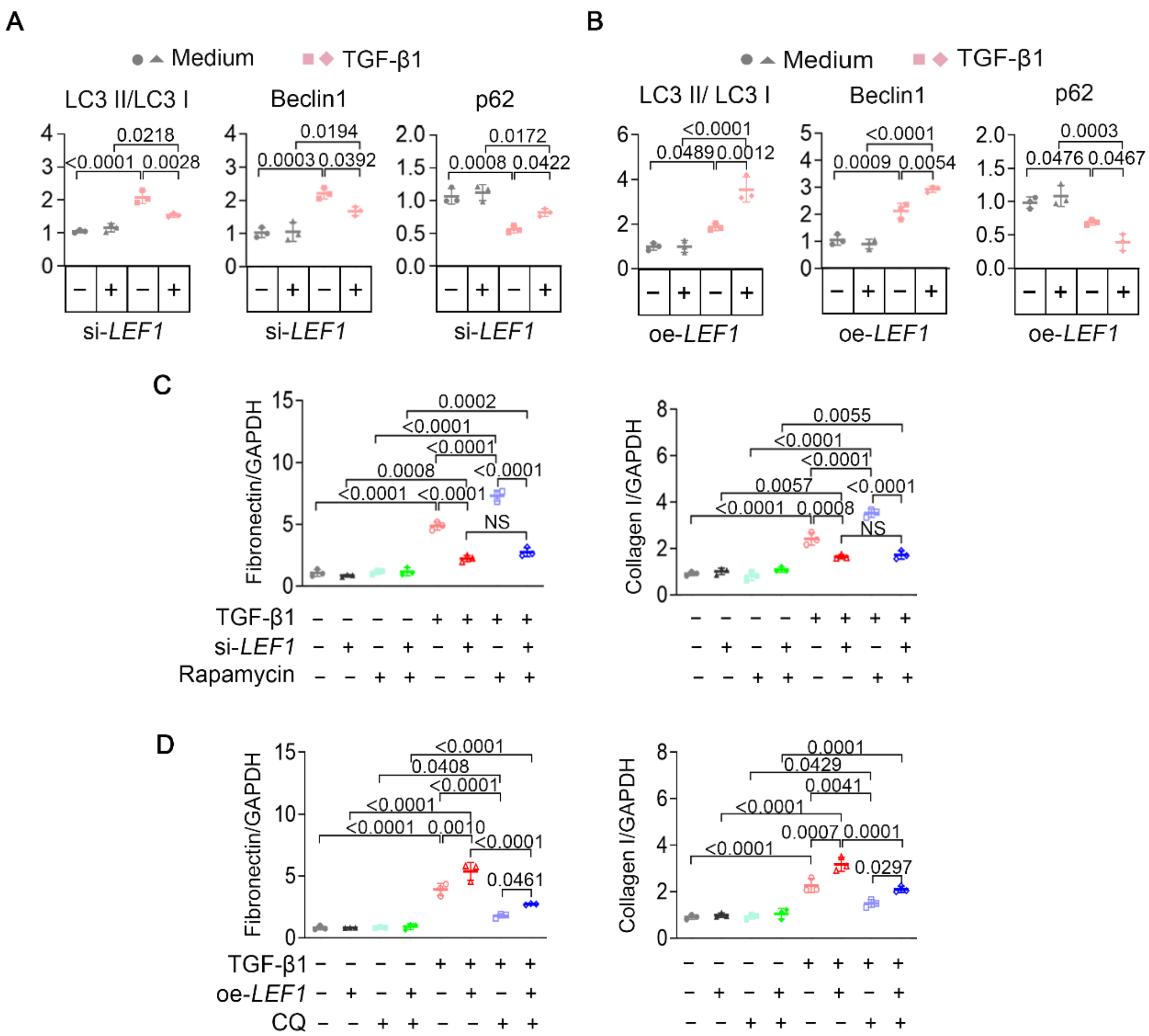

Figure S6. LEF1 regulates TECs autophagy and fibrotic response. (Related to Figure 4).

(**A**) Quantification of LC3, Beclin1, and p62 protein levels in HK-2 cells transfected with *LEF1* siRNA or control siRNA, following TGF-β1 stimulation. The relative protein abundance was normalized to GAPDH (n=3 independent experiments). (**B**) Quantification of LC3, Beclin1, and p62 protein levels in HK-2 cells transfected with either oe-Ctrl or oe-*LEF1* plasmid, following TGF-β1 stimulation. The relative protein abundance was normalized to GAPDH (n=3 independent experiments). (**C**) Quantification of Fibronectin and Collagen I in HK-2 cells transfected with either *LEF1* siRNA or control siRNA, following rapamycin and TGF-β1 stimulation. (n=3 independent experiments). (**D**) Quantification of Fibronectin and Collagen I in HK-2 cells transfected with either oe-Ctrl or oe-*LEF1* plasmid, following CQ and TGF-β1 stimulation. (n=3 independent experiments). Statistical analysis was performed using one-way ANOVA followed by Tukey's multiple-comparison test. Data are mean ± SD. NS, no significance $P \geq 0.05$.

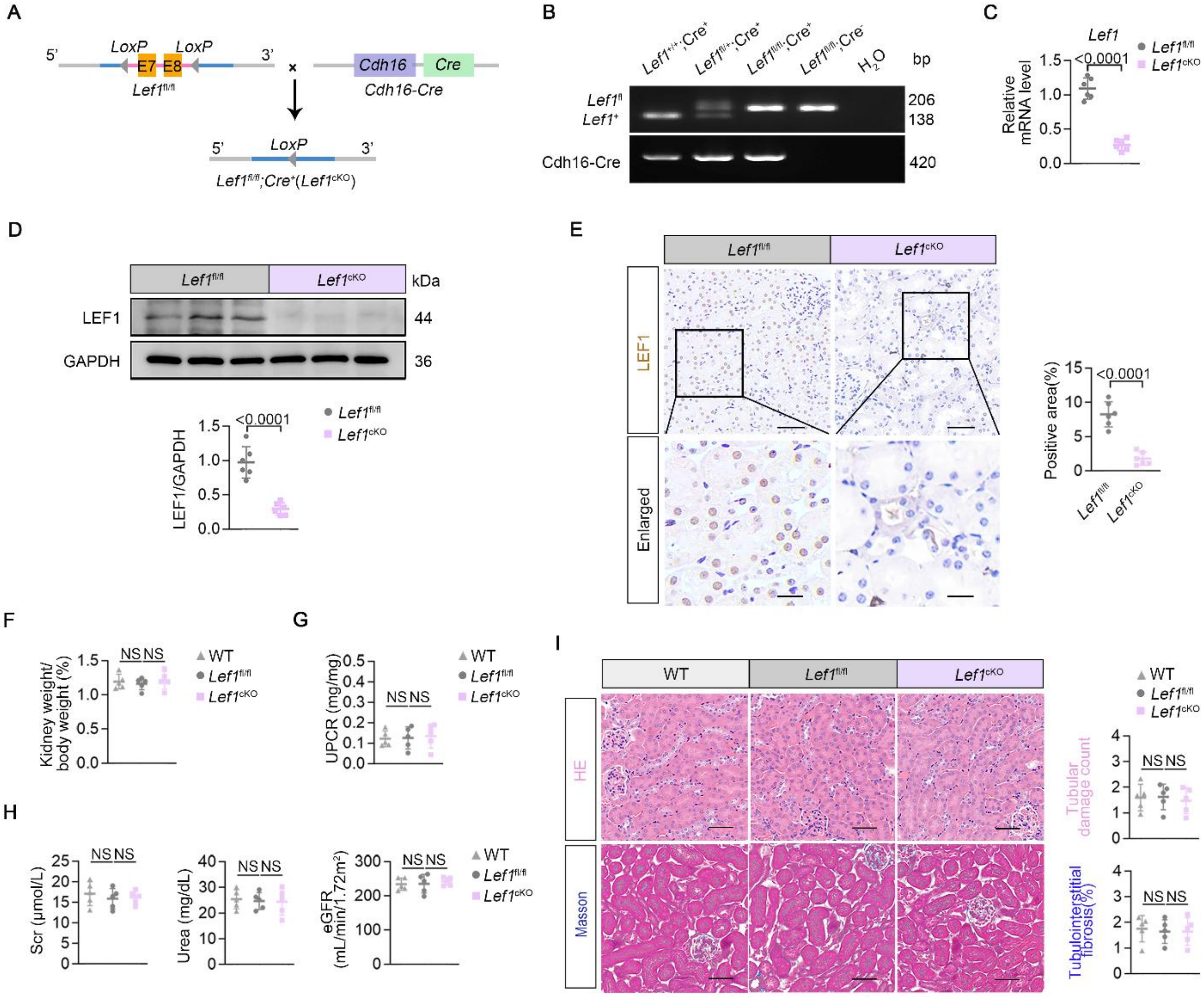

Figure S7. Generation of TEC-specific *Lef1* knockout mouse. (Related to Figure 5).

(**A**) Schematic diagram of the generation of TEC-specific *Lef1* knockout mouse. (**B**) Genotyping of *Lef1* and *Cre* mice. PCR assay of mice from the indicated groups. (**C-E**) RT-qPCR assay, western blotting, IHC staining of LEF1 in kidney tissues from *Lef1$^{fl/fl}$ and Lef1^{cKO}* mice. n=6 mice per group. Scale bar = 50 µm. Scale bar= 20 µm for the enlarged insets. (**F**) Quantification of the kidney weight to body weight ratio (KW/BW) in WT, *Lef1$^{fl/fl}$ and Lef1^{cKO}* mice. n=5 mice per group. (**G**) Quantification of the urine protein-to-creatinine ratio (UPCR) in WT, *Lef1$^{fl/fl}$ and Lef1^{cKO}* mice. n=5 mice per group. (**H**) Quantifications of the levels of serum creatinine (SCr), blood urea nitrogen, and eGFR in WT, *Lef1$^{fl/fl}$ and Lef1^{cKO}* mice. n=5 mice per group. (**I**) H&E and Masson staining of kidney tissues from the indicated group. Quantification of tubular damage score, and tubulointerstitial fibrosis percentage, Scale Bar=50 µm. n=5 mice per group. Statistical analysis was performed using two-tailed Student's t-test (panel C, D, E) and one-way ANOVA analysis (panel F, G, H, I). Data are mean ± SD. NS, no significance $P \geqslant 0.05$.

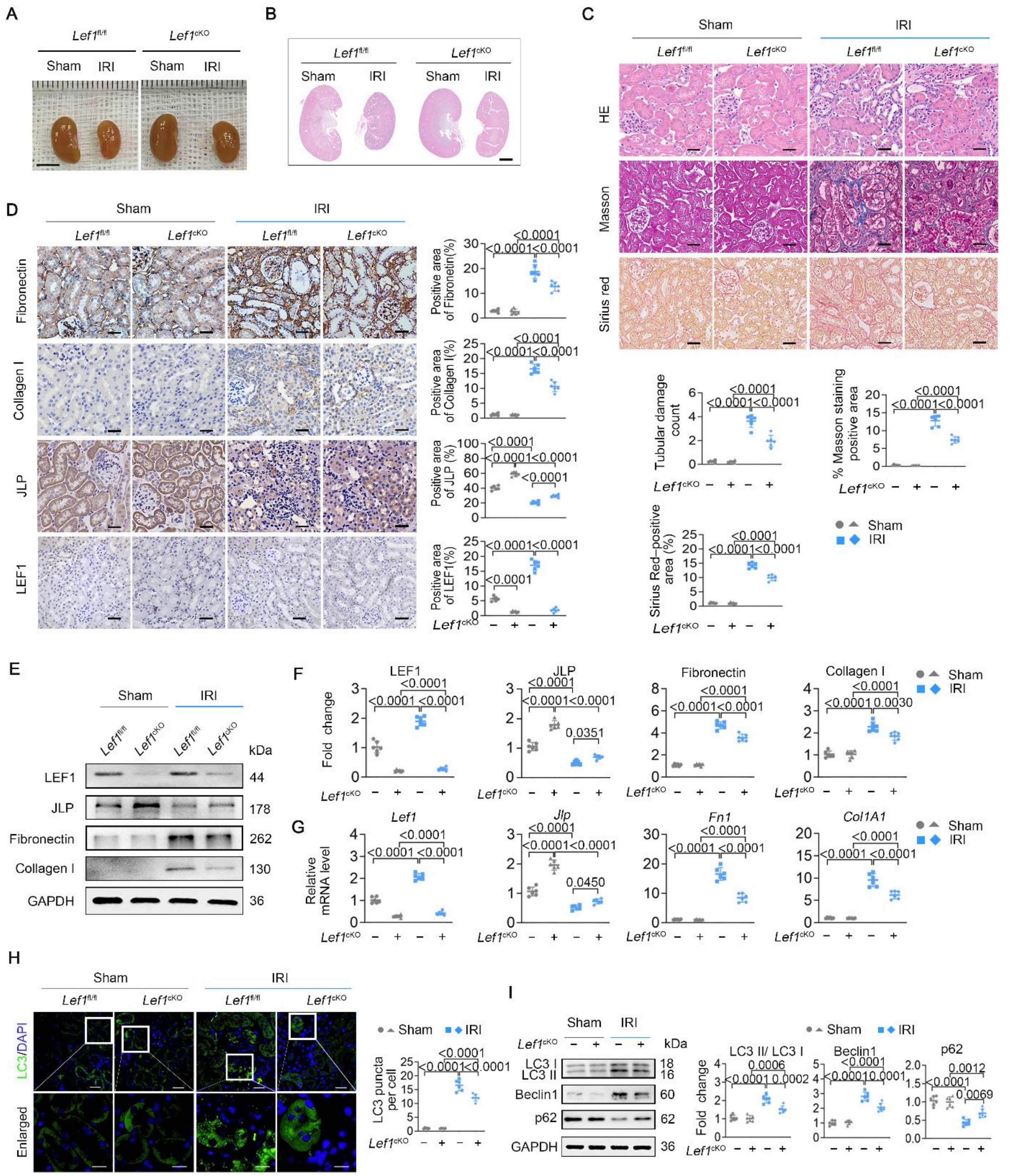

Figure S8. Renal TEC-specific *Lef1* deficiency ameliorates renal fibrosis in uIRI (28d) mouse model. (Related to Figure 5).

(**A**) Gross appearance of kidneys from the indicated groups. (**B**) Photomicrographs exhibiting the Hematoxylin and eosin (HE) staining of kidney sections from the indicated groups. Scale Bar=2 mm. (**C**) H&E, Masson and Sirius red staining of kidney tissues from the indicated group.

Quantification of tubular damage score, and tubulointerstitial fibrosis percentage, Scale Bar=50 μm. n=6 mice per group. (**D**) IHC staining of the protein expression of the related molecules in kidney tissues from the indicated group. Quantification the expression of Fibronectin, Collagen I, JLP, LEF1 based on immunohistochemistry staining. Scale Bar=50 μm. n=6 mice per group. (**E** and **F**) Western blot analysis and quantitative data of LEF1, JLP, Fibronectin, and Collagen-I of kidney tissues in the indicated groups. The differences in protein expression were normalized by GAPDH (right panel). n=6 mice per group. (**G**) The relative mRNA expressions in kidney tissues in the four groups were calculated by normalization to GAPDH mRNA. n=6 mice per group. (**H**) Immunofluorescence staining images of LC3 in kidney sections from indicated groups. Densitometric analysis of LC3 signals. Scale bar=50 μm. Scale bar=20 μm for the enlarged insets. (**I**) Immunoblot analysis of LC3, Beclin1, and p62 in Sham or uIRI mouse kidneys from *Lef1*^{cKO} and *Lef1*$^{fl/fl}$ mice. n=6 mice per group. Statistical analysis was performed using one-way ANOVA followed by Tukey's multiple-comparison test (panel C, D, F, G, H, I). Data are mean ± SD.

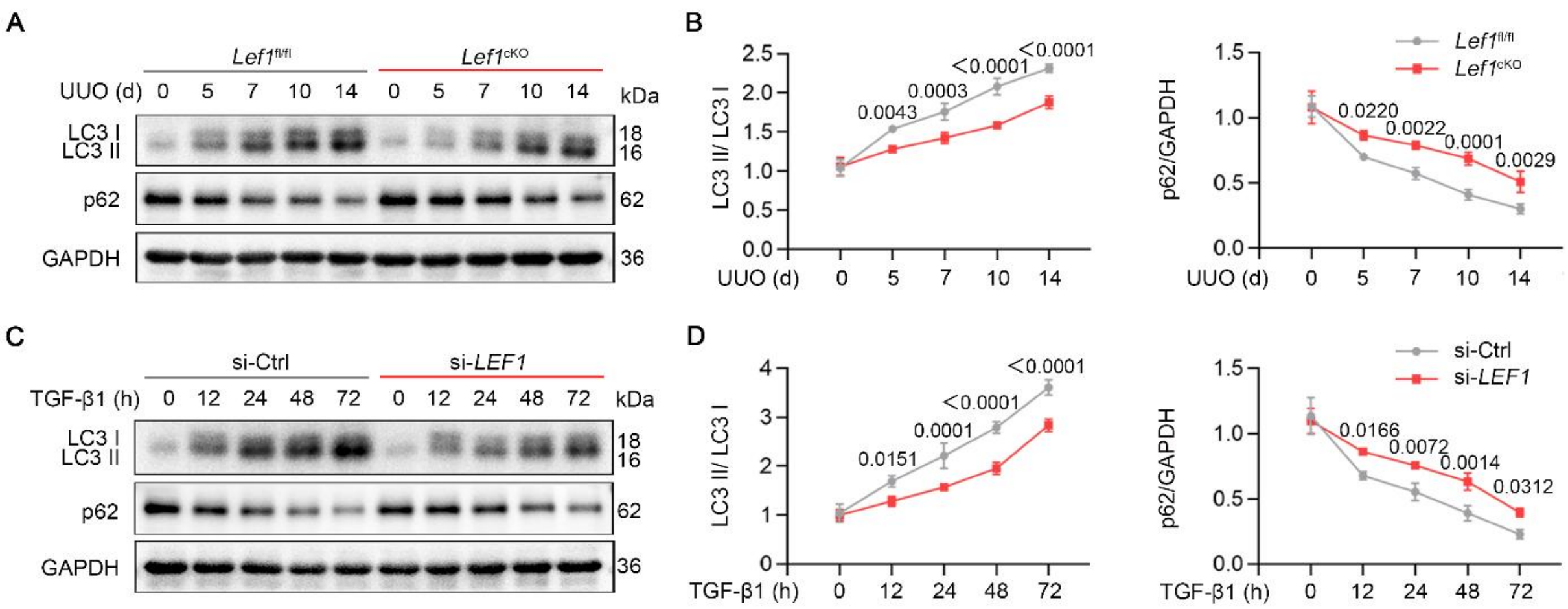

Figure S9. LEF1 regulates autophagy dynamics in the UUO mouse model and in HK-2 cells following TGF-β1 stimulation. (Related to Figure 5).

(**A** and **B**) Western blot and quantification of LC3 and P62 in kidney tissues from the indicated groups. n=3 mouse per group. (**C** and **D**) Western blot and quantification of LC3 and P62 of HK-2 cells in the indicated groups. n=3 independent experiments. Statistical analysis was performed using two-tailed Student's t-test (panel B, D). Data are mean ± SD.

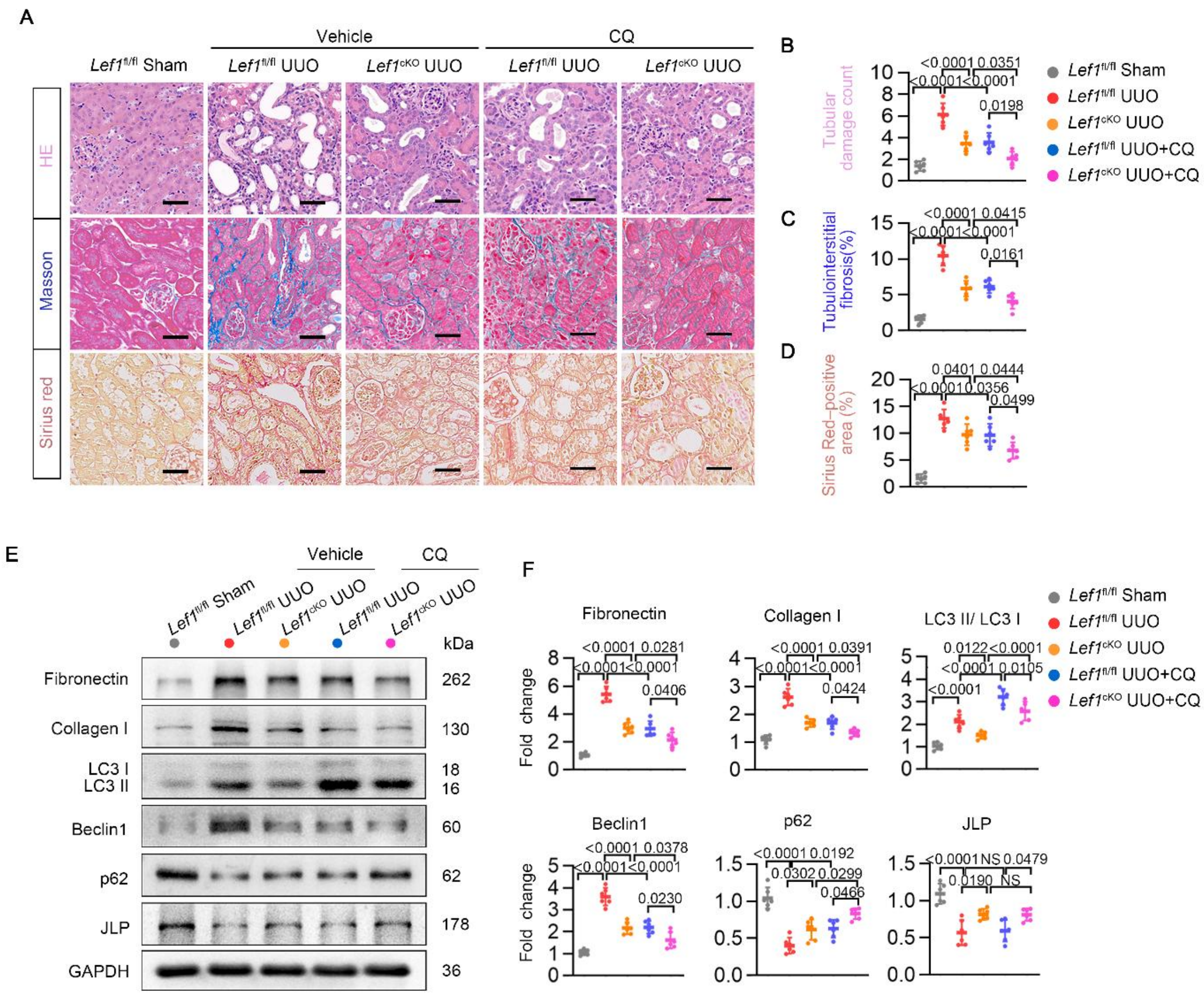

Figure S10. Administration of Chloroquine attenuates renal fibrosis in the UUO model.

(Related to Figure 5).

(**A**) H&E, Masson staining and Sirius red staining of kidney tissues from the indicated group. Scale Bar=50 μm. n=6 mice per group. (**B-D**) Tubular damage score was quantified from H&E staining. The percentage of tubulointerstitial fibrosis was quantified from Masson trichrome- or Sirius Red-stained kidney sections using ImageJ. n=6 mice per group. (**E** and **F**) Western blot analysis and quantitative data of Fibronectin, Collagen I, LC3, Beclin1, p62, and JLP in kidney tissues from the indicated groups. n=6 mice per group. Statistical analysis was performed using one-way ANOVA followed by Tukey's multiple-comparison test (panel B, C, D, F). Data are mean ± SD.

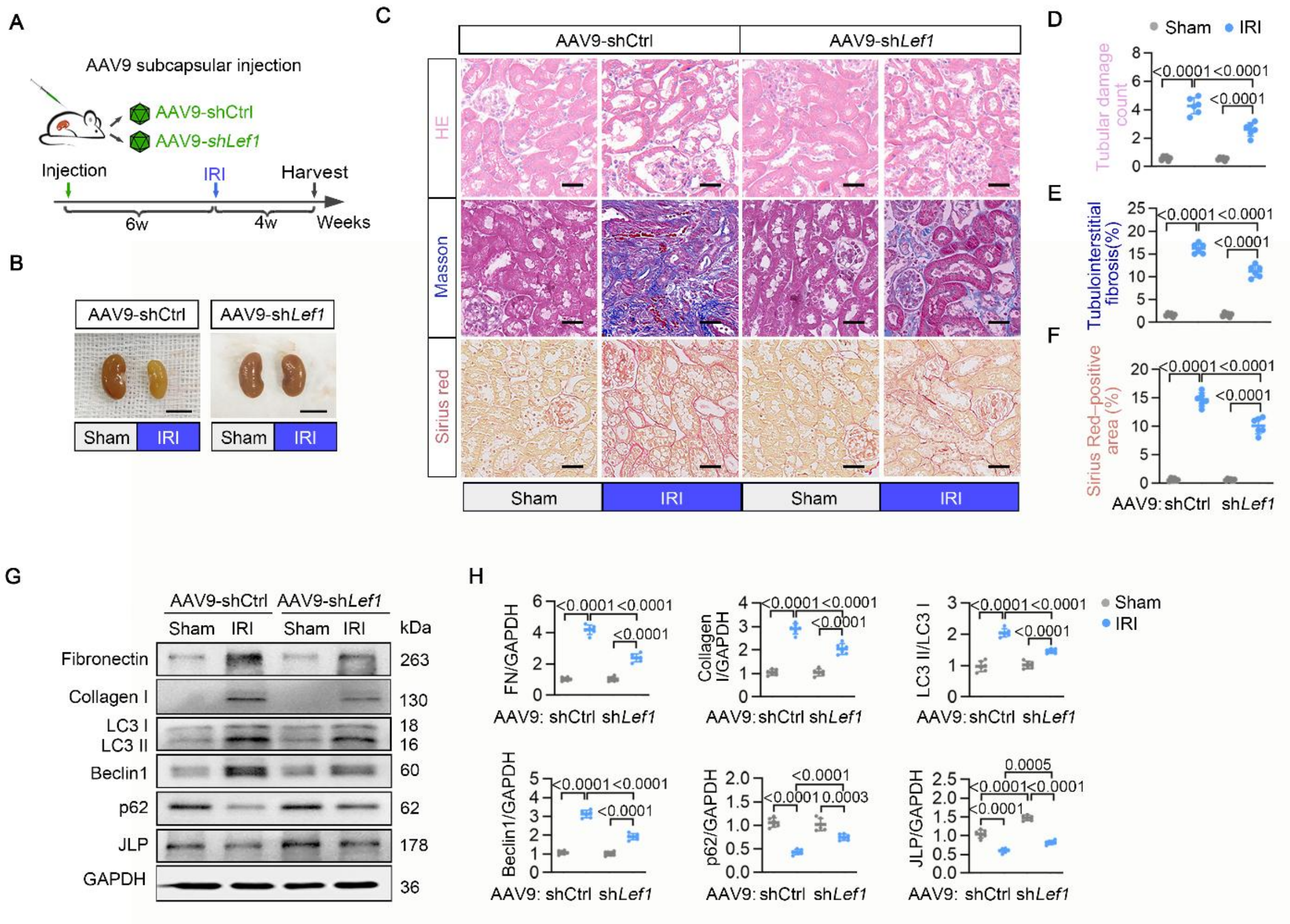

Figure S11. AAV9-*shLef1* gene therapy mitigates uIRI-induced renal fibrosis. (Related to Figure 6).

(**A**) Schematic of experimental design. Renal subcapsular delivery of the AAV9-shCtrl or AAV9-sh*Lef1* to wild-type C57BL/6 mice at 6 weeks of age. After the delivery for six weeks, the mice were subjected to uIRI surgery. (**B**) Gross appearance of kidneys from the indicated groups. Scale Bar=5 mm. (**C-F**) H&E, Masson staining and Sirius red staining of kidney tissues from the indicated group.Quantification of tubular damage score, and tubulointerstitial fibrosis percentage, Scale Bar=50 µm. n=6 mice per group. (**G** and **H**) Western blot analysis and quantitative data of Fibronectin, Collagen-I, LC3, Beclin I, p62 and JLP of kidney tissues in the indicated groups. n=6 mice per group. Statistical analysis was performed using one-way ANOVA followed by Tukey's multiple-comparison test (panel D, E, F, H). Data are mean ± SD.

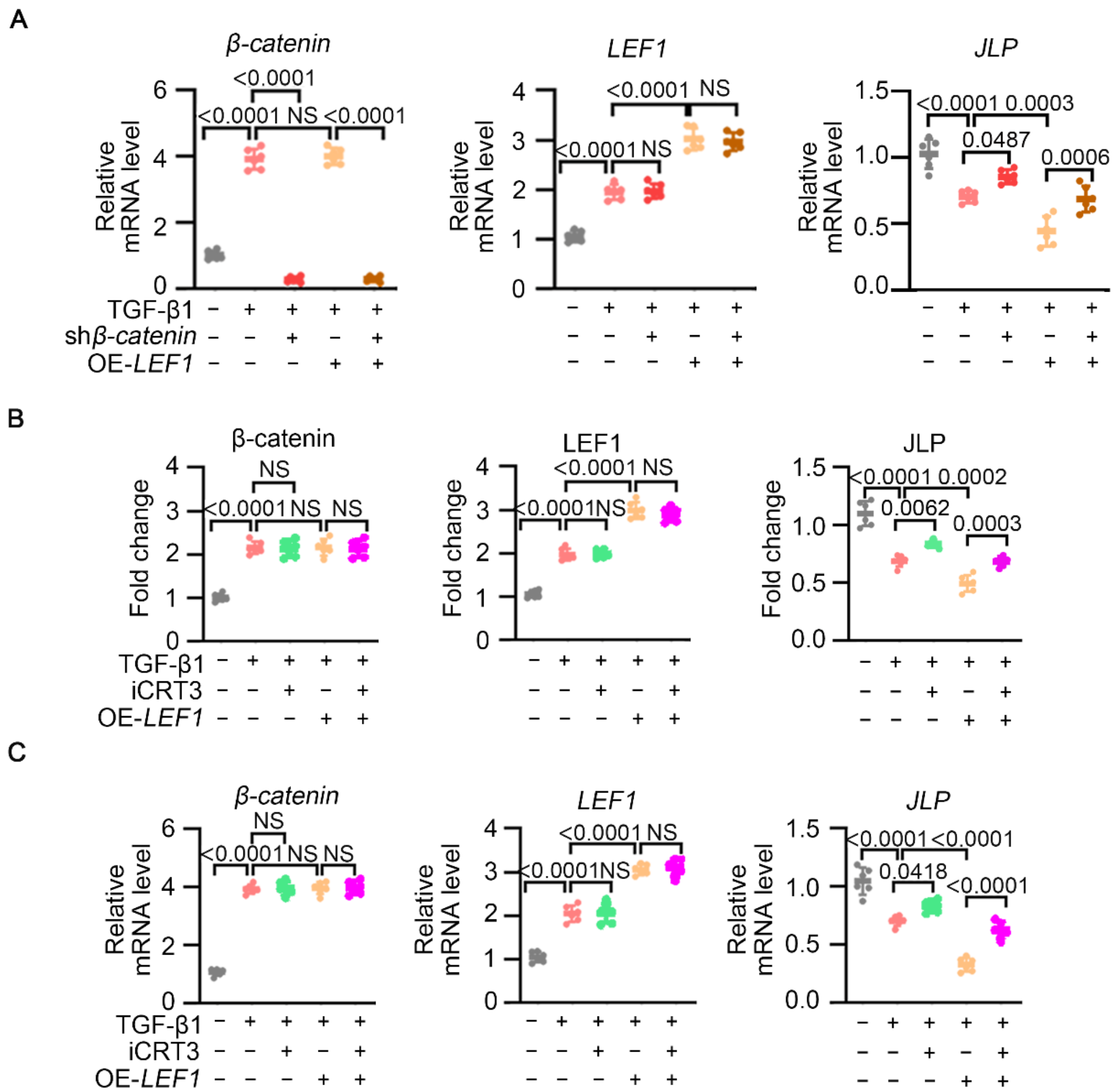

Figure S12. Inhibition of LEF1 reduces TECs injury under TGF-β1 treatment. (Related to Figure 7).

(**A**) Abundance of the indicated mRNAs analyzed by RT-qPCR in HK-2 cells (n = 6 biologically independent samples). (**B**) Western blot quantitative data of β-catenin, LEF1, and JLP protein levels in HK-2 cells. (n = 6 biologically independent samples). (**C**) Abundance of the indicated mRNAs analyzed by RT-qPCR (n = 6 biologically independent samples). Statistical analysis was performed using one-way ANOVA followed by Tukey's multiple-comparison test. Data are mean ± SD. NS, no significance $P \geqslant 0.05$.

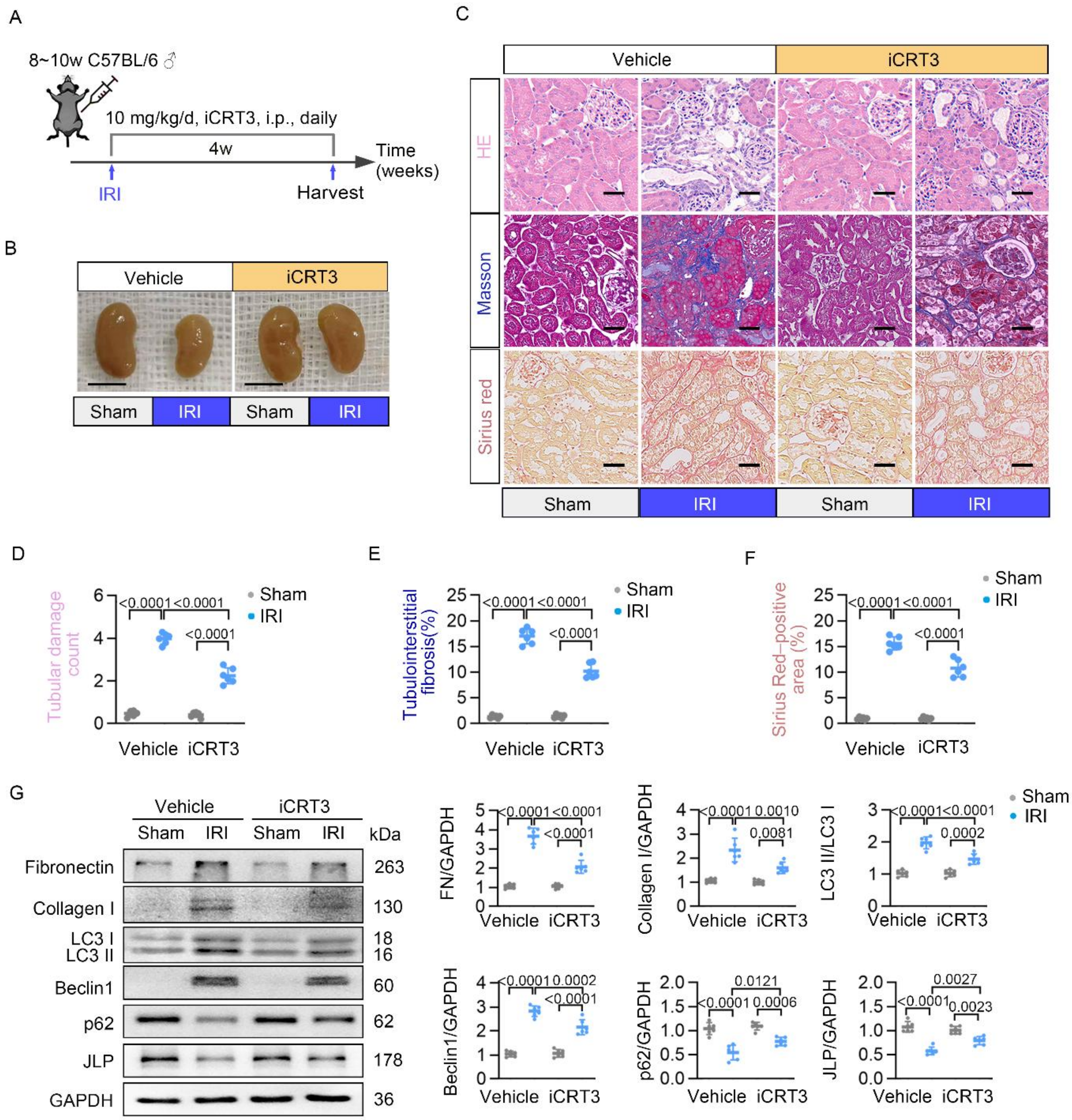

Figure S13. Pharmacological inhibition of LEF1 attenuates uIRI-induced renal fibrosis. (Related to Figure 8).

(**A**) Schematic of experimental design. Wild-type C57BL/6 mice (8-10 weeks old, male) underwent uIRI surgery and were administered daily i.p. injections of iCRT3 (10 mg/kg/d) for 4 weeks. (**B**) Gross appearance of kidneys from the indicated groups. Scale Bar=5 mm. (**C**) H&E, Masson's trichrome, and Sirius red staining of kidney tissues from the indicated group. (**D-F**) Quantification of tubular damage score, and tubulointerstitial fibrosis percentage, Scale Bar=50 μm. n=6 mice per group. (**G**) Western blot analysis and quantitative data of Fibronectin, Collagen-I, LC3, Beclin I, p62 and JLP of kidney tissues in the indicated groups. n=6 mice per group.

Statistical analysis was performed using one-way ANOVA followed by Tukey's multiple-comparison test (panel D, E, F, G). Data are mean ± SD.

Table S1. Baseline characteristics of CKD and adjacent normal tissues patients.

Sample number	Gender	Age [ys]	Serum creatinine [μmol/l]	Blood urea nitrogen [mmol/l]	eGFR	Diagnosis	CKD stage
1	male	61	98	8.21	71.01	RCC	-
2	male	56	112	10.58	82.67	RCC	-
3	female	56	106	9.48	85.71	RCC	-
4	male	70	112	7.35	81.07	RCC	-
5	female	65	130	8.19	75.97	RCC	-
6	female	67	113	8.54	68.7	RCC	-
7	female	32	52	5.14	102.19	FSGS	CKD1
8	female	45	65	7.6	116.56	FSGS	CKD1
9	male	37	73	4.77	105	IgAN	CKD1
10	female	56	59	6.13	106.04	MN	CKD1
11	male	39	45	4.47	108.92	FSGS	CKD1
12	male	31	57	6.92	100.13	MN	CKD1
13	male	29	89	10.11	86.2	IgAN	CKD2
14	female	33	102	7.28	75.44	IgAN	CKD2
15	female	36	95	6.33	72.18	MN	CKD2
16	female	45	76	14.12	68.91	IgAN	CKD2
17	male	48	104	7.38	70.67	SGN	CKD2
18	male	29	107	6.91	79.3	MN	CKD2
19	male	34	64	12.17	84.39	DN	CKD2
20	female	39	129	5.46	42.79	LN	CKD3
21	female	44	178	9.45	42.24	IgAN	CKD3
22	female	61	58	5.83	41.1	DN	CKD3
23	female	60	132	9.2	39.7	MN	CKD3
24	female	55	128	8.71	39.11	IgAN	CKD3
25	male	53	167	12.27	38.9	IgAN	CKD3
26	male	38	235	16.78	23.56	DN	CKD4

27	male	46	204	22.19	21.69	DN	CKD4
28	female	52	221	10.46	24.58	HRD	CKD4
29	male	55	269	18.17	22.11	MN	CKD4
30	female	67	278	11.06	21.8	HRD	CKD4
31	male	47	282	16.6	23.3	MN	CKD4
32	male	59	244	13.37	24.18	DN	CKD4
33	female	66	350	15.9	13.05	LN	CKD5
34	female	69	727	16.04	8.04	HRD	CKD5
35	female	65	852	33.57	5.97	SGN	CKD5
36	male	54	518	11.55	7.06	DN	CKD5
37	male	58	721	17.86	6.2	SGN	CKD5
38	female	62	834	26.69	5.48	LN	CKD5

IgAN: IgA nephropathy; MN: Membranous nephropathy; LN: lupus nephritis; FSGS: Focal segmental glomerular sclerosis; SGN: Sclerosing glomerulonephritis; DN: Diabetic Nephropathy; HRD: Hypertensive renal damage; RCC: Renal cell carcinoma. eGFR [$ml \cdot min^{-1} \cdot (1.73m^2)^{-1}$]

Table S2. Clinical information of patients with obstructive nephropathy.

Sample number	Gender	Age [ys]	eGFR [ml ·min^{-1} · (1.73m^2)$^{-1}$]	Diagnosis
1	male	53	98.17	Obstructive nephropathy
2	male	42	82.93	Obstructive nephropathy
3	female	67	75.38	Obstructive nephropathy
4	female	65	80.59	Obstructive nephropathy
5	female	54	83.33	Obstructive nephropathy
6	female	39	72.08	Obstructive nephropathy

Table S3. Primer sequences used for qRT-PCR in this study.

Target		Primer sequences (5'-3')	
LEF1	h	Forward	TGCCAAATATGAATAACGACCCA
		Reverse	GAGAAAAGTGCTCGTCACTGT
	m	Forward	TCTGGCTACATAATGATGCCCA
		Reverse	GGACATGCCTTGCTTGGAGTT
SPAG9	h	Forward	CAAGGCGGATCTAAAGCTACC
		Reverse	TTGGCGCATCTGTAACCTTCA
	m	Forward	AGGTTGCCCAAGAGACTAGGA
		Reverse	AGGAGTGGATTCAATGATTGCTT
FN1	h	Forward	CGGTGGCTGTCAGTCAAAG
		Reverse	AAACCTCGGCTTCCTCCATAA
	m	Forward	ATGTGGACCCCTCCTGATAGT
		Reverse	GCCCAGTGATTTCAGCAAAGG
COL1A1	h	Forward	GAGGGCCAAGACGAAGACATC
		Reverse	CAGATCACGTCATCGCACAAC
	m	Forward	TAAGGGTCCCCAATGGTGAGA
		Reverse	GGGTCCCTCGACTCCTACAT
GAPDH	h	Forward	ACAACTTTGGTATCGTGGAAGG
		Reverse	GCCATCACGCCACAGTTTC
	m	Forward	AGGTCGGTGTGAACGGATTTG
		Reverse	TGTAGACCATGTAGTTGAGGTCA

Table S4. Primer sequences used for ChIP-qPCR in this study.

Name	Forward primer (5'-3')	Reverse primers (5'-3')
Site1	AGGTAGACCAGGTGAGTGACAG	TGGGTAGTAGGAATGCCGA
Site2	CTGGGTTACAGGCATGAGTC	AACATAGGGCAAGGCTTTAC
Site3	CTGTGAAACGAGGTCCAAAT	GGGGTTCGGGAAGAGTG
Site4	CTCCATCTACCGCGAGTTCG	GAGTCCAGGTTCTCCAGCACA
