# Supplementary material for: A TGF-β1/LEF1/β-catenin/JLP network motif regulates autophagy and tubule injury in renal fibrosis
Source: JCI Insight. 2026 Jan 8;11(13):e196835. doi: 10.1172/jci.insight.196835 (PMC13460821; doi:10.1172/jci.insight.196835)

Full unedited gel for Figure 2B

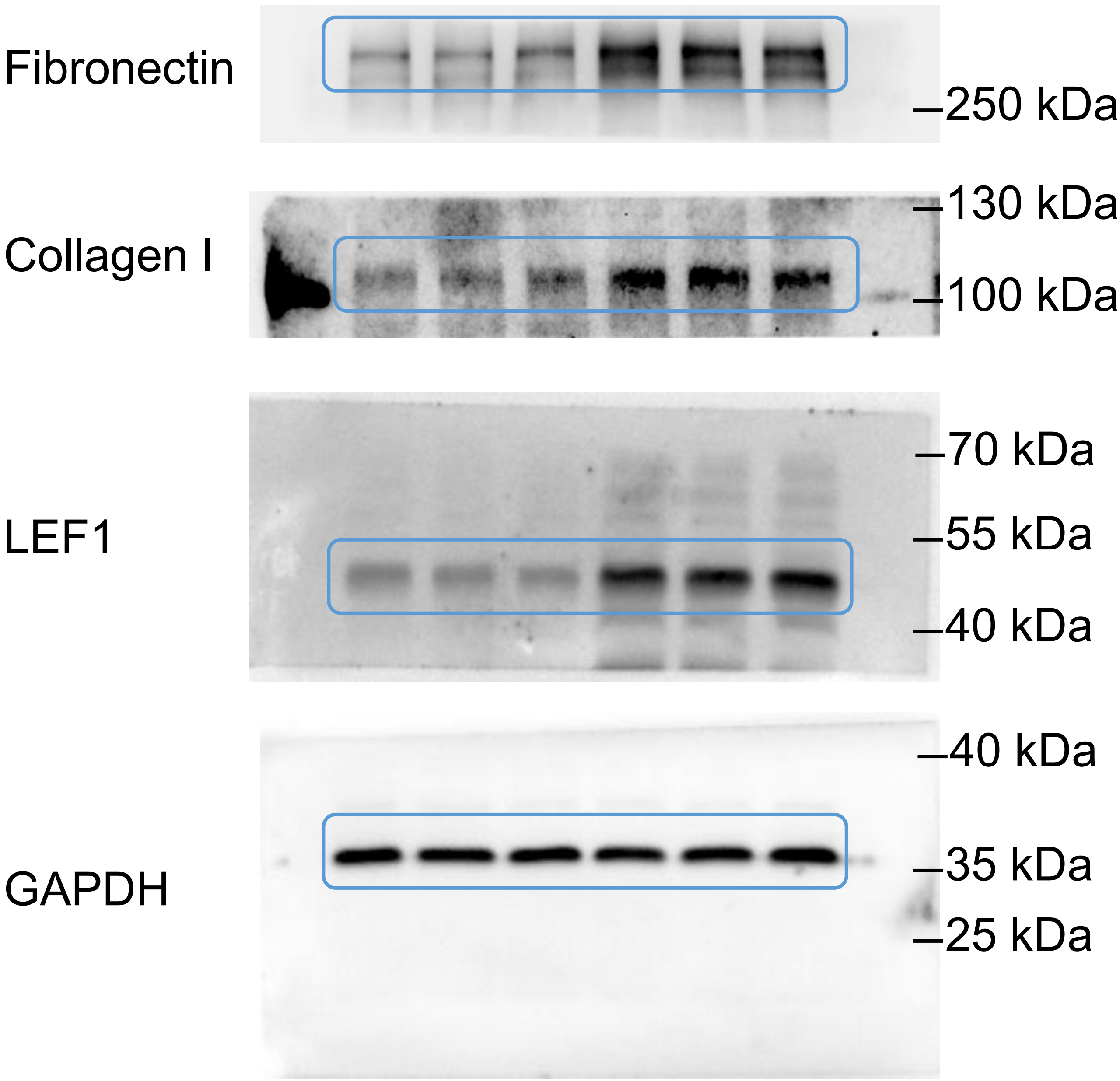

Full unedited gel for Figure 3B

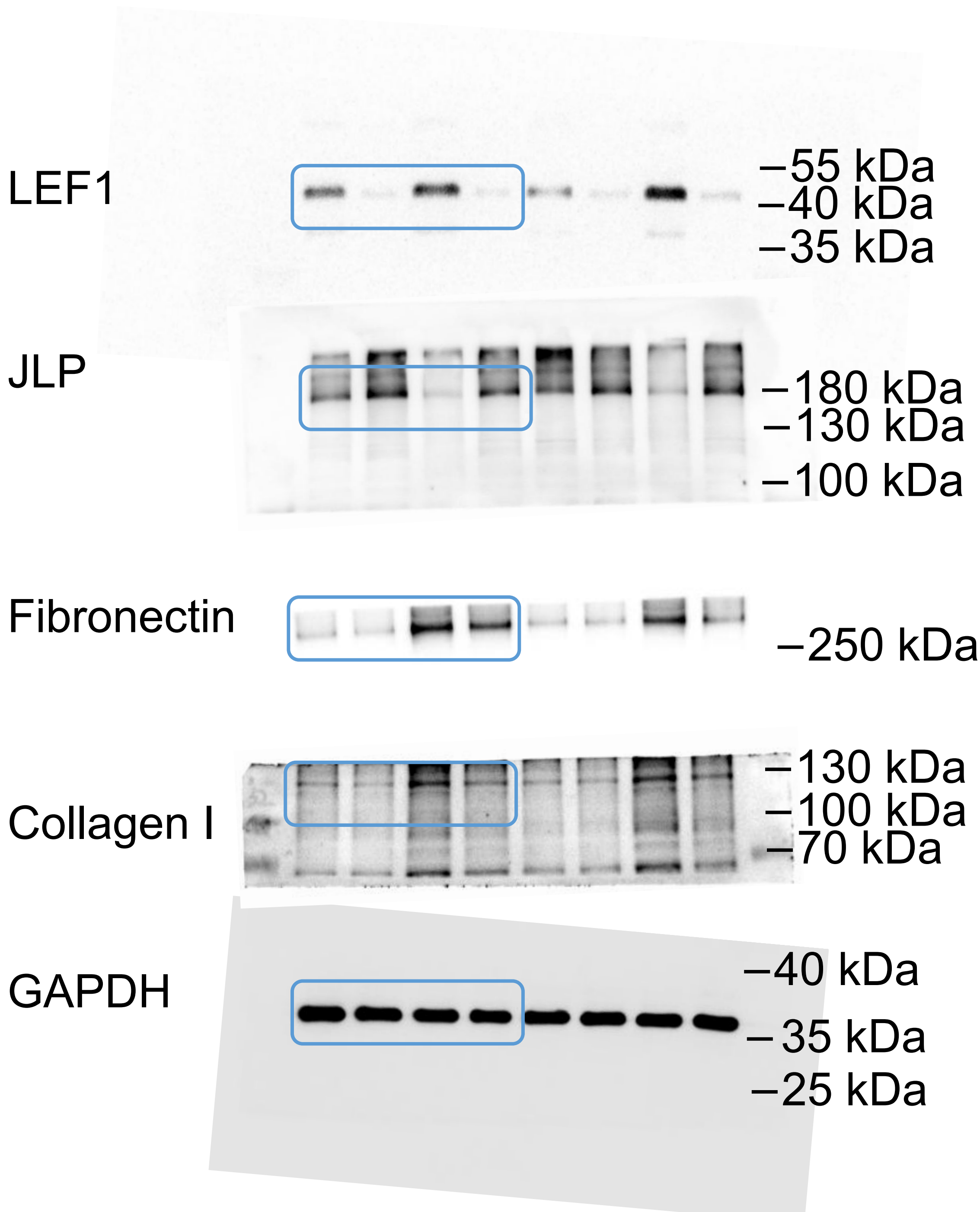

Full unedited gel for Figure 3D

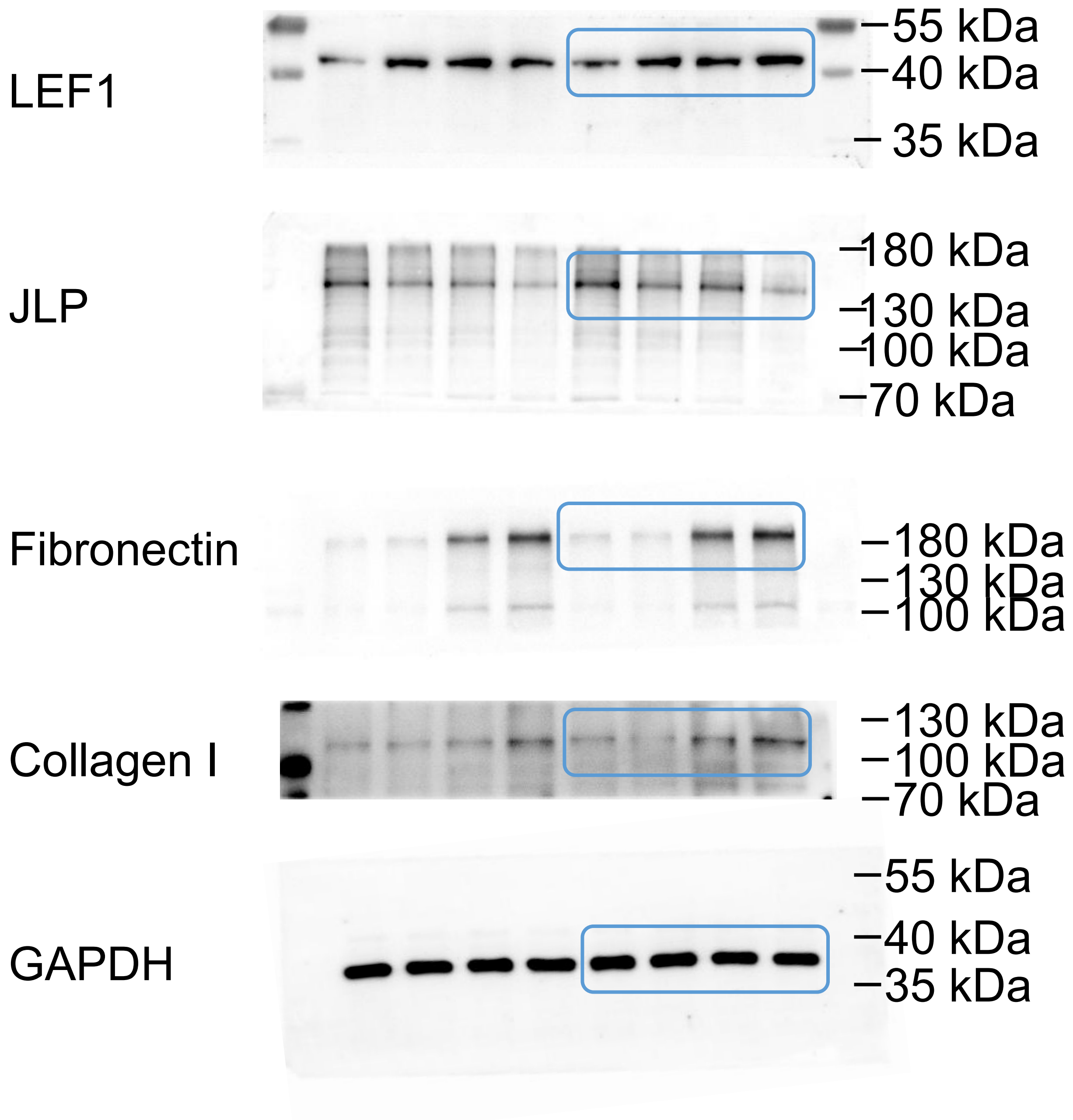

Full unedited gel for Figure 4A(Left)&4B(Right)

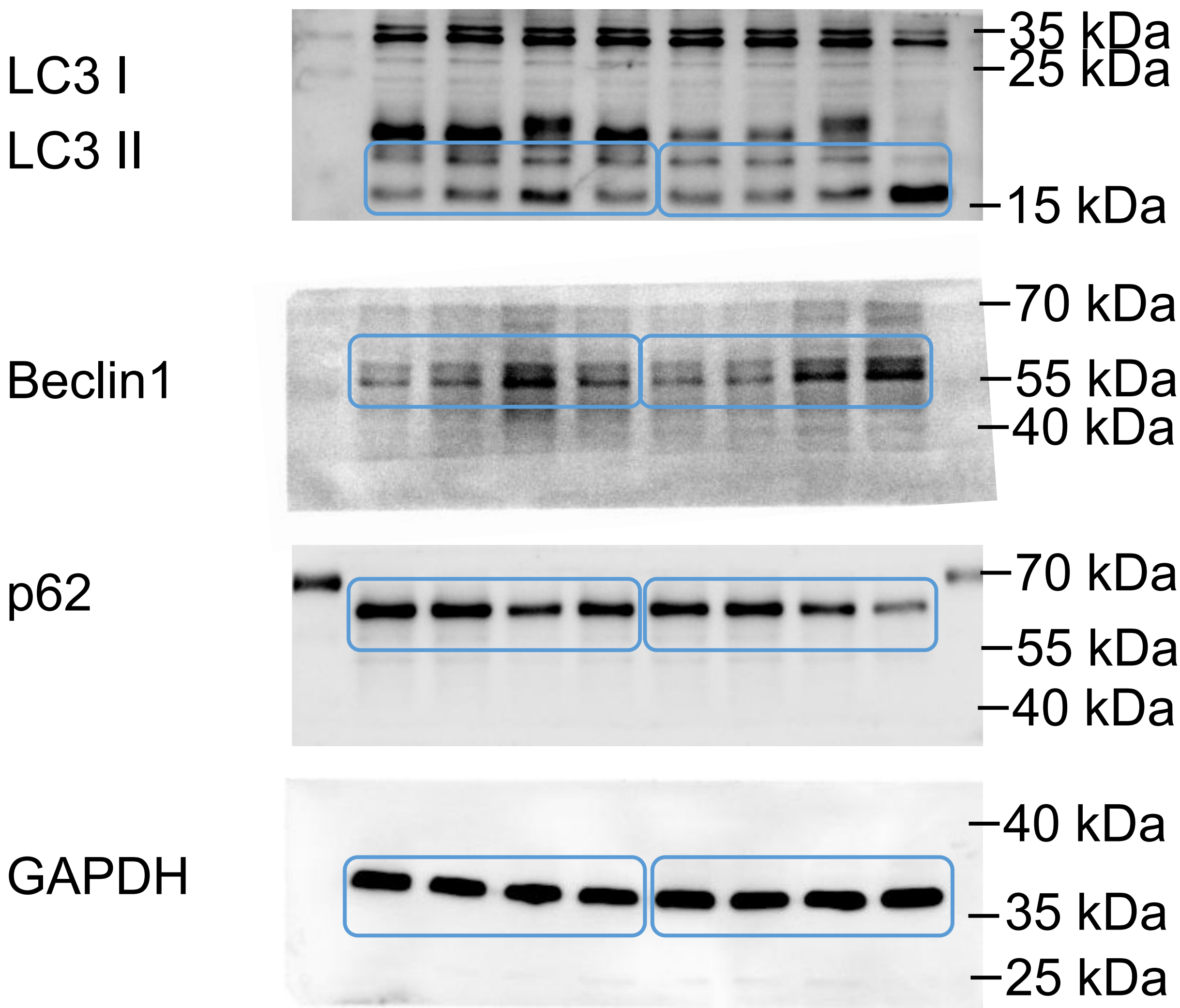

Full unedited gel for Figure 4F

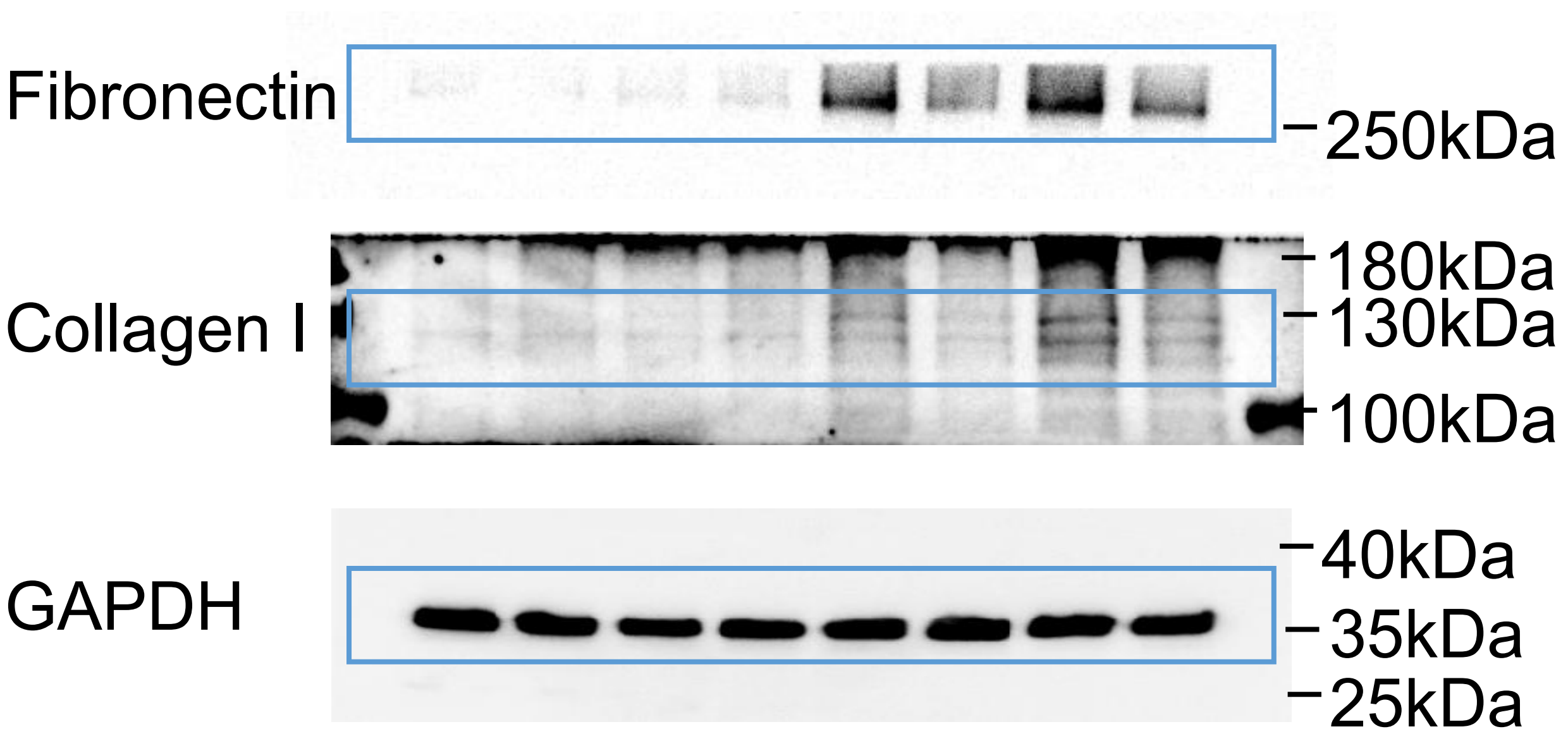

Full unedited gel for Figure 4G

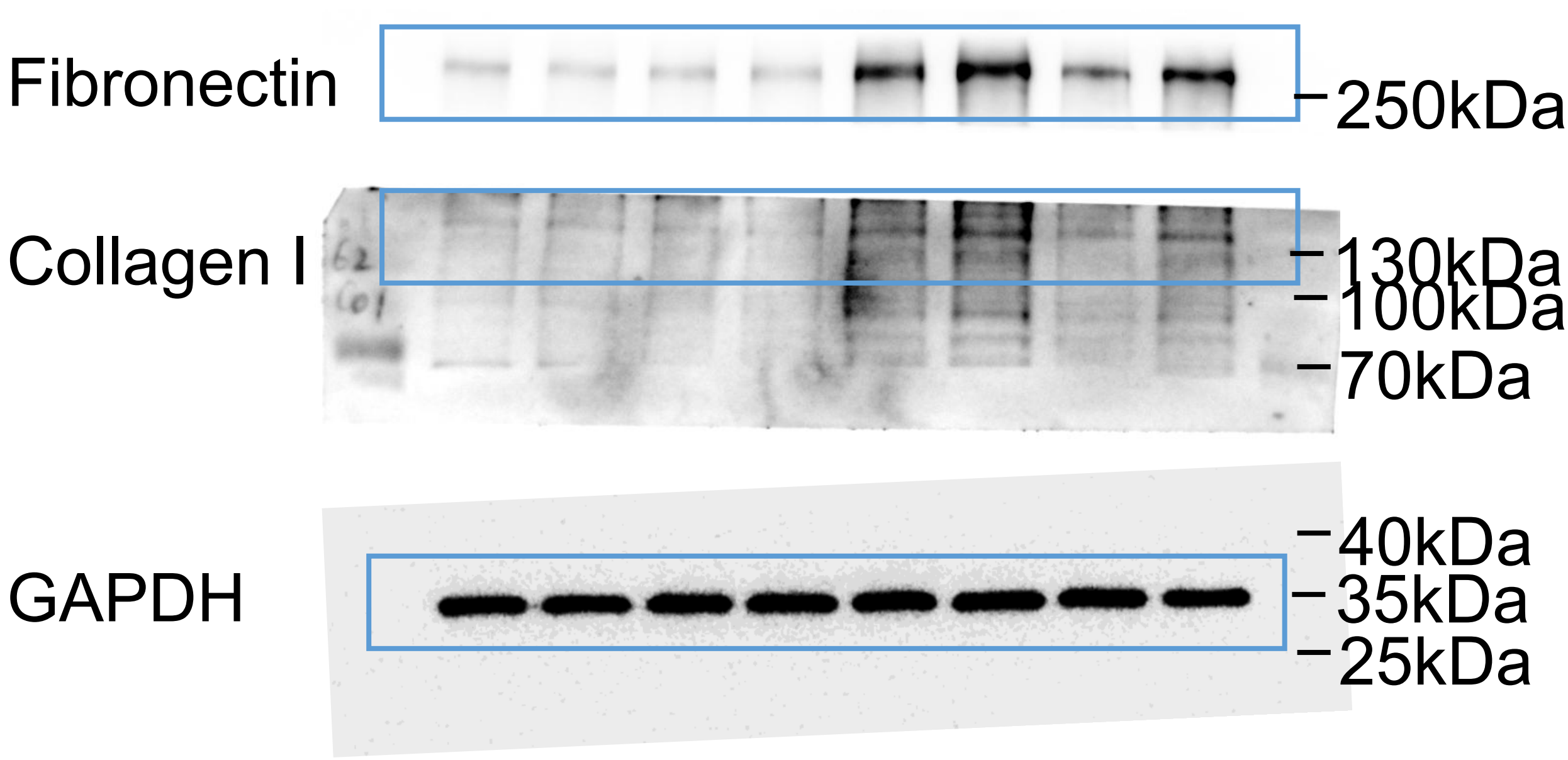

Full unedited gel for Figure 4H

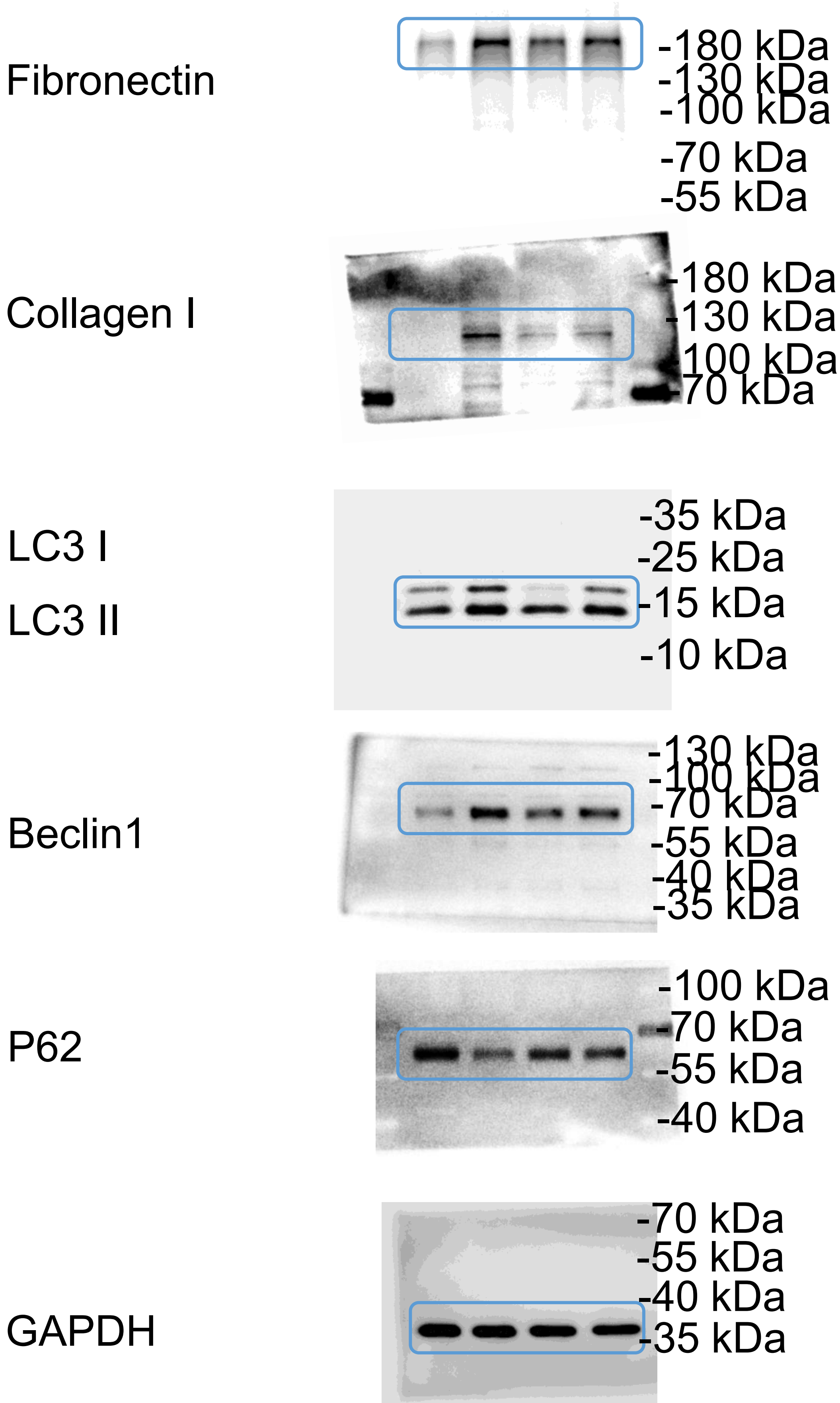

Full unedited gel for Figure 4J

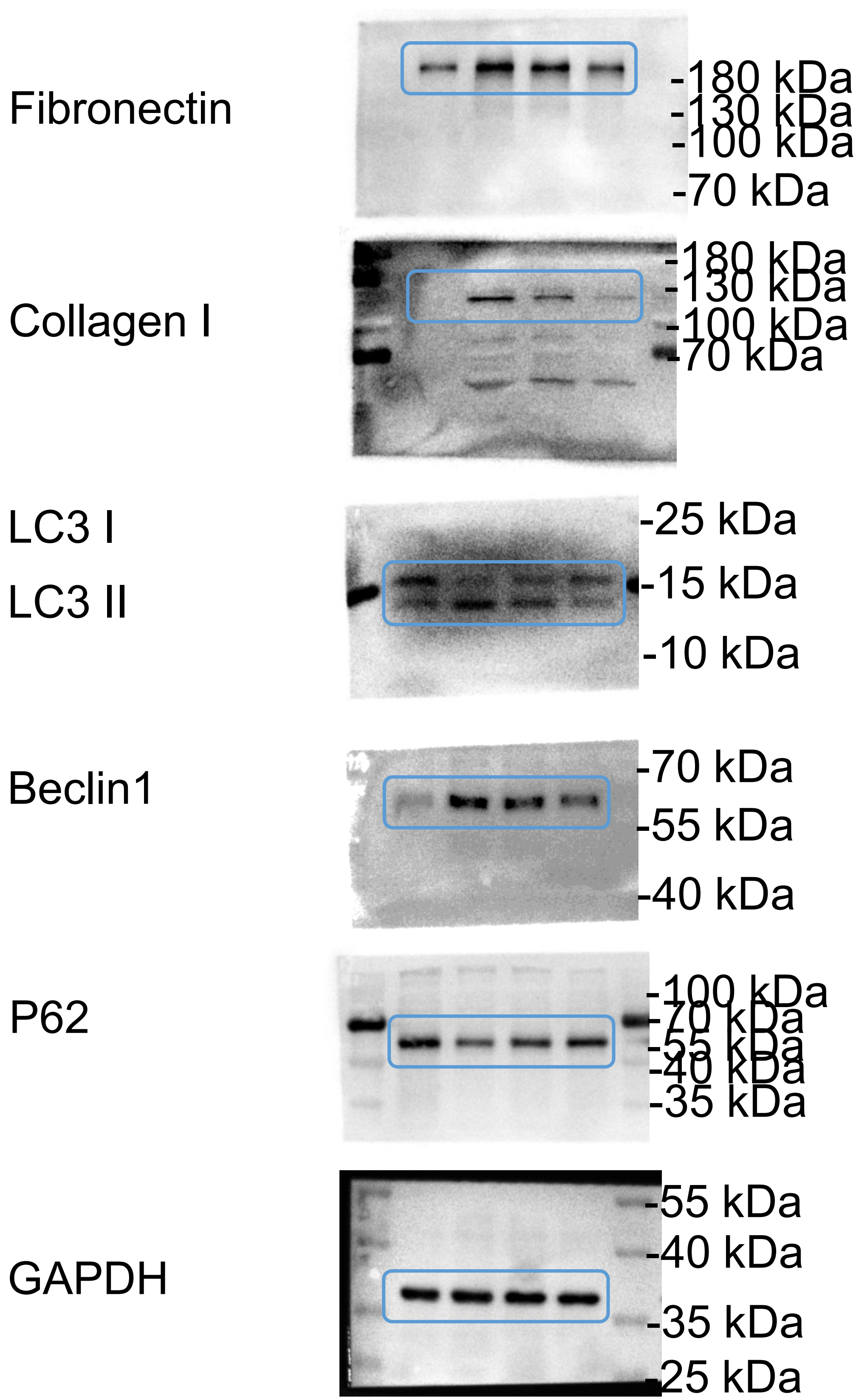

Full unedited gel for Figure 5F

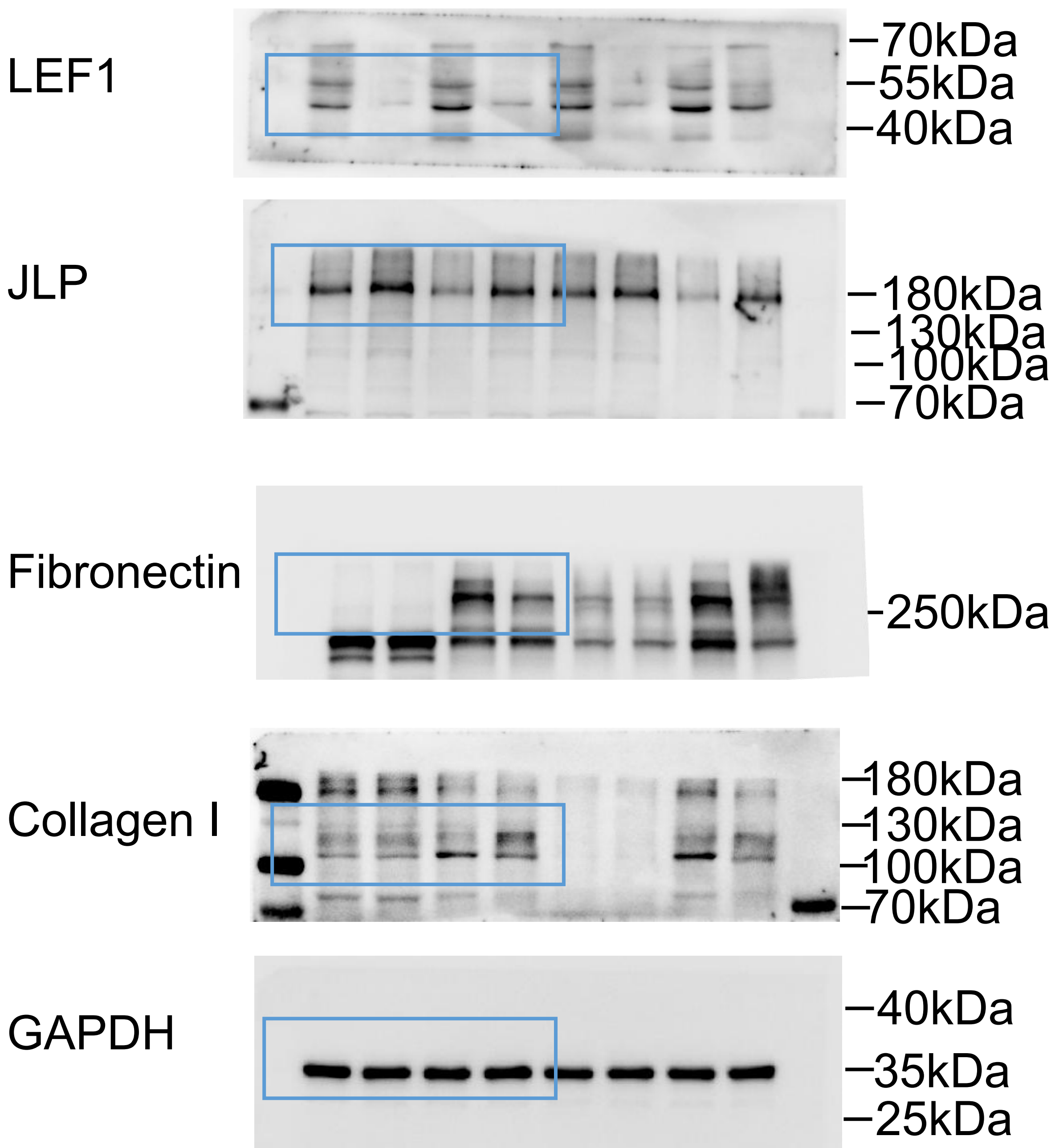

Full unedited gel for Figure 5M

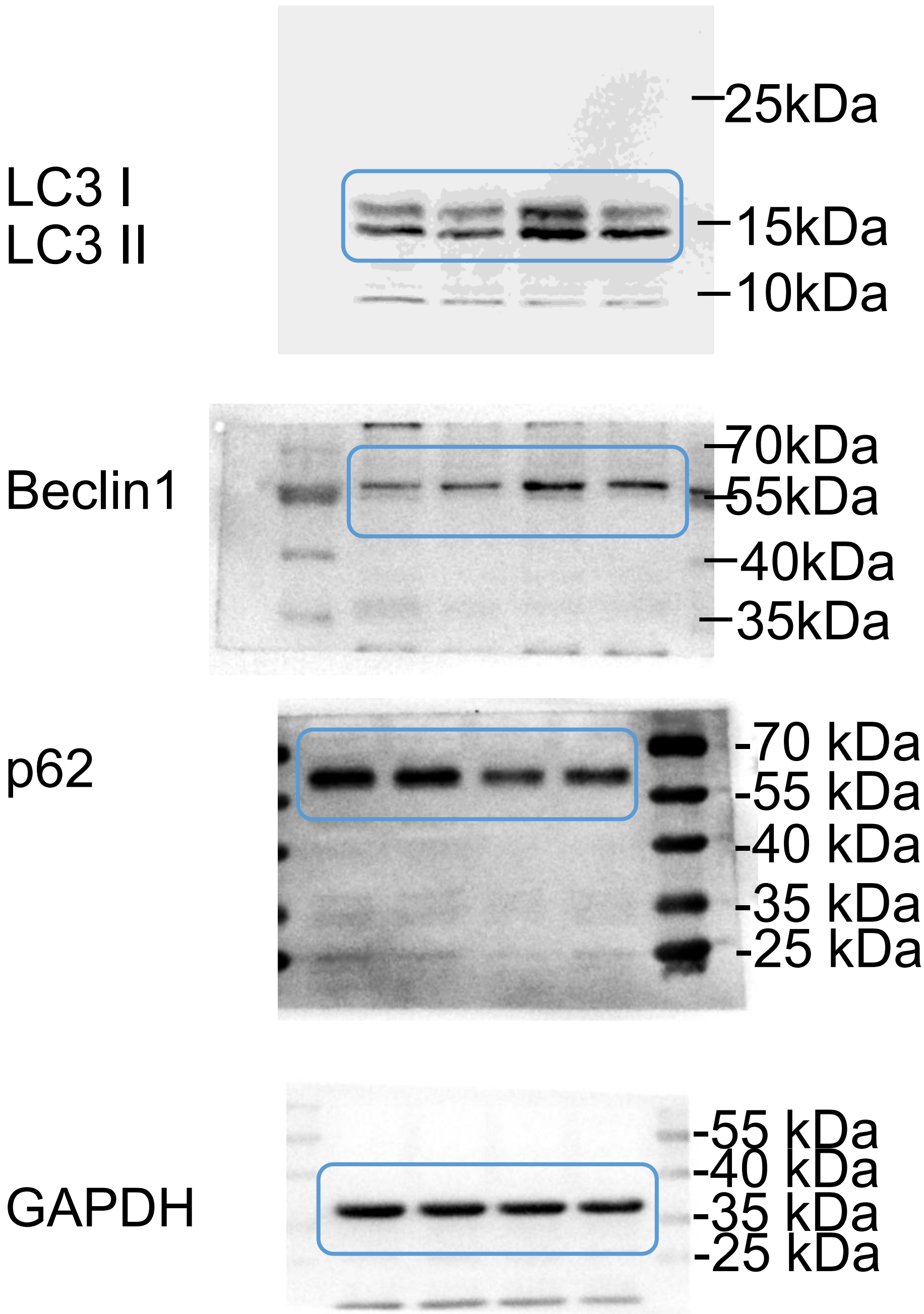

Full unedited gel for Figure 6D

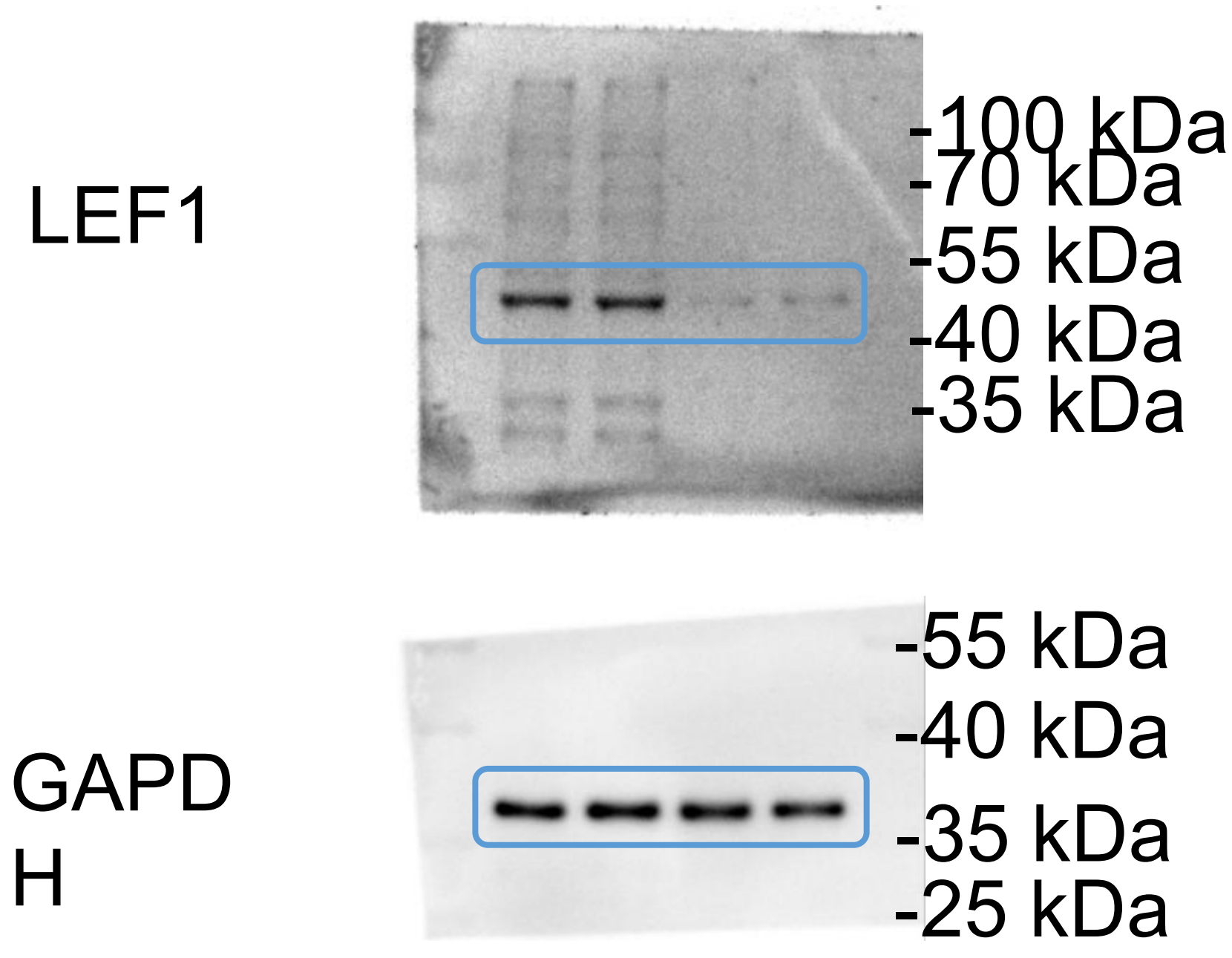

Full unedited gel for Figure 6J

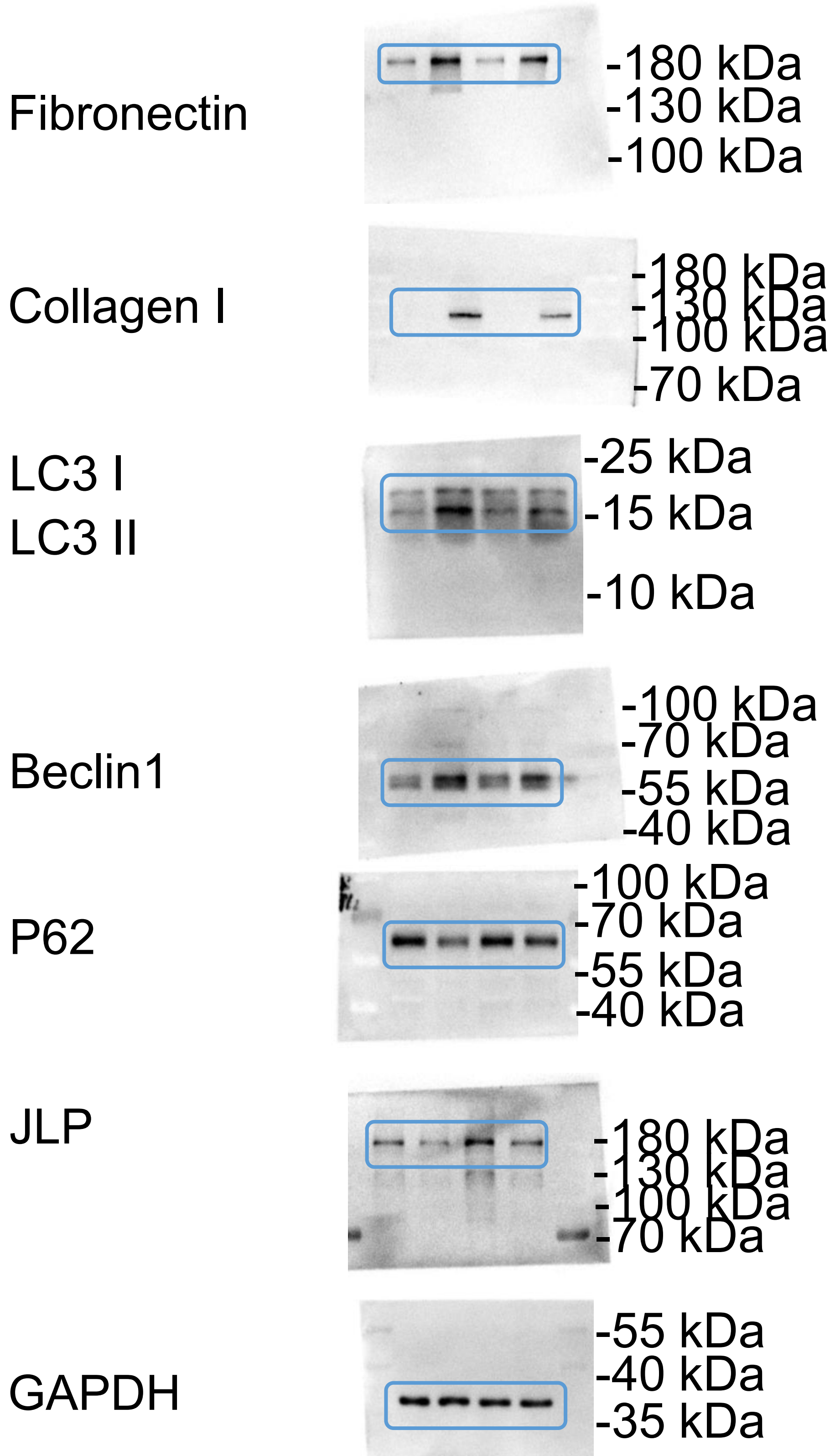

Full unedited gel for Figure 7A IP

β-catenin

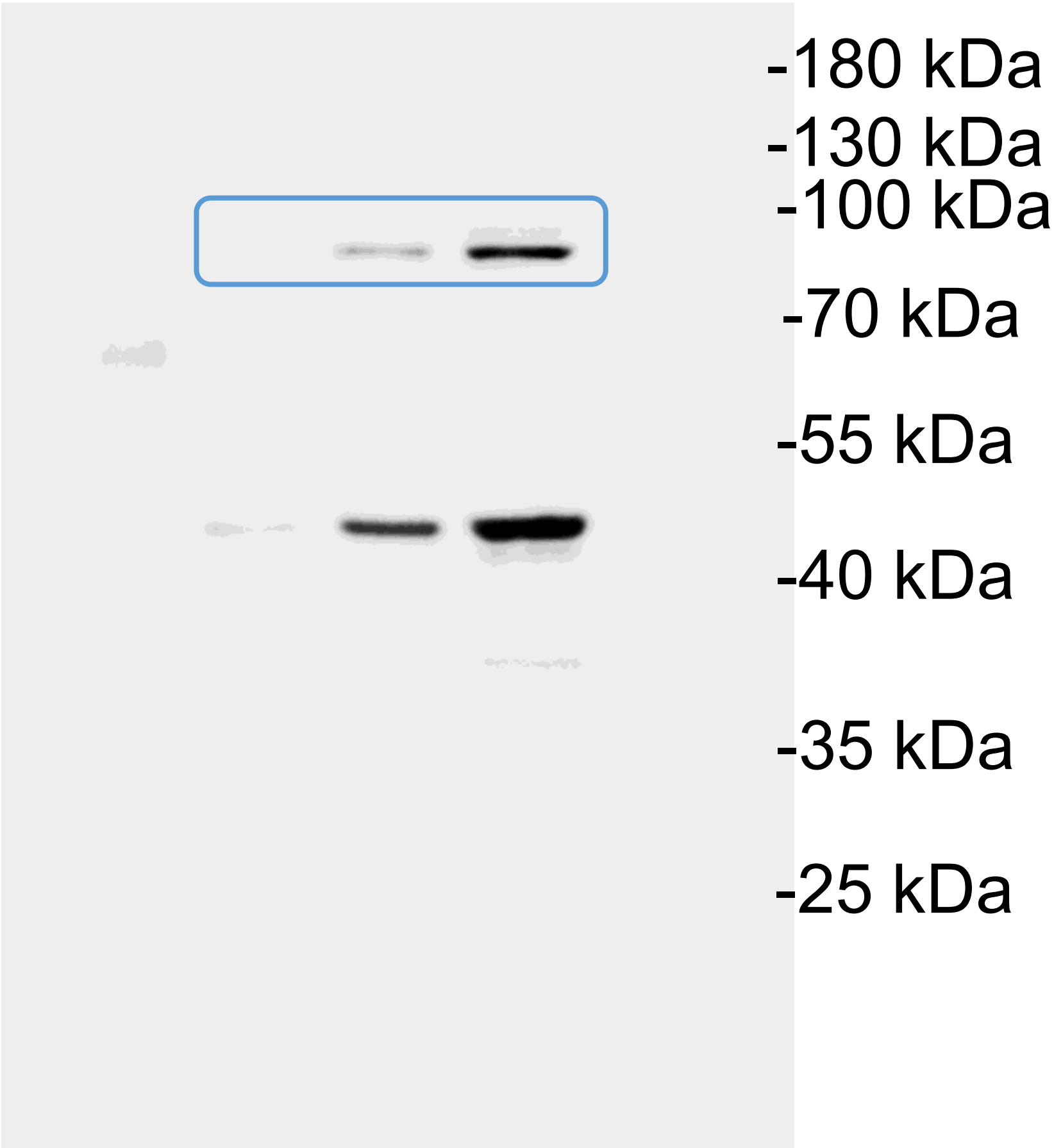

LEF1

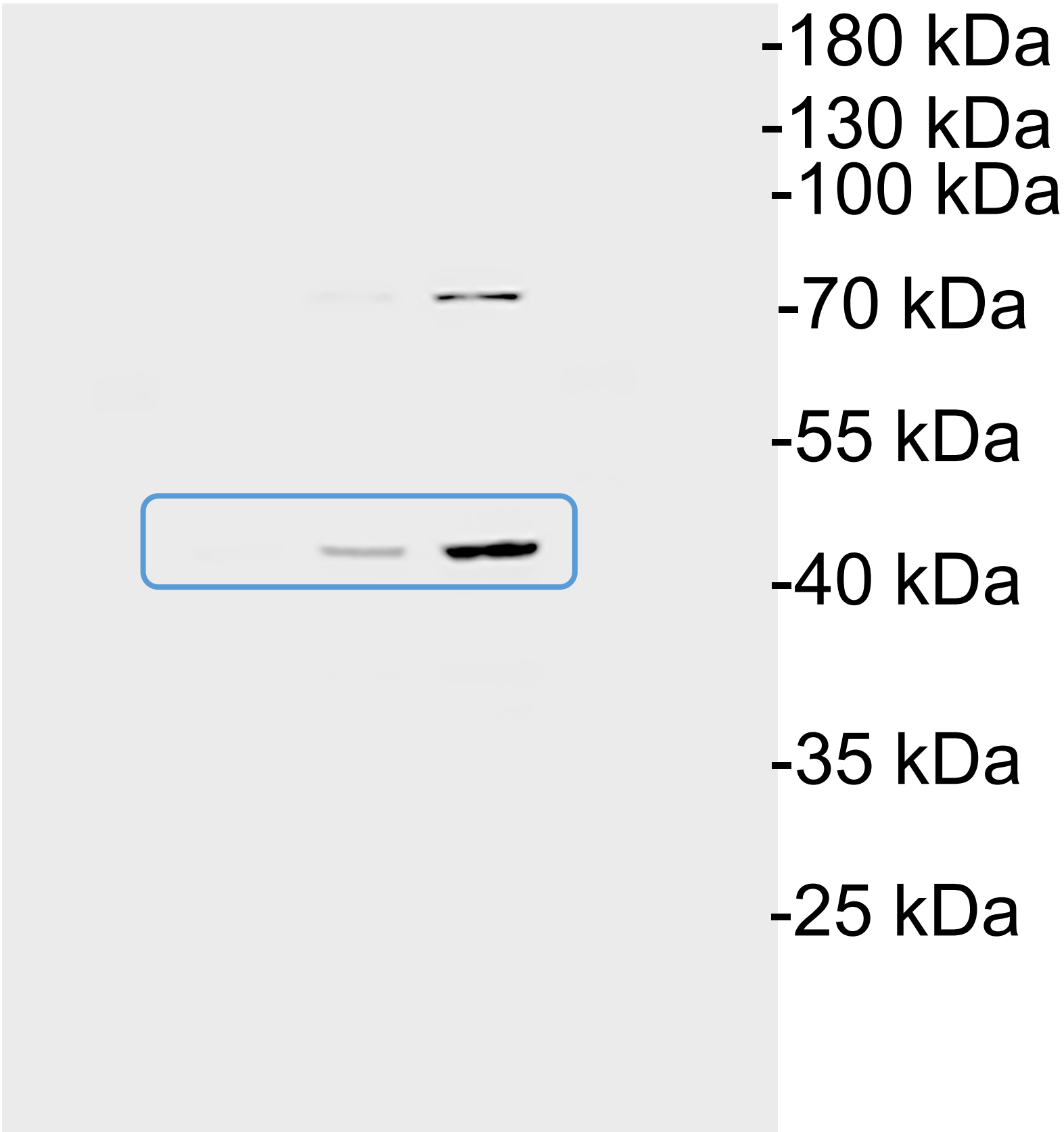

Full unedited gel for Figure 7A Input

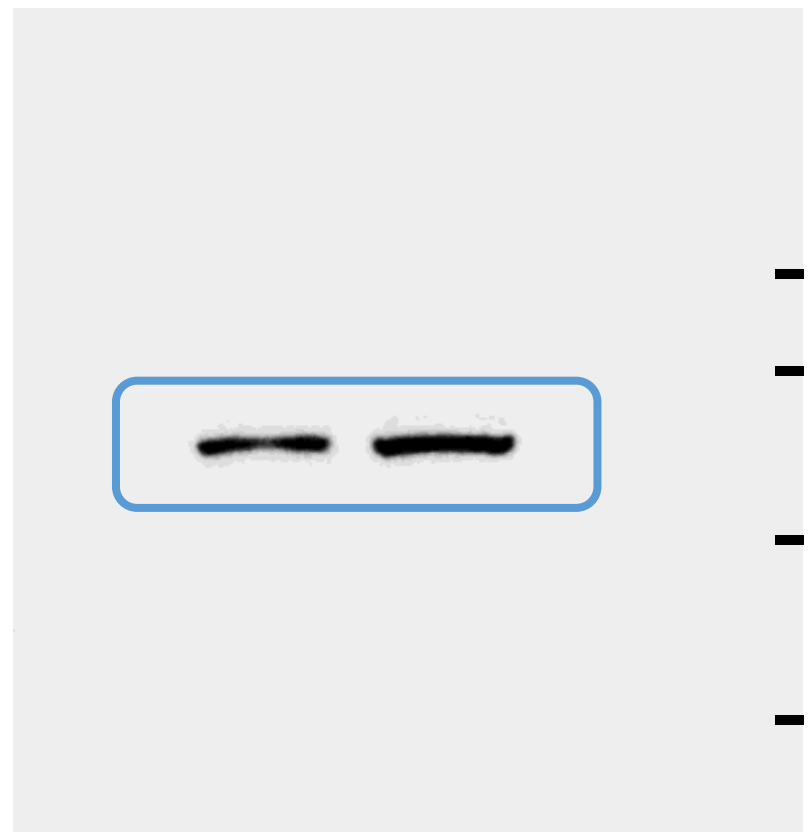

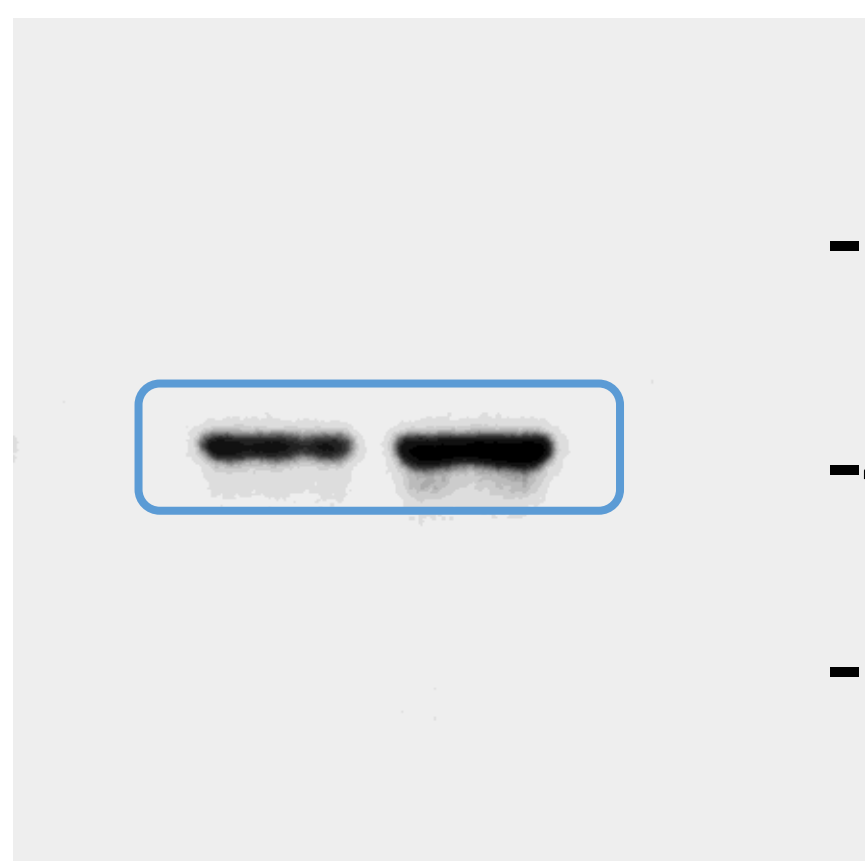

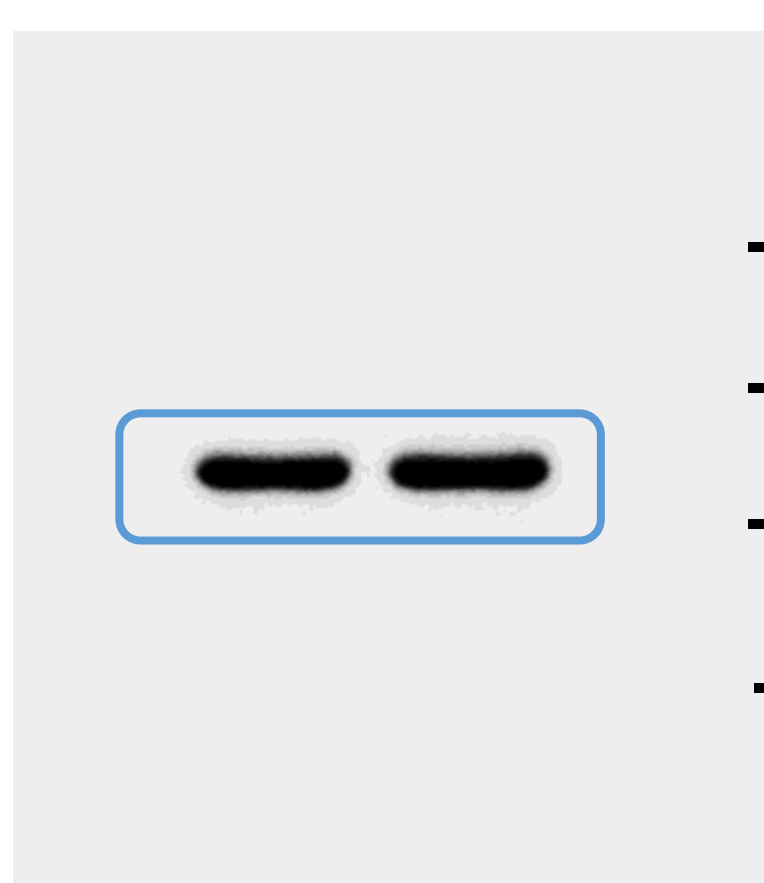

Full unedited gel for Figure 7D IP

β-catenin

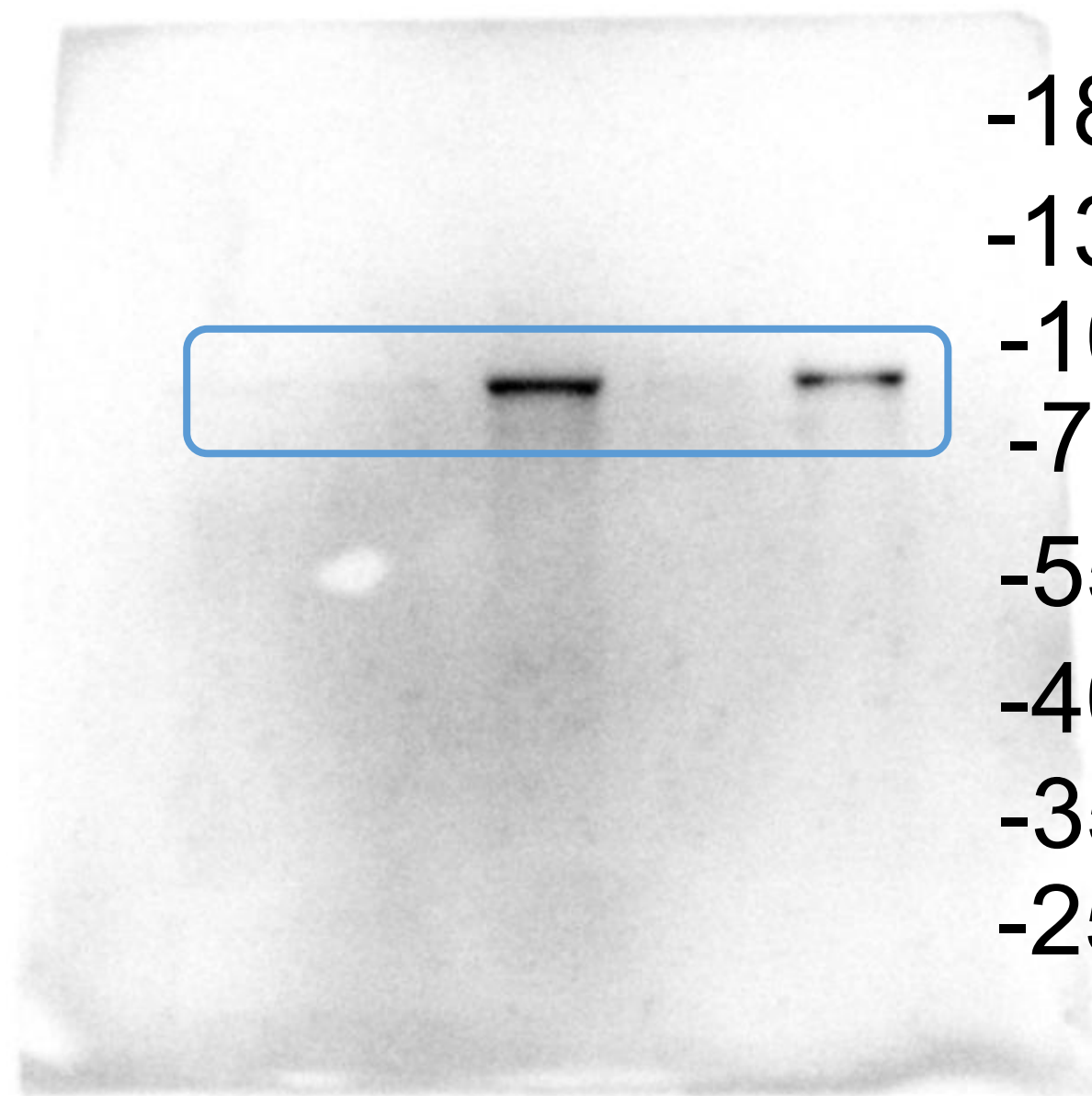

LEF1

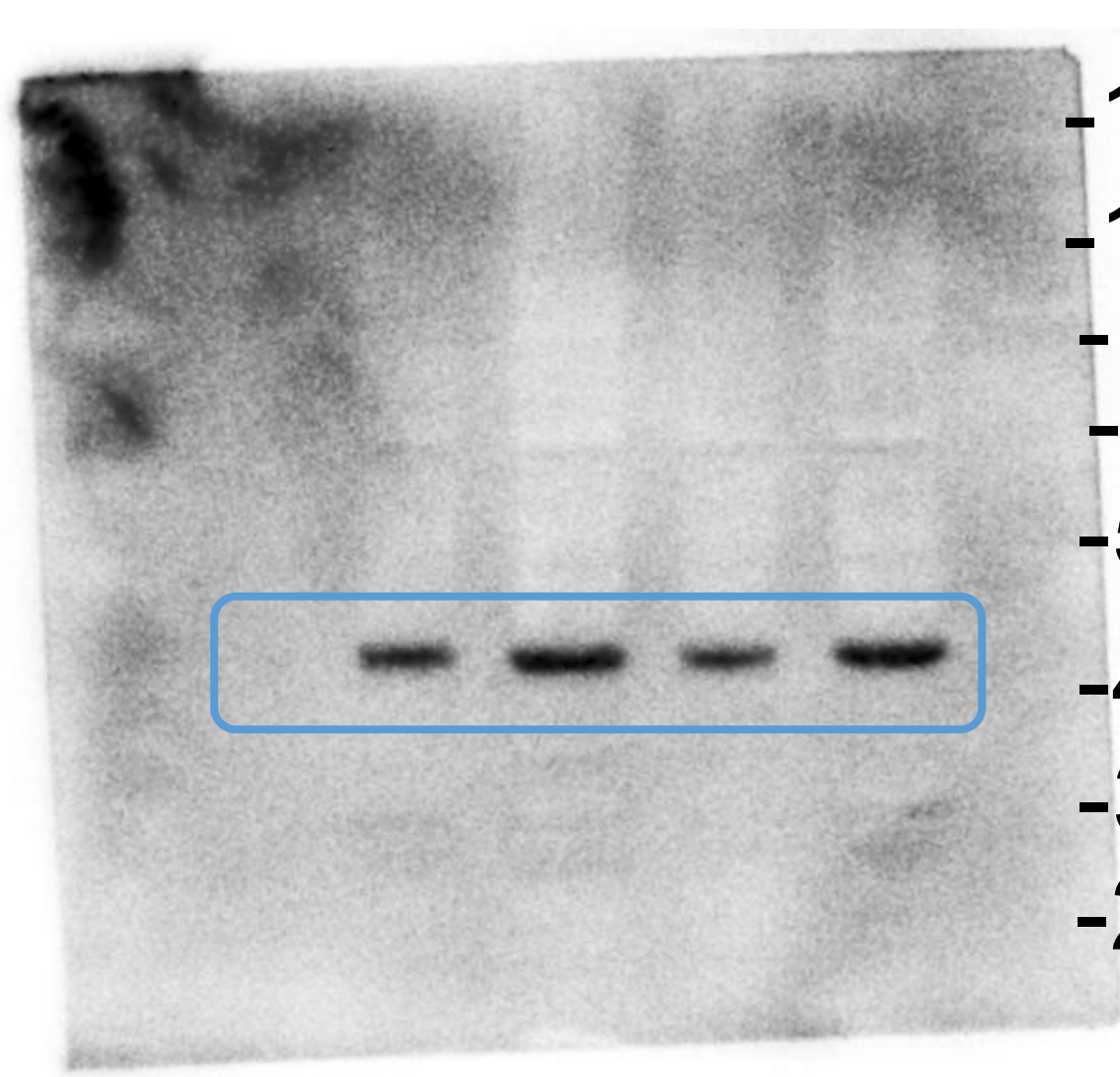

Full unedited gel for Figure 7D Input

β-catenin

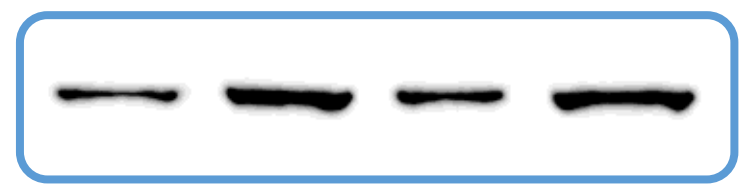

-180 kDa
-130 kDa
-100 kDa
-70 kDa

LEF1

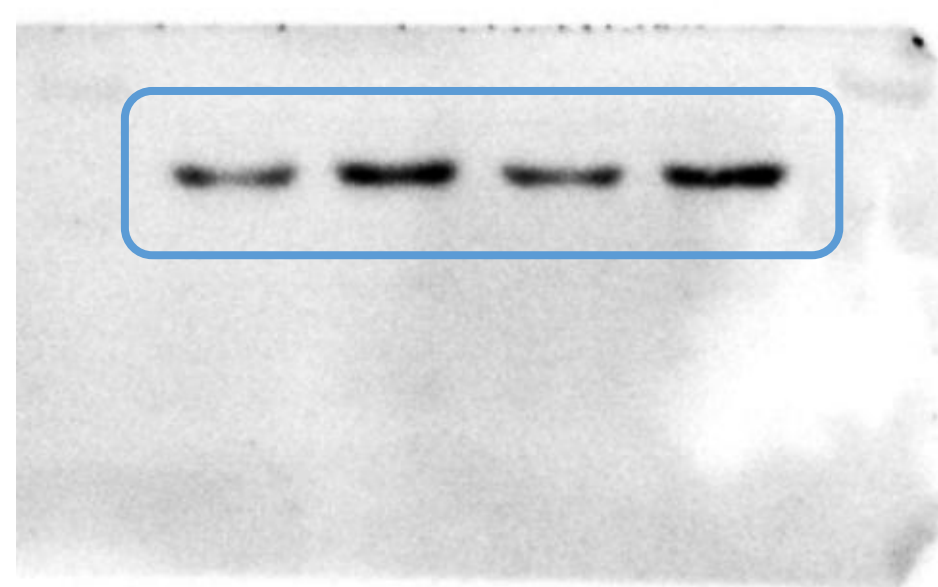

-55 kDa
-40 kDa
-35 kDa
-25 kDa

GAPDH

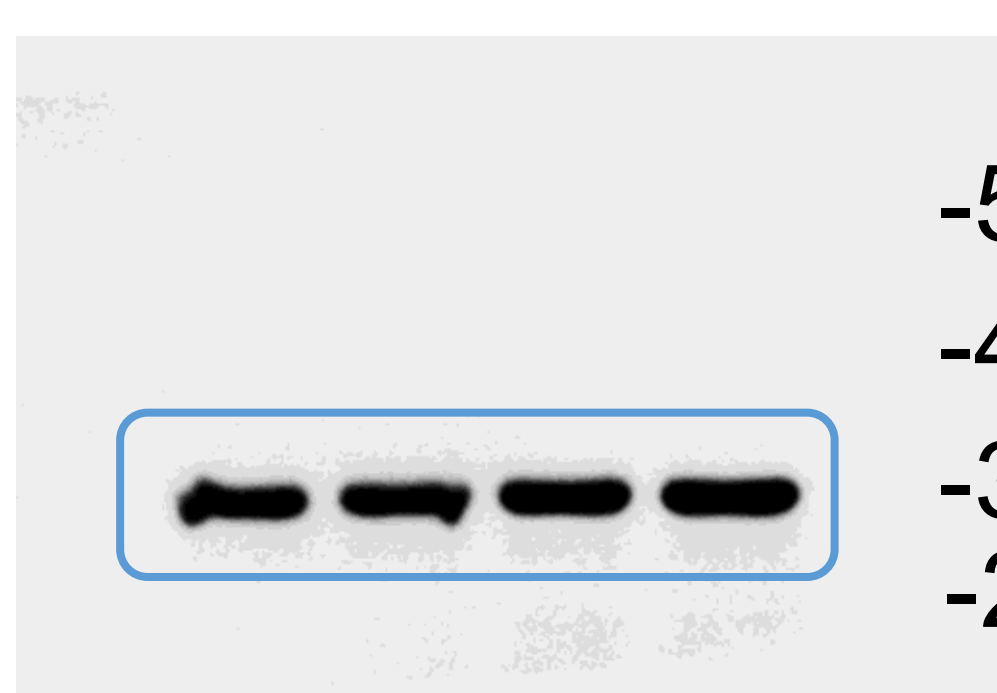

-55 kDa
-40 kDa
-35 kDa
-25 kDa

Full unedited gel for Figure 7E

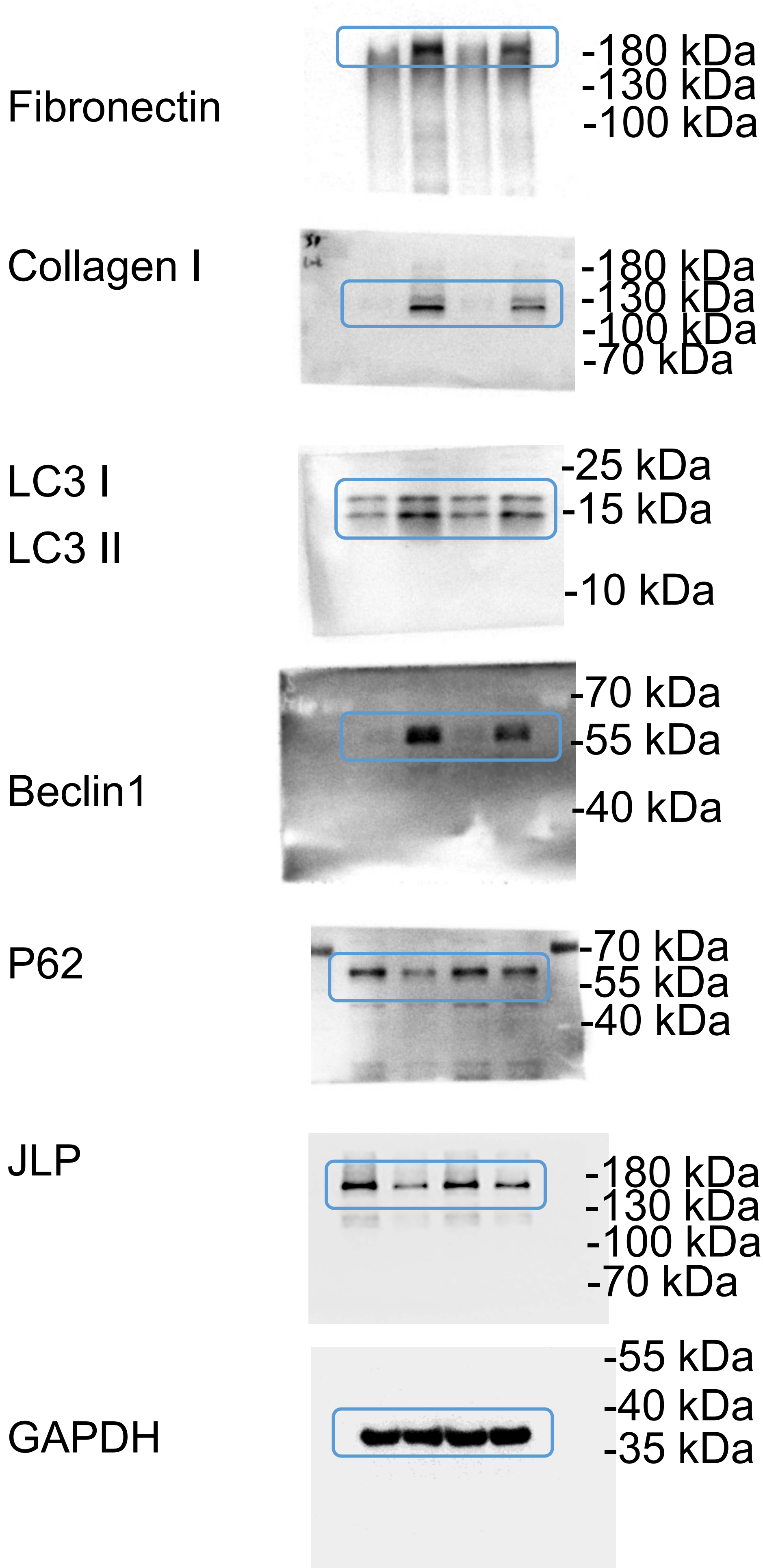

Full unedited gel for Figure 7F

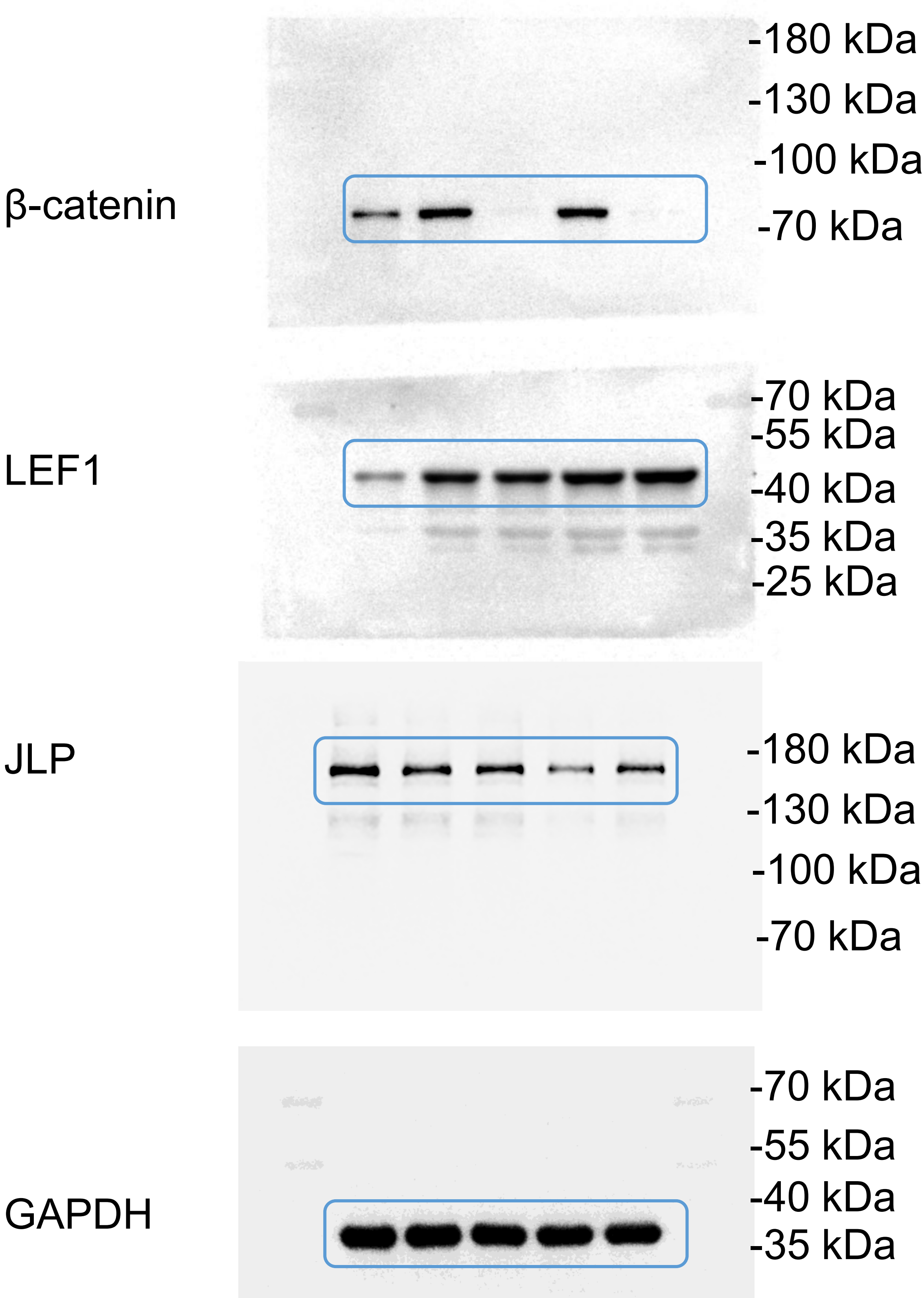

Full unedited gel for Figure 7G

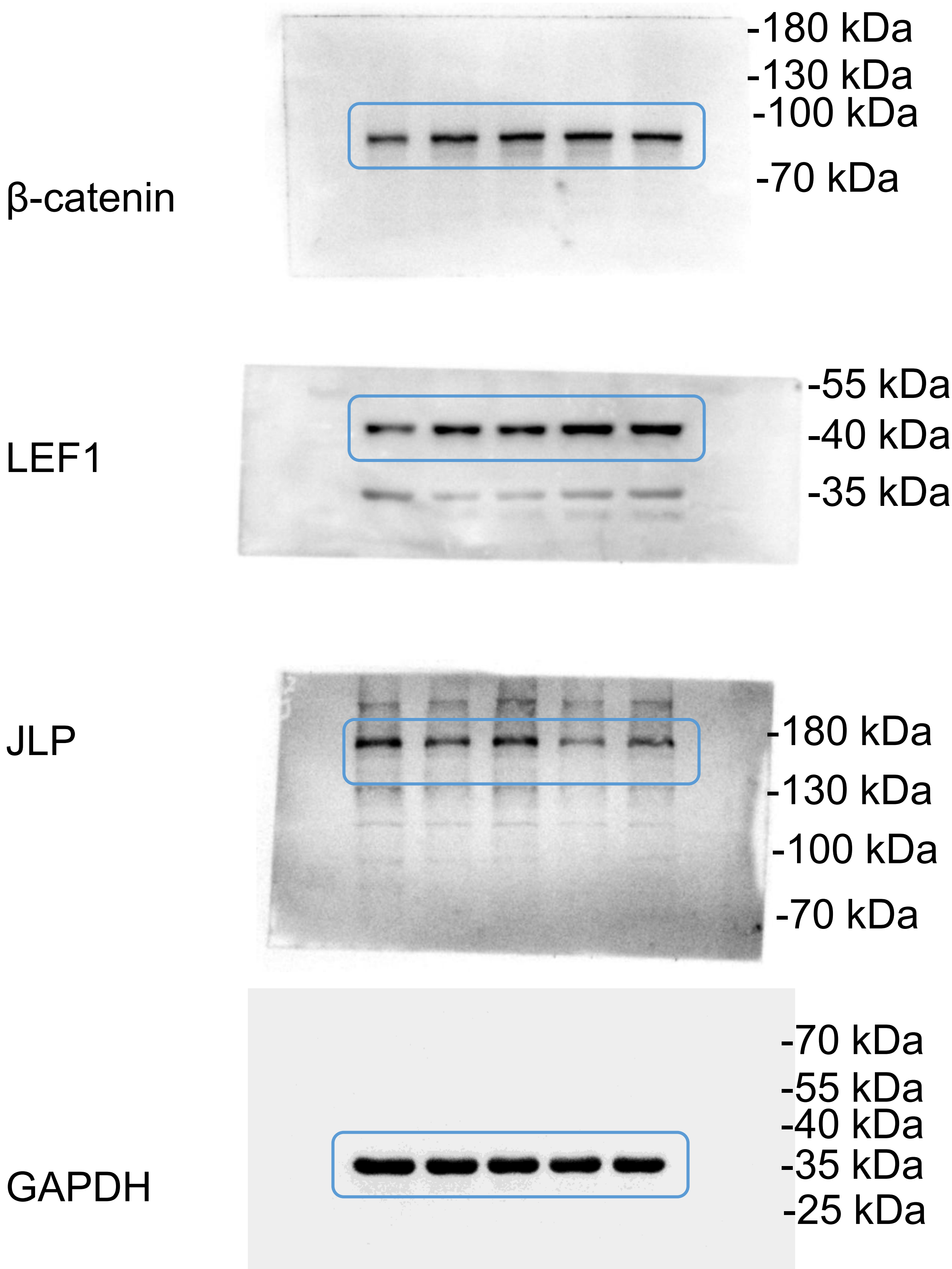

Full unedited gel for Figure 8G

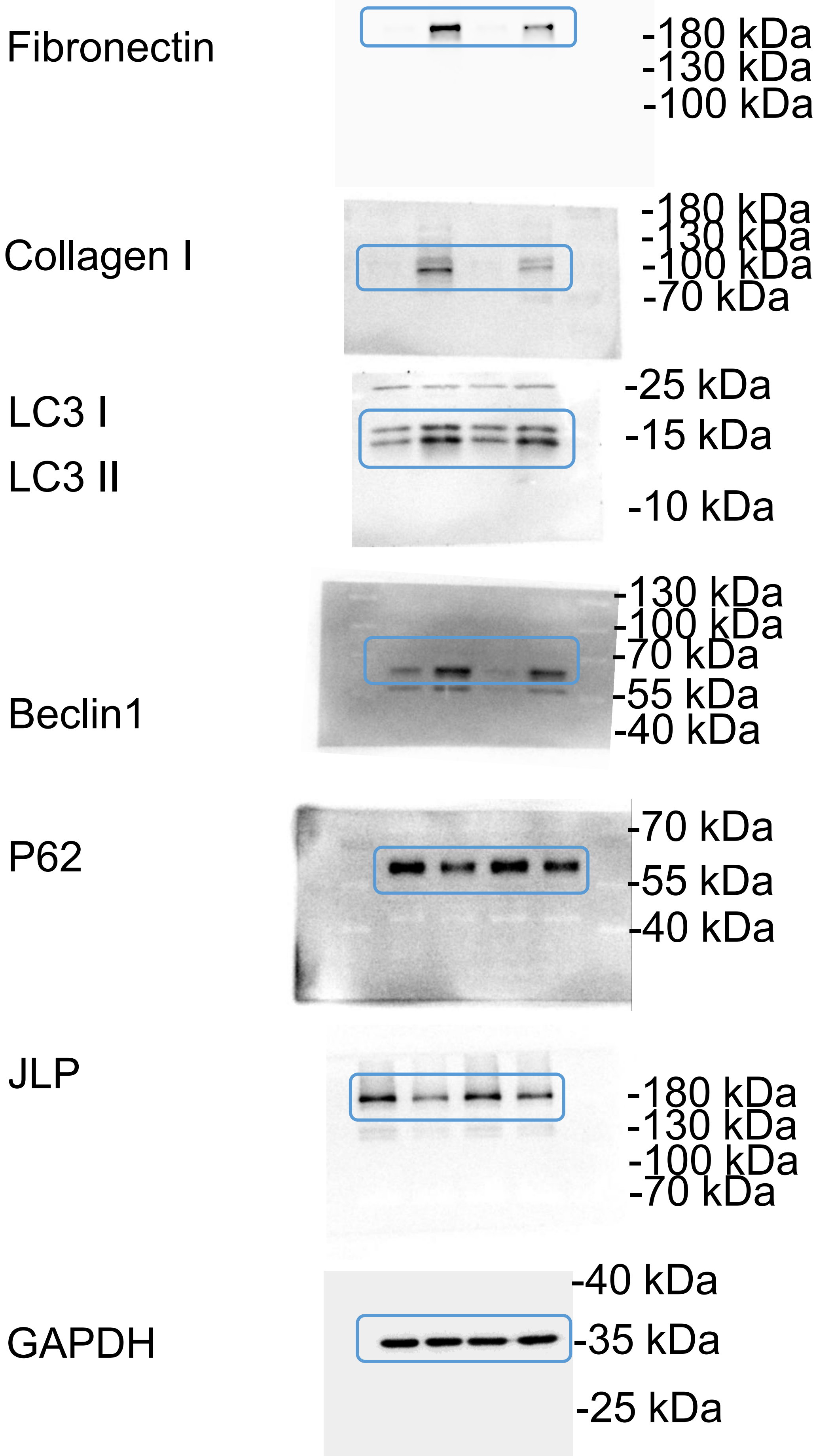

Full unedited gel for Figure S1B

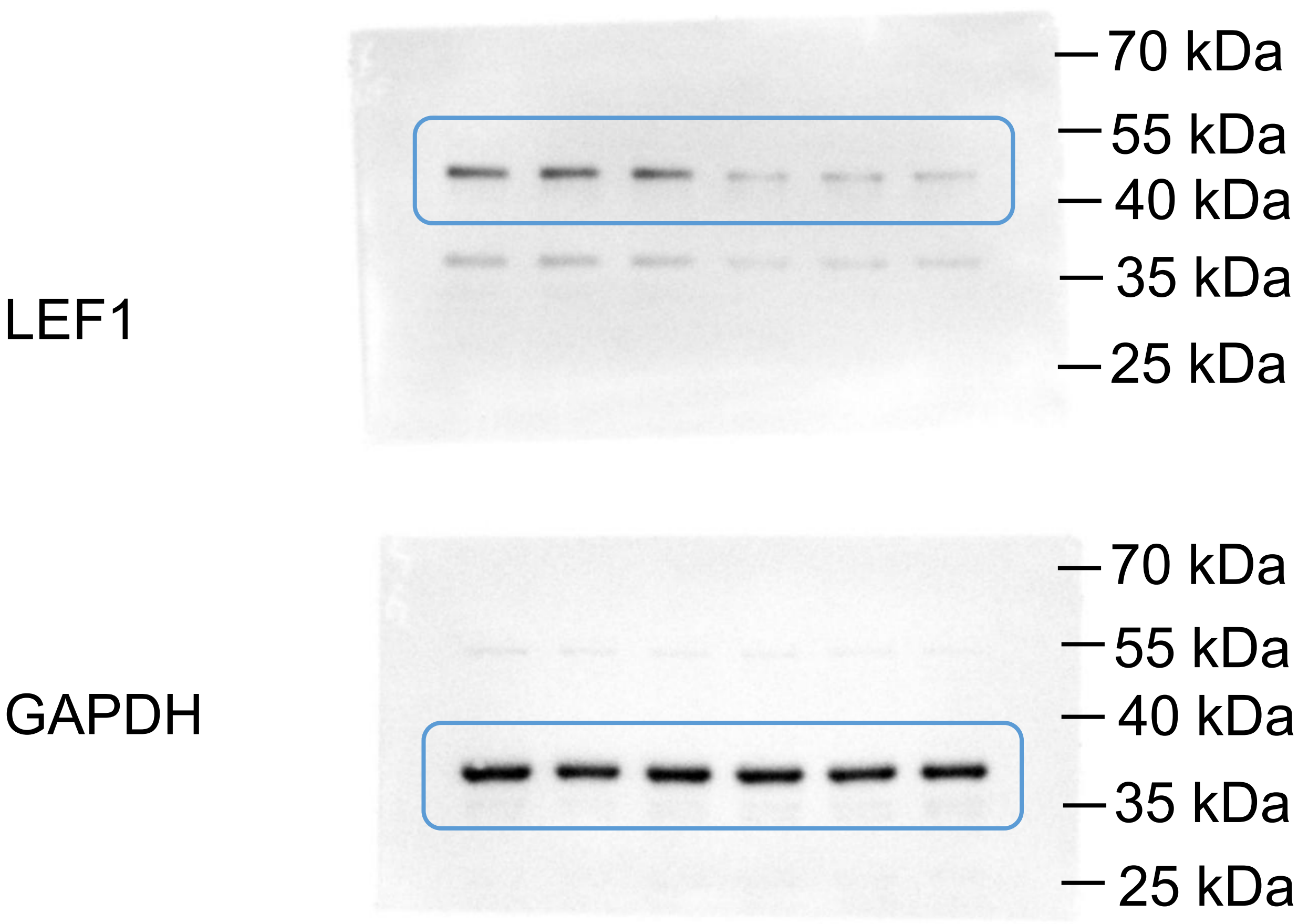

Full unedited gel for Figure S1C

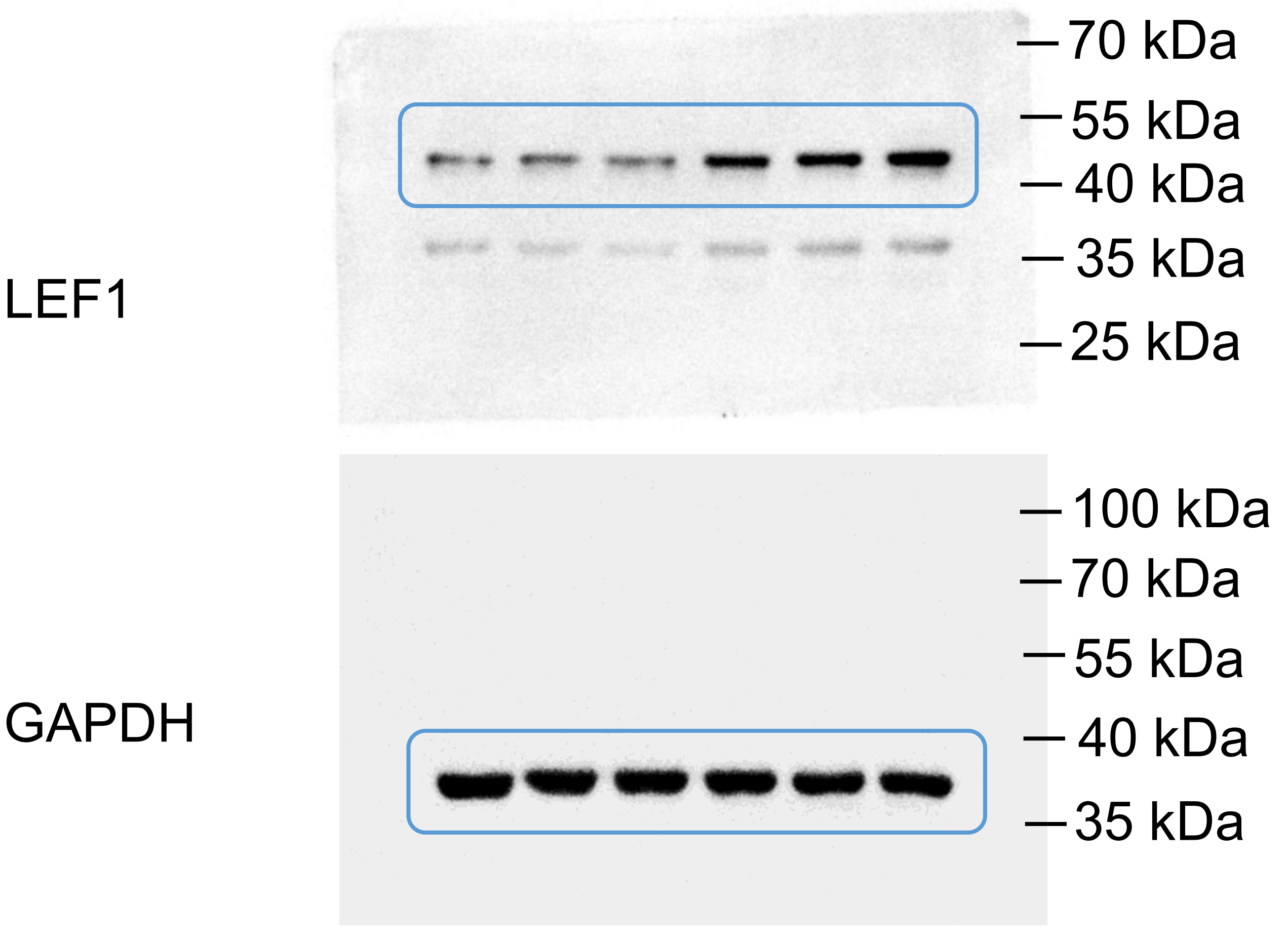

Full unedited gel for Figure 3B

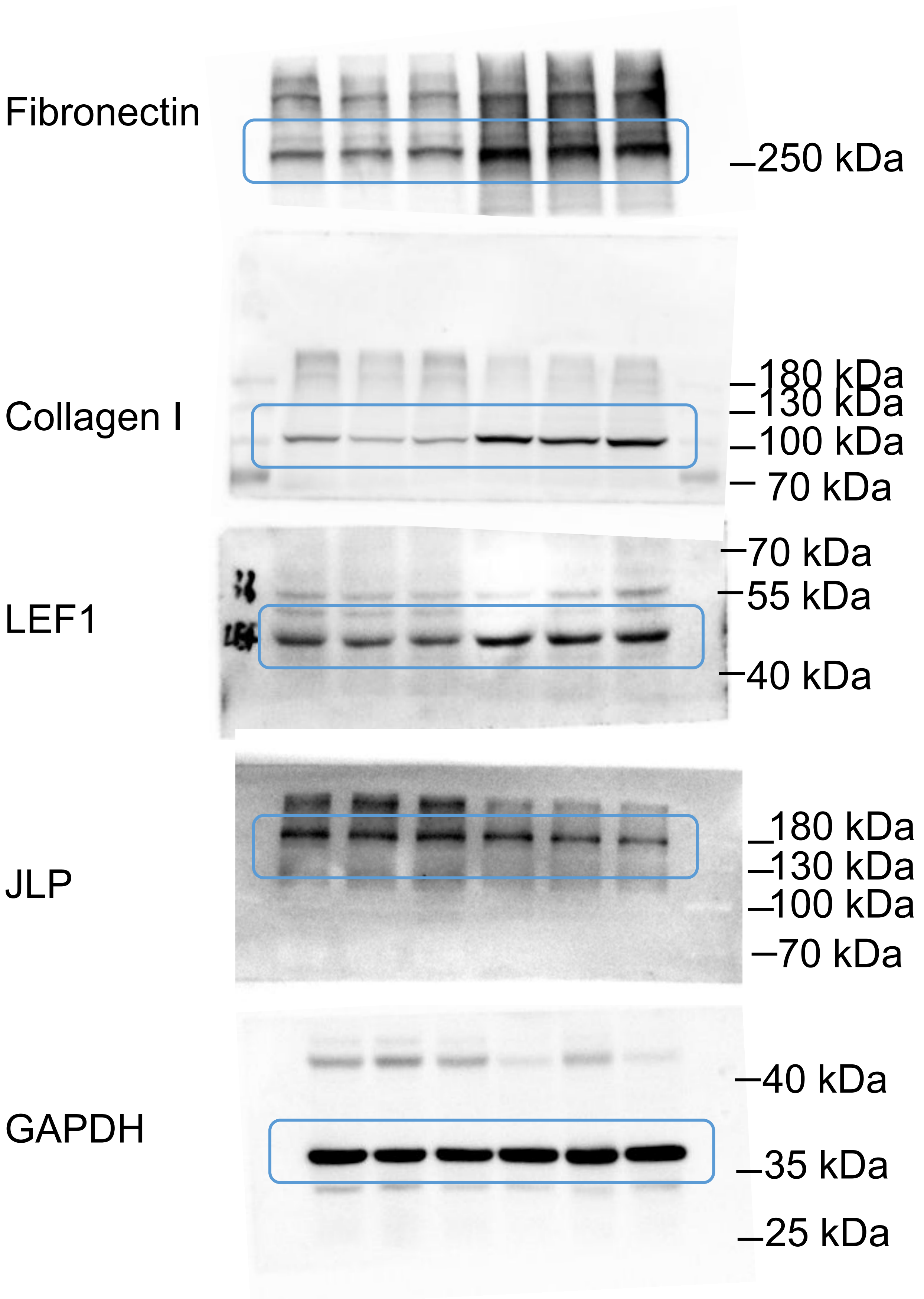

Full unedited gel for Figure S4B

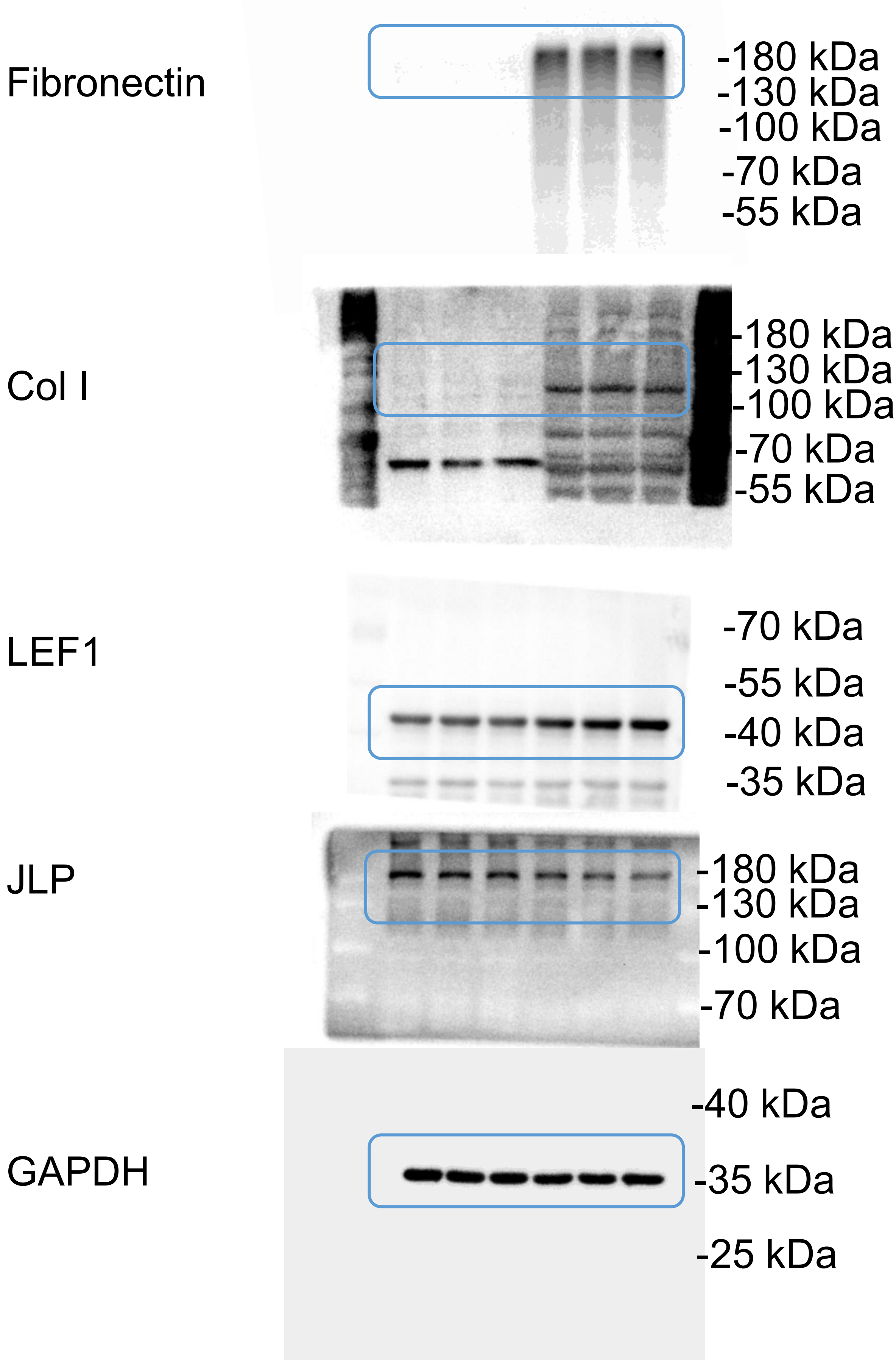

Full unedited gel for Figure S4G

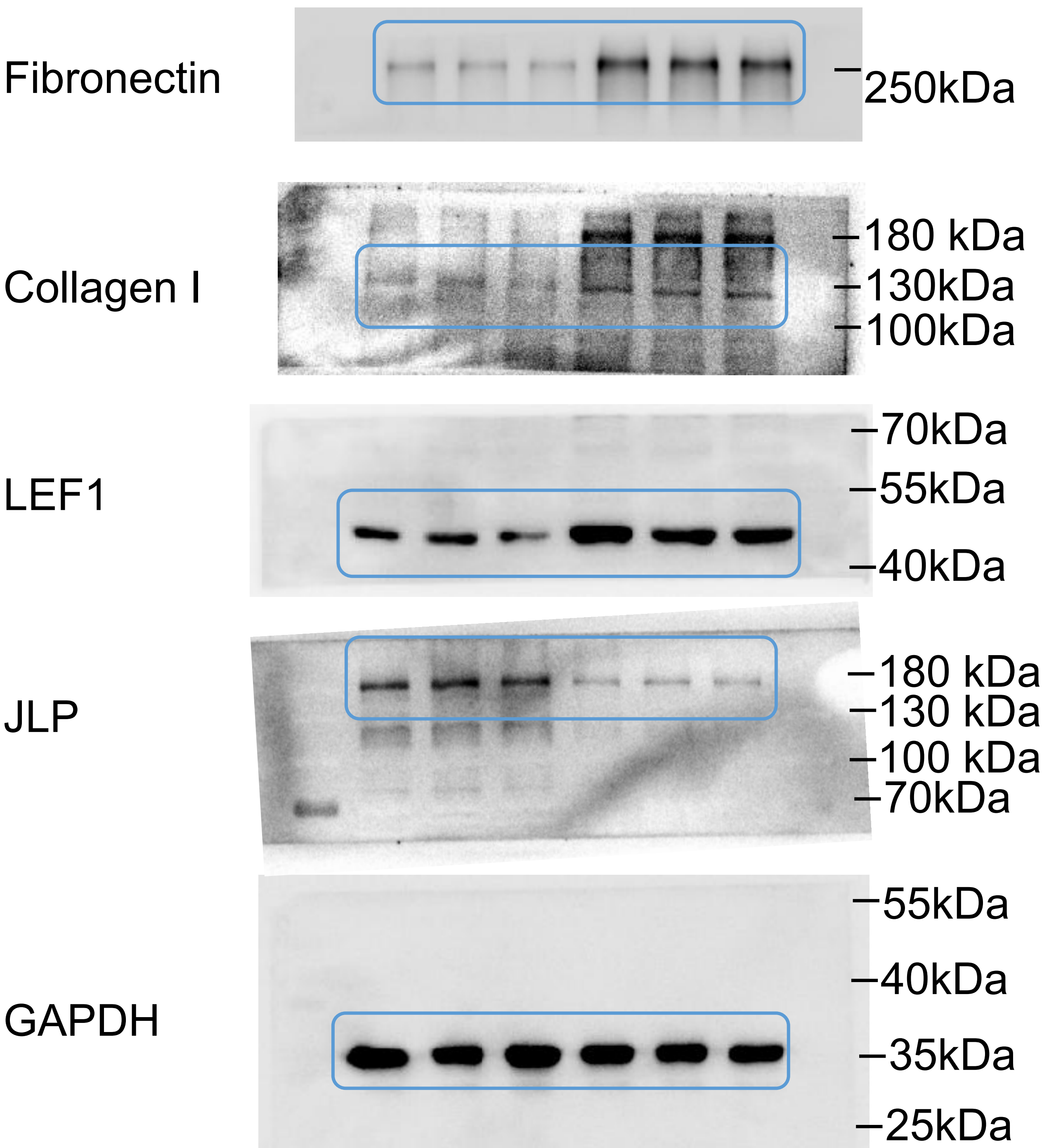

Full unedited gel for Figure S5A

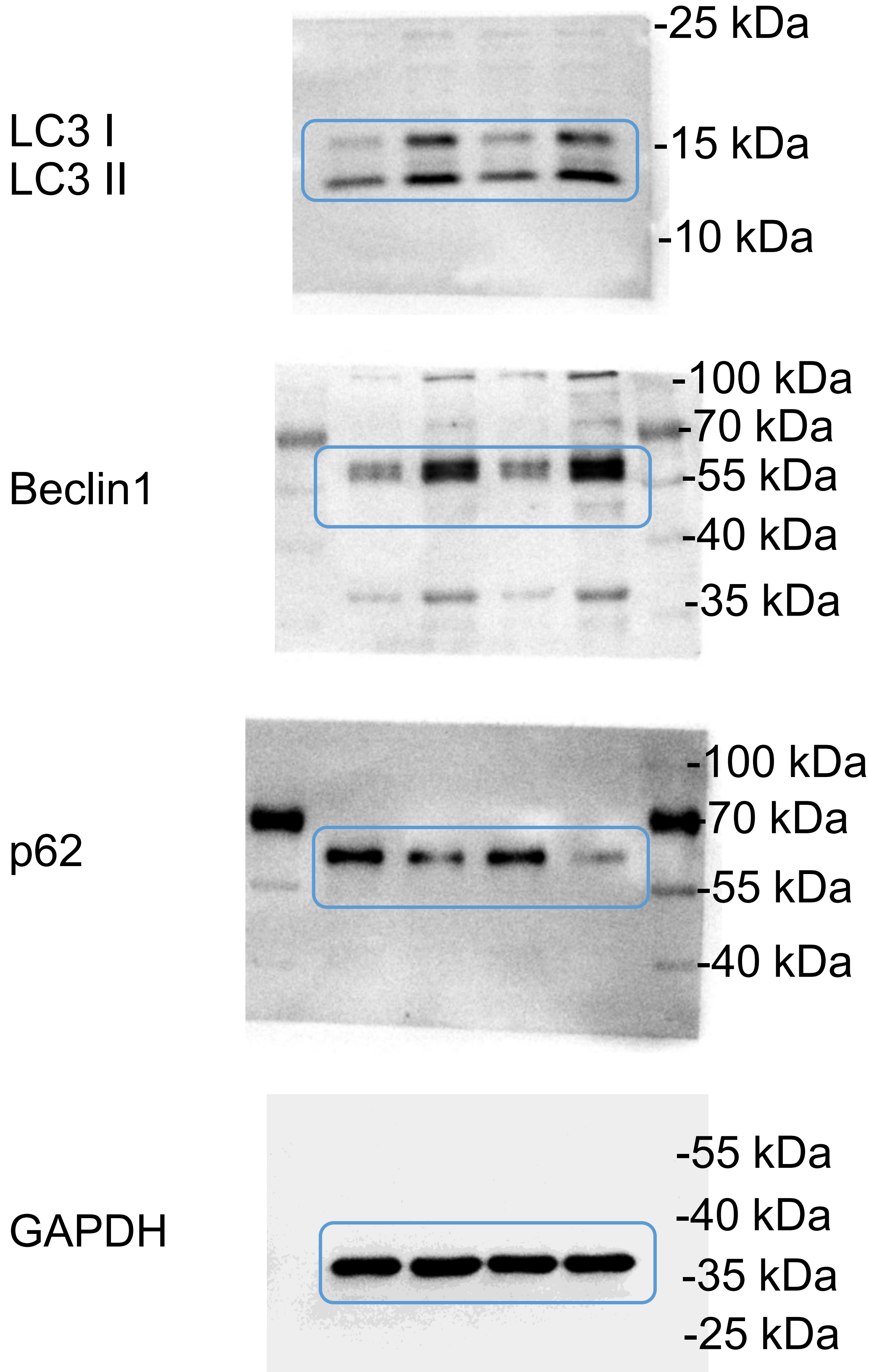

Full unedited gel for Figure S5B

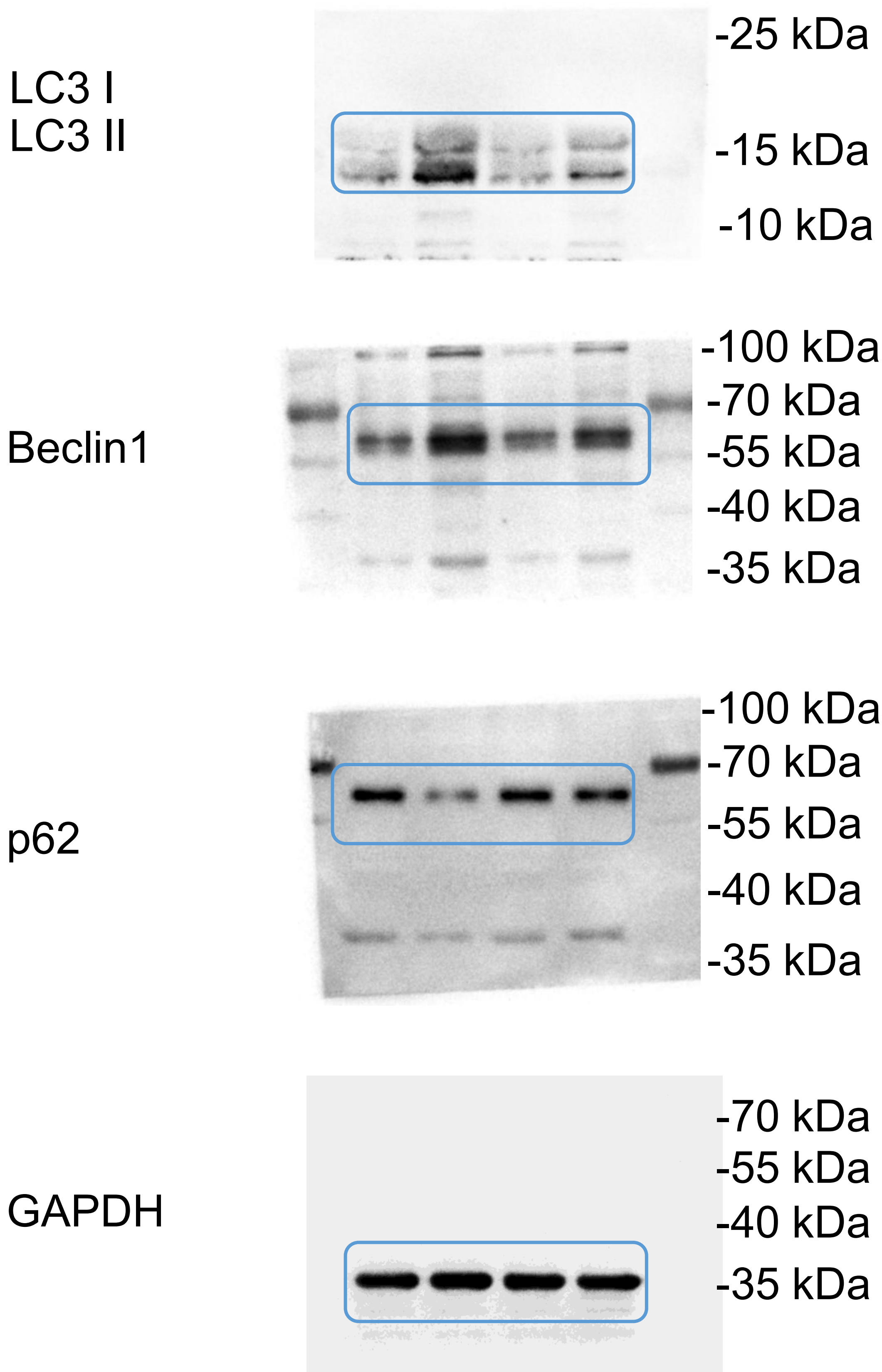

Full unedited gel for Figure S7D

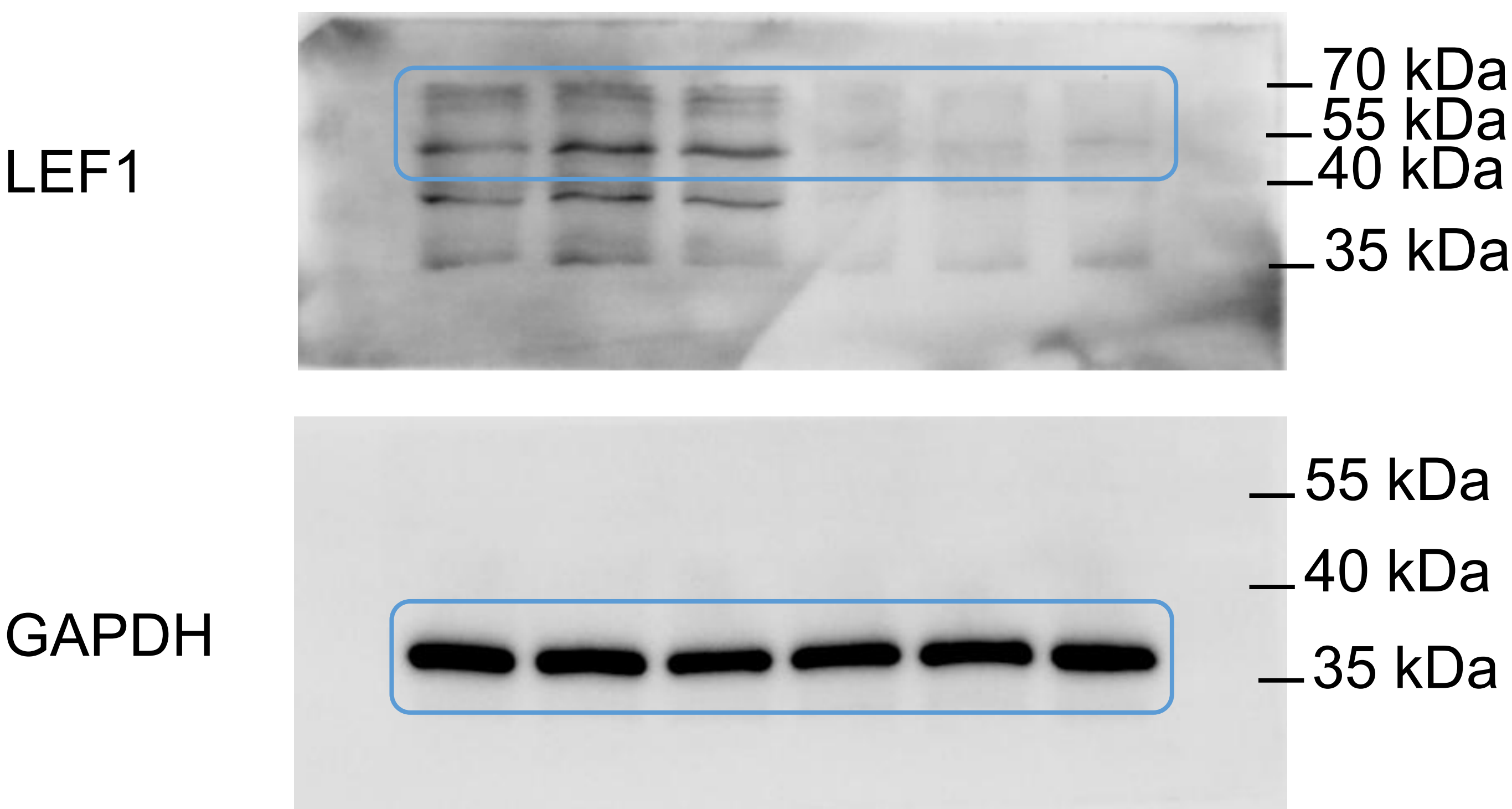

Full unedited gel for Figure S8E

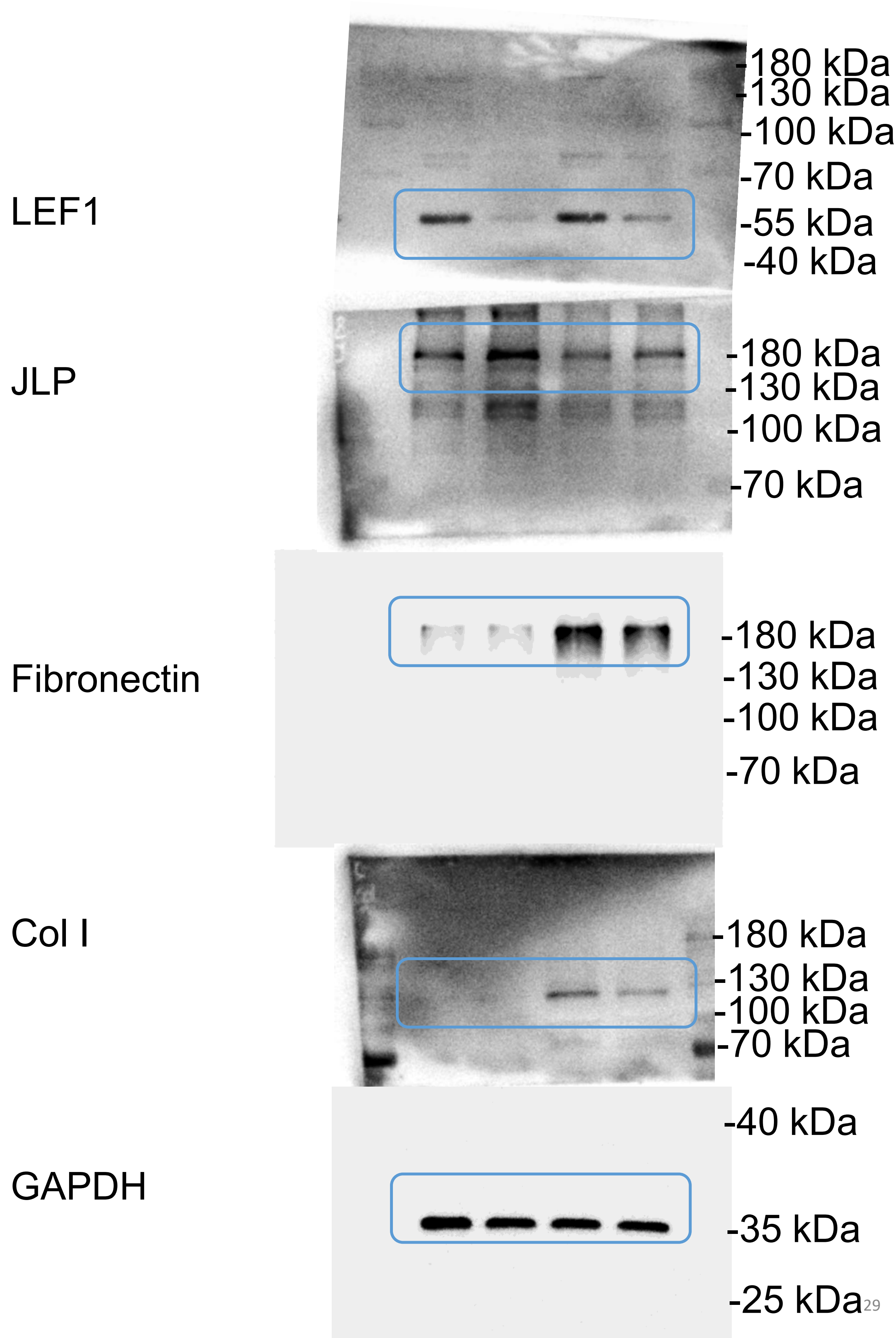

Full unedited gel for Figure S8I

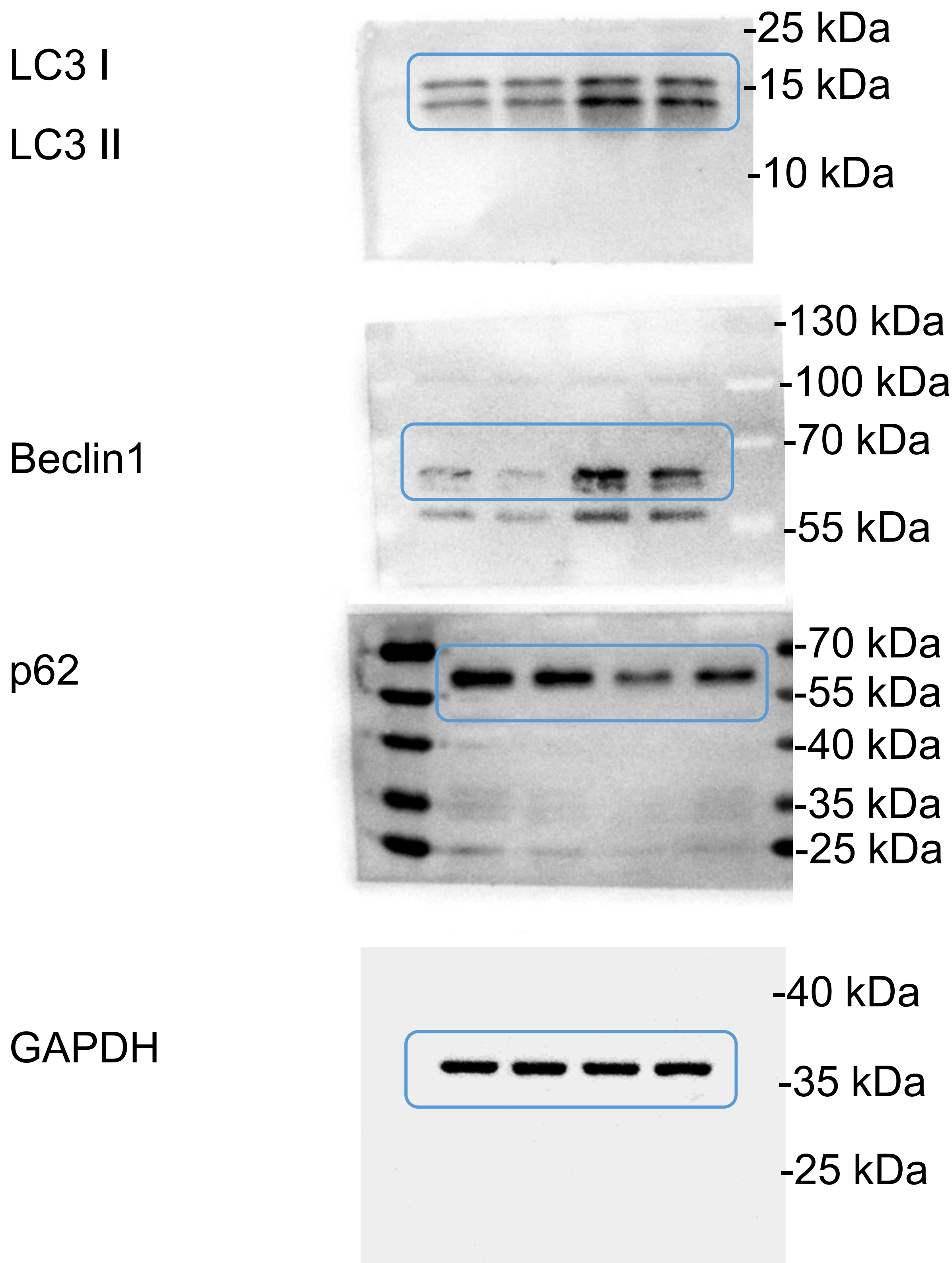

Full unedited gel for Figure S9A

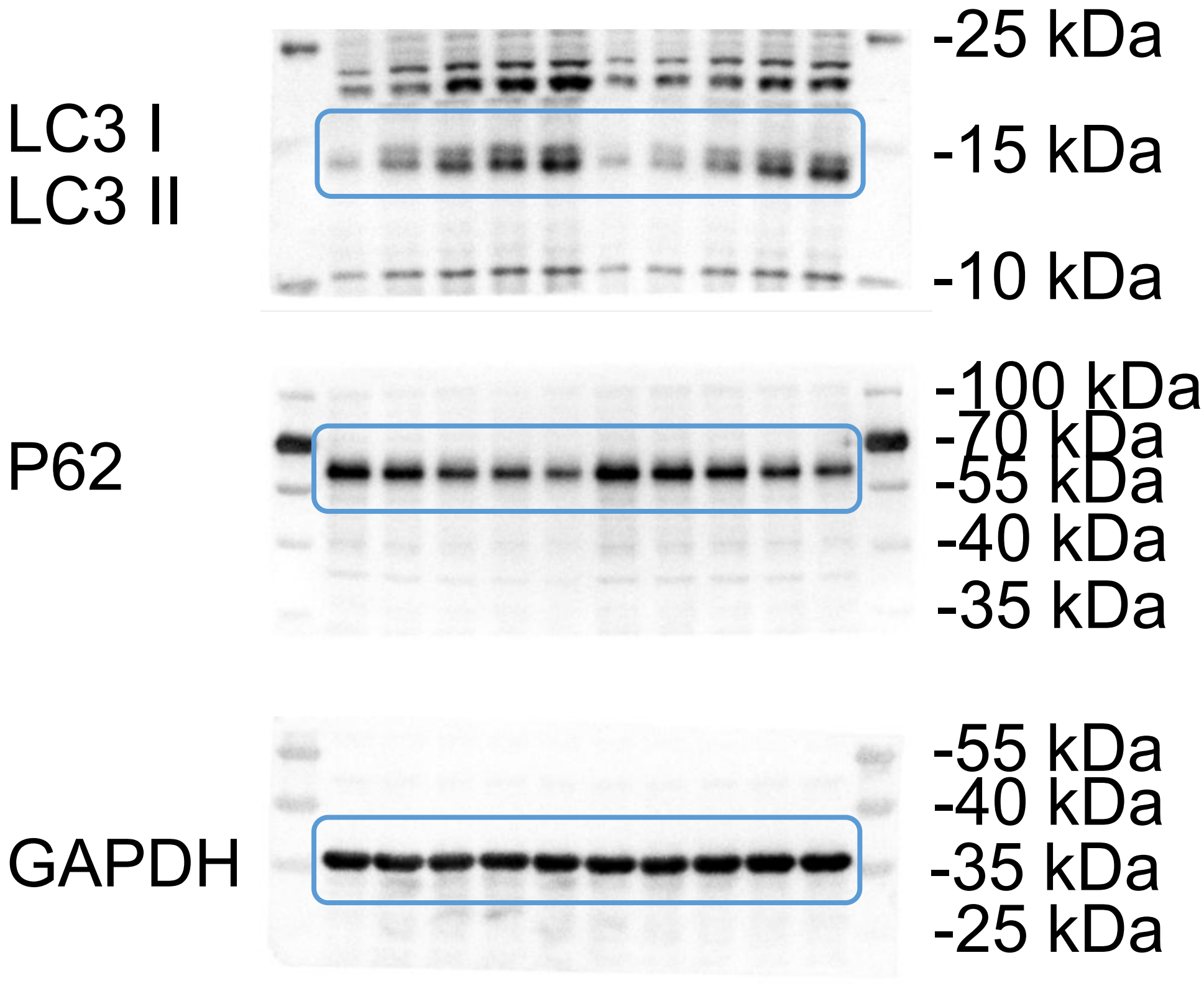

Full unedited gel for Figure S9C

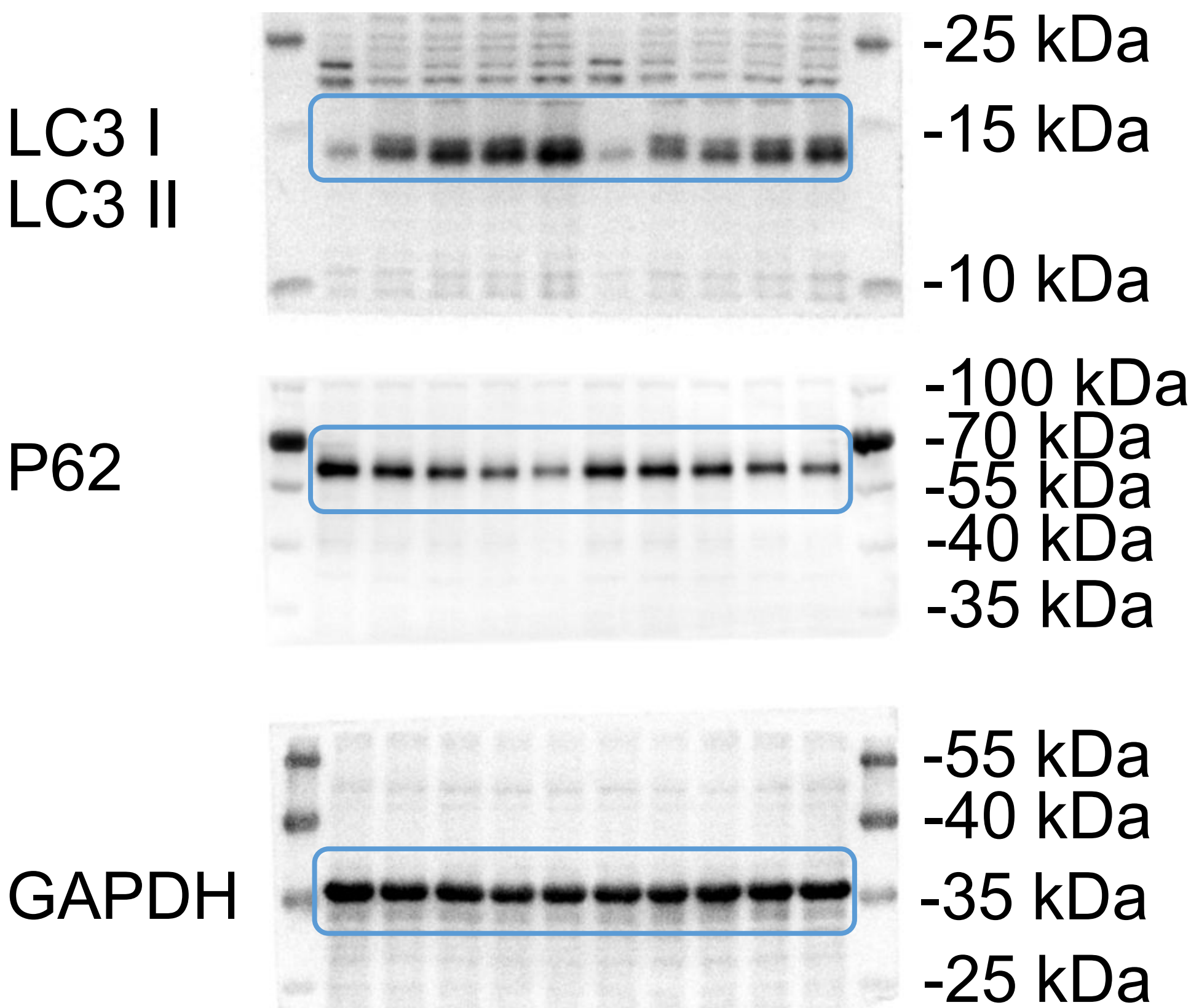

Full unedited gel for Figure S10E

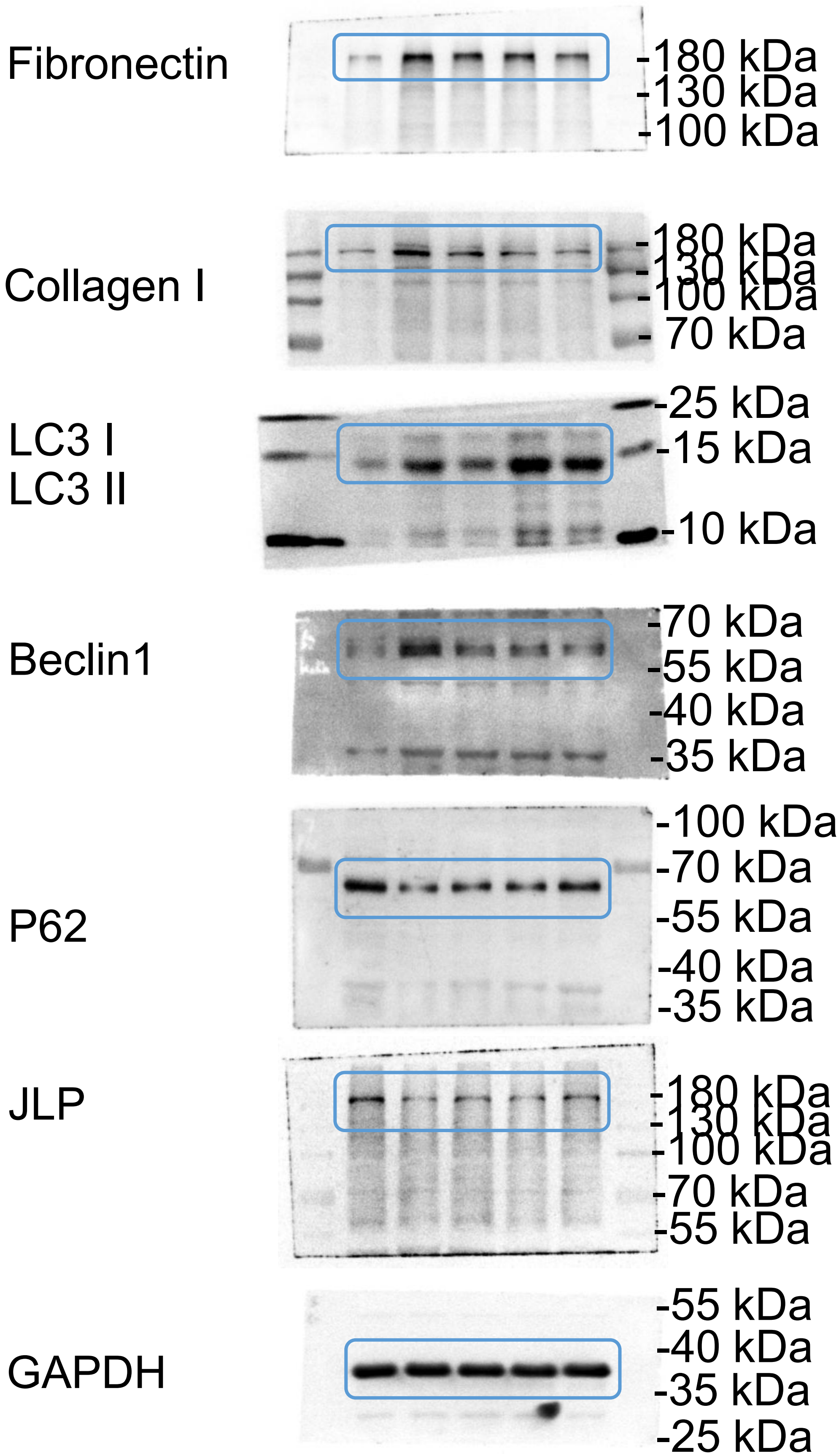

Full unedited gel for Figure S11G

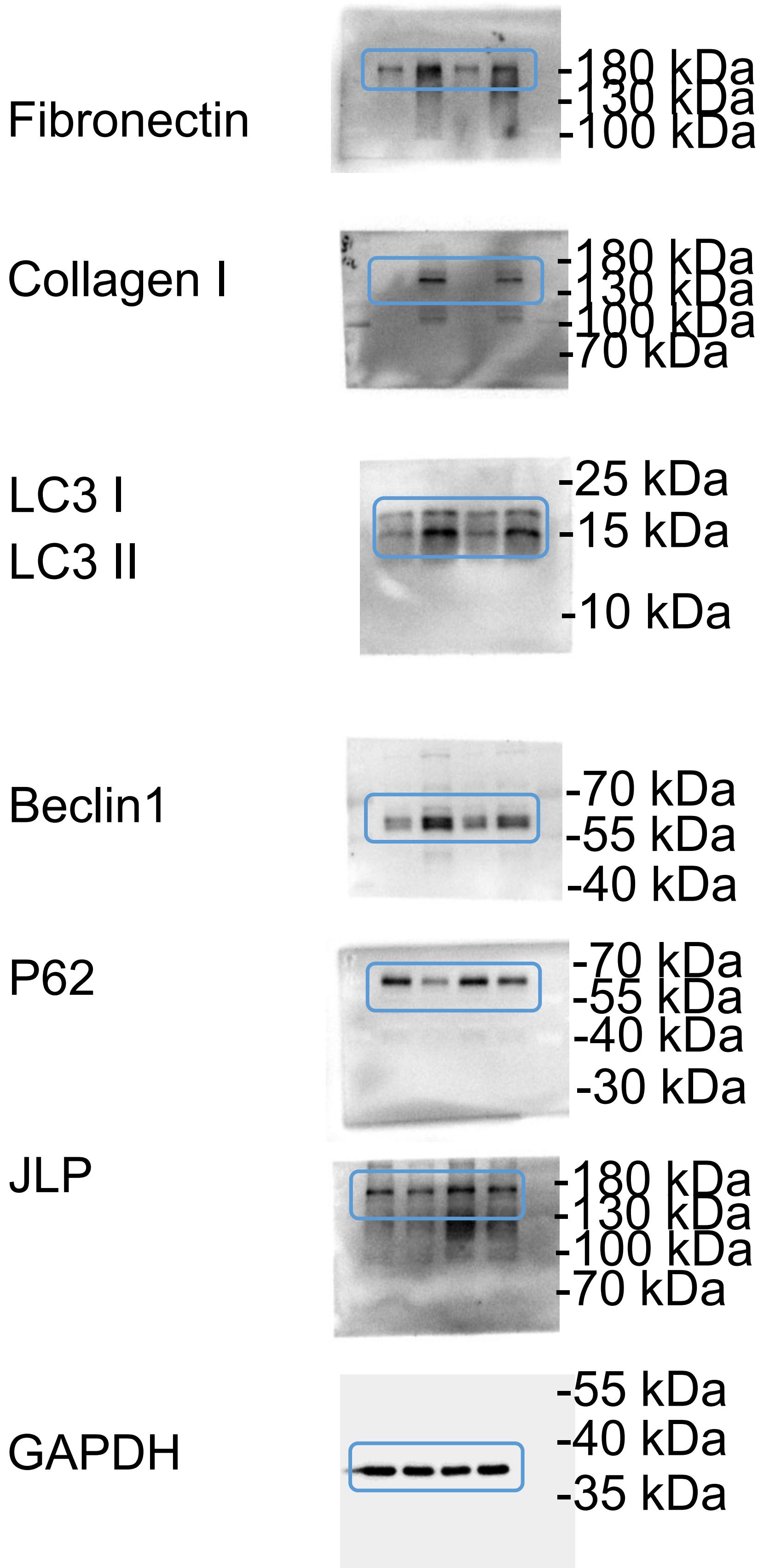

Full unedited gel for Figure S13G

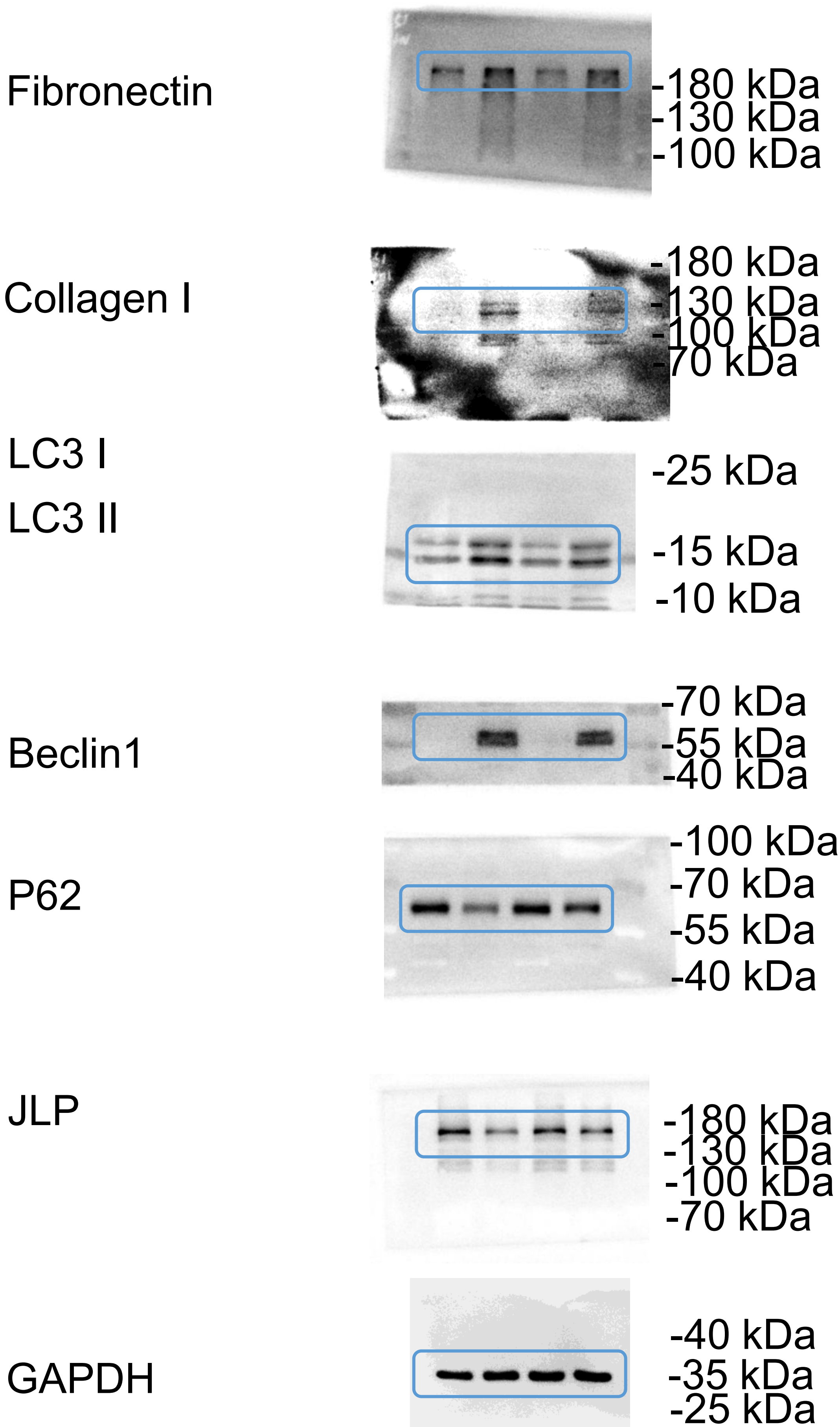

Supplement: Supplementary file 2 [file jciinsight-11-196835-s161.pdf]
